# Ex vivo study of molecular changes of stained teeth following hydrogen peroxide and peroxymonosulfate treatments

**DOI:** 10.1038/s41598-023-43201-y

**Published:** 2023-09-28

**Authors:** Paulo Wender P. Gomes, Simone Zuffa, Anelize Bauermeister, Andrés Mauricio Caraballo-Rodríguez, Haoqi Nina Zhao, Helena Mannochio-Russo, Cajetan Dogo-isonagie, Om Patel, Paloma Pimenta, Jennifer Gronlund, Stacey Lavender, Shira Pilch, Venda Maloney, Michael North, Pieter C. Dorrestein

**Affiliations:** 1 Skaggs School of Pharmacy and Pharmaceutical Sciences, Collaborative Mass Spectrometry Innovation Center, University of California, San Diego, La Jolla, CA USA; 2 Skaggs School of Pharmacy and Pharmaceutical Sciences, University of California, San Diego, La Jolla, CA USA; 3 Colgate-Palmolive, Global Technology Center, Piscataway, NJ USA

**Keywords:** Metabolomics, Mass spectrometry, Dentistry

## Abstract

White teeth can give confidence and tend to be associated with a healthier lifestyle in modern society. Therefore, tooth-bleaching strategies have been developed, including the use of hydrogen peroxide. Recently, peroxymonosulfate has been introduced as an alternative bleaching method to hydrogen peroxide. Although both chemicals are oxidizing agents, their effects on the molecular composition of the stained teeth are yet unknown. In this study, the molecular profiles of teeth bleached with hydrogen peroxide and peroxymonosulfate were compared using Liquid Chromatography-Tandem Mass Spectrometry. Statistical analyses were used to assess the samples. In addition, reference spectral libraries and in silico tools were used to perform metabolite annotation. Overall, principal component analysis showed a strong separation between control and hydrogen peroxide and peroxymonosulfate samples (*p* < 0.001). The analysis of molecular changes revealed amino acids and dipeptides in stained teeth samples after hydrogen peroxide and peroxymonosulfate treatments. Noteworthy, the two bleaching methods led to distinct molecular profiles. For example, diterpenoids were more prevalent after peroxymonosulfate treatment, while a greater abundance of alkaloids was detected after hydrogen peroxide treatment. Whereas non-bleached samples (controls) showed mainly lipids. Therefore, this study shows how two different tooth-whitening peroxides could affect the molecular profiles of human teeth.

## Introduction

White teeth are associated with self-confidence in modern society, and an increasing number of patients and consumers have demonstrated concern about the appearance and darkening^1^ of their teeth, despite that discoloration is a natural process and is usually caused by many factors, such as food, and beverages, smoking, microorganisms, mechanical wearing, and aging^2,3^. Thus, a significant amount of different aged populations have dissatisfaction with tooth color. For instance, a recent report revealed that 34% of Americans are unhappy with the color of their teeth^4^. Additionally, in another study conducted in the United Kingdom (UK) 96.6% of the interviewed people believed that the appearance of their teeth could affect their behavior and self-confidence^5^.

A tooth’s color is determined by a combination of factors, including the tooth's inner structure, the thickness of its enamel, and the presence of stains or discoloration on the surface^6^. The inner structure of a tooth is composed of a substance called dentin, which has a natural yellow hue. This color can show through the semi-translucent enamel, the outermost layer of the tooth, affecting the tooth's overall appearance^7^. The thickness and quality of the enamel can vary from person to person and are determined by such factors as age, genetics, and oral hygiene habits, making some teeth appear more yellow or gray. A person’s daily habits are major contributors to a tooth’s color. Stains from food, drinks, tobacco, and other substances can accumulate on the enamel surface, causing discoloration^8^. These are often referred to as extrinsically derived stains and are caused by highly colored molecules known as chromogens that reside in these consumables^9^. Common chromogens found in dark-colored food and beverages are tannins which are found in tea, coffee, red wine, and some fruits, and anthocyanins, which are found in many plants, including berries, red grapes, cherries, and red cabbage^10^. Stains that are derived from intrinsic sources include certain medications such as tetracycline or some antihistamines that are used during a tooth’s development or blood due to trauma^11^. These types of stains are often difficult to bleach.

Oxidizing agents are commonly used to bleach stains. Hydrogen peroxide (H_2_O_2_) and carbamide peroxide (CH_6_N_2_O_3_), also known as urea-hydrogen peroxide are oxidizing agents commonly used in oral care products designed for tooth whitening^12^. These molecules are highly reactive and are capable of oxidizing the chemical bonds responsible for the stain’s color, which reduces their ability to absorb visible light and hence appear less colored. In addition to these two oxidizing agents, ozone (O_3_) alone or combined with hydrogen peroxide is also widely used for teeth-bleaching treatments^13–15^. Ozone is a source of superoxide (highly reactive) and can provide additional hydroxyl groups when combined with hydrogen peroxide, improving the efficacy in the teeth bleaching effectiveness^15,16^. Tooth whitening products using hydrogen peroxide, carbamide peroxide, or ozone are available in many forms for both at-home use and professional whitening procedures. These product forms include toothpaste, mouthwash, gel base applications, strips, or gels applied in combination with a tray^17^.

Another very commonly used oxidizing agent is potassium peroxymonosulfate (KHSO_5_)^18^. Although this agent is a well-established cleanser and sanitizer as a result of its potent oxidizing power, it has not been utilized as a tooth-bleaching agent. Colgate-Palmolive’s research and development have developed toothpaste that utilizes peroxymonosulfate (MPS) as an alternative tooth-bleaching ingredient. However, it is unknown whether these different bleaching strategies could affect the molecules found in stained teeth, or even if these bleaching strategies have no significant difference in the impact on teeth molecules (null hypothesis). In this context, a primer investigation into the effects of bleaching strategies contributes to the broader understanding of dental materials and their impacts on the human teeth' molecular profile. Here, we investigate the impact of two teeth bleaching methods (H_2_O_2_ and MPS) on the molecules that could be detected from destained teeth post-bleaching via untargeted liquid chromatography followed by tandem mass spectrometry (LC–MS/MS), highlighting how the bleaching methods are affecting on the teeth metabolome.

## Results

**Figure 1:**
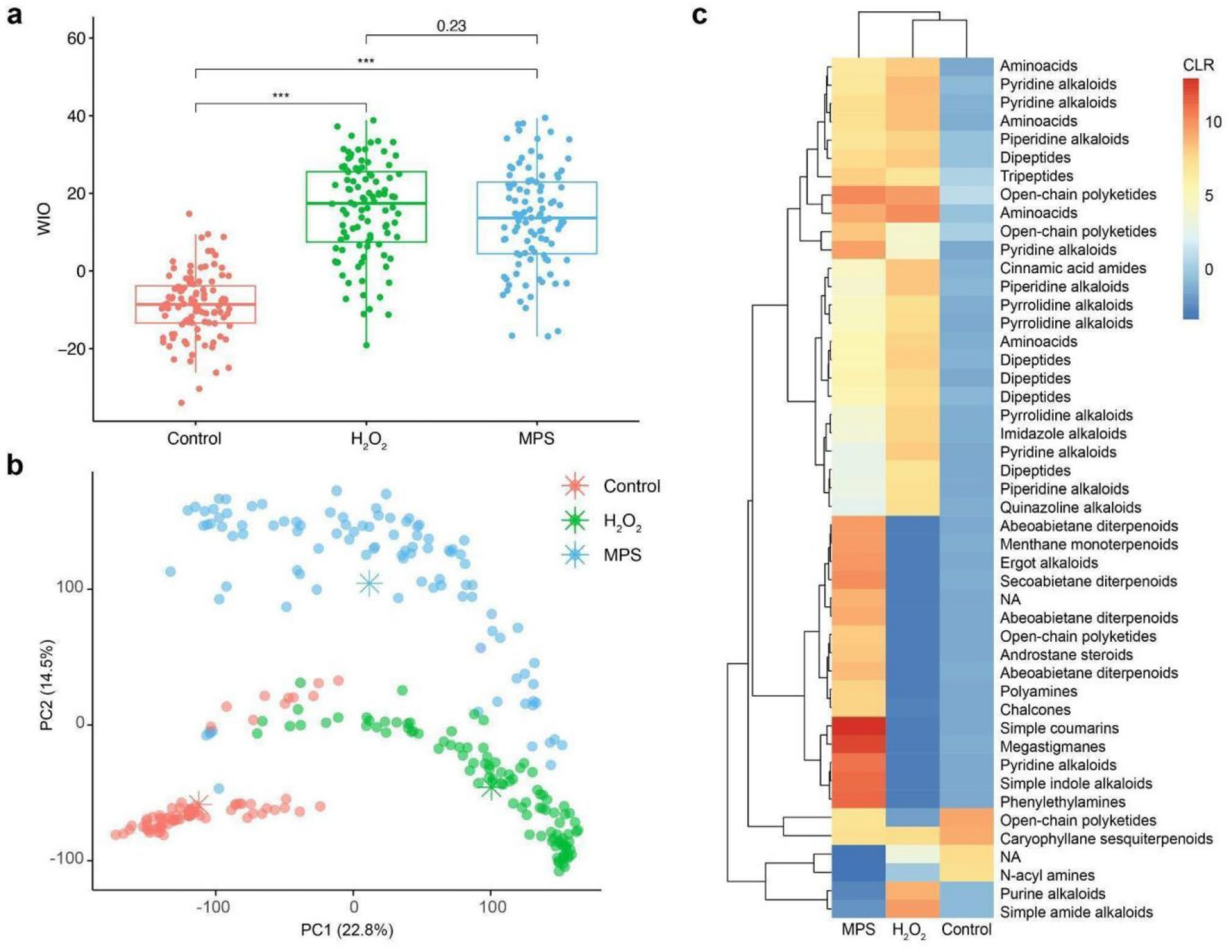
Bleaching methods significantly alter teeth' biochemical profiles. (**a**) Boxplots of non-normalized whiteness index (WIO) show a significant difference between Control and H_2_O_2_/MPS bleaching methods. This is a whiteness index based on human perception studies, − WIO are darker teeth and + WIO are whiter teeth^23^. Analysis of variance (ANOVA) followed by Tukey’s Honestly Significant Difference (HSD) Test. ***p < 0.05. (**b**) PCA shows a clear separation between control and bleaching methods according to the Permutational Analysis of Variance (PERMANOVA), R2 = 0.27, *p* < 0.001. (**c**) Heatmap of the top 47 features with VIP scores > 1.00 extracted from pairwise PLS-DA models. The asterisks in the scores plots represent group centroids.

Our results showed the average delta Whiteness Index (WIO)^19^ was statistically the same for both bleaching treatments (Fig. 1a). Large Cohen’s^20^ d values for Control vs. H_2_O_2_ (d estimate of − 2.312481) and for Control vs. MPS (− 2.034352) were observed (more details can be found in Supplementary Table 1). Otherwise, Cohen’s d value of 0.16995 for H_2_O_2_ vs. MPS indicates a negligible effect size. Unsupervised principal component analysis of the LC–MS/MS data showed a strong separation according to permutational analysis of variance (R2 = 0.27, *p* < 0.001) between control and bleached samples (Fig. 1b). Furthermore, the PCoA (Supplementary Fig. 1) gives insight into the metabolomic variation of the control and that treatment with either of the peroxides results in an entirely different metabolomics profile. Excitingly, this shows that the experimental treatment result is a much larger change in consistency than the original consistency of the samples. The low *p*-values 1.00 × 10^–92^ (PC1) and 3.35 × 10^–63^ (PC2) and large *F* statistics of 469.84 (PC1) and 242.72 (PC2) suggest that the differences among the groups are unlikely to have occurred by random chance. Pairwise partial least square discriminant analysis models (PLS-DA) (Supplementary Fig. 2) showed proper insights to understand which metabolites feature MS^1^ (i.e., a detected signal with *m/z* and retention time which correspond to a detected molecule) differed between groups A, B, and control. Features with variable importance projection (VIP) scores > 1.00 were considered relevant for group separation^21^. Thus, a total of 3315 mass spectrometry features presented a VIP > 1 (Supplementary Table 2). The top 25 discriminating features obtained from the pairwise PLS-DA models built on Control vs. H_2_O_2_ and Control vs. MPS (Supplementary Fig. 2a, b) are represented as a heatmap of the centered log ratio-transformed peak intensities (Fig. 1c). In this heatmap, the molecular class of these 25 tandem mass spectra (MS/MS) are described according to their ontology classification. These results correspond to chemical classes or level 3 annotations based on the Metabolomics Standards Initiative (MSI)^22^. Out of 47 features, six of them were less abundant in the MPS-treated samples compared to Control, one annotation included palmitoylethanolamide (Supplementary Fig. 3), while 20 of them were decreased in samples treated with H_2_O_2_ compared to Control, including polyketides, terpenoids, acyl amine, and alkaloids. Both treatments led to an increased abundance of features classified as amino acids, dipeptides, and pyrrolidine alkaloids. Differences between the MPS and H_2_O_2_ whitening treatments were also detected. Among the top 47 discriminant features, MPS and H_2_O_2_-treated teeth resulted in differences in features classified as di- and triterpenoids, open-chain polyketides (including hydroxylated fatty acids), and polyamines.

**Figure 2:**
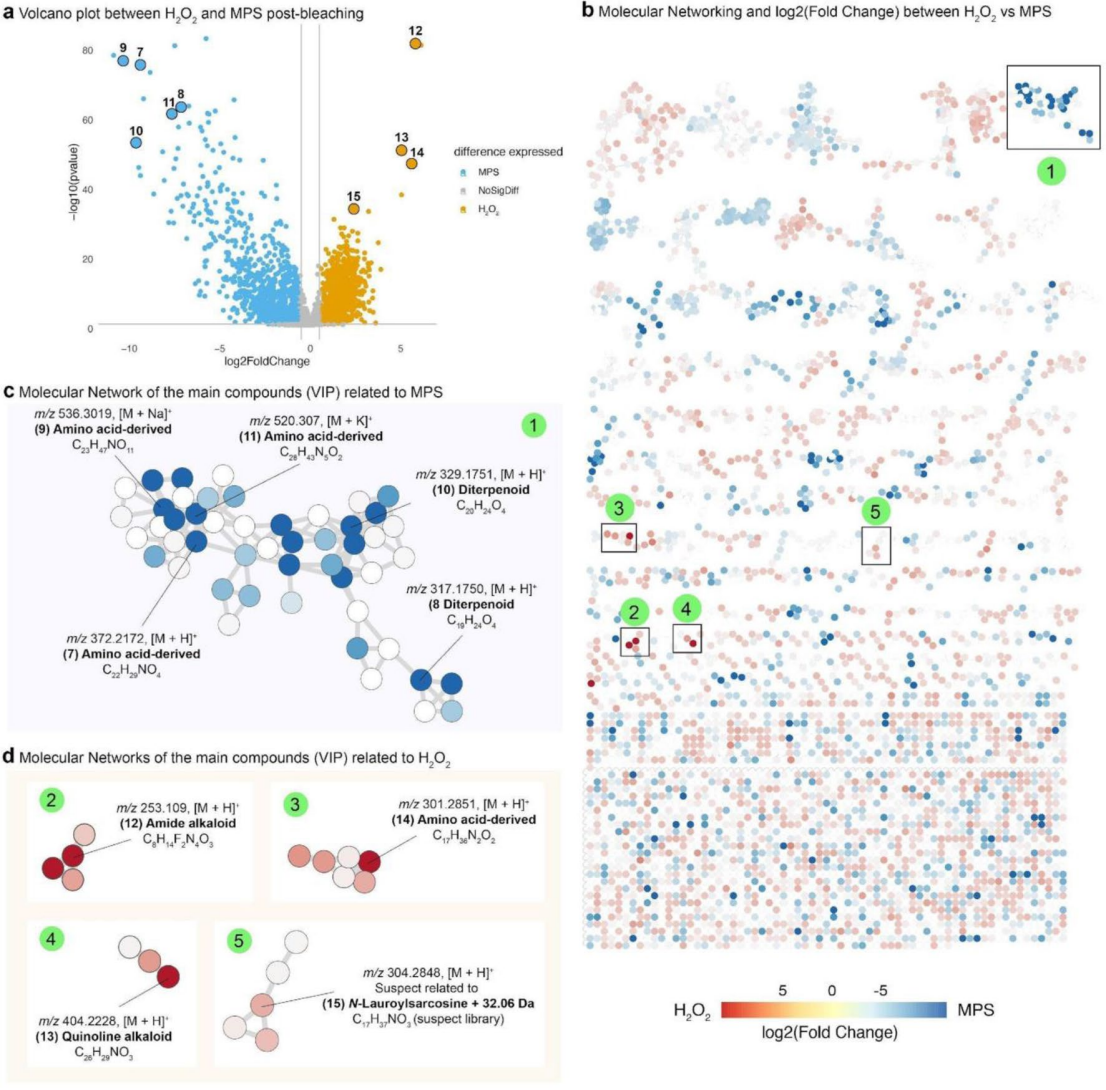
Univariate analyses and annotated metabolites in H_2_O_2_ and MPS treated samples. (**a**) Volcano plot between H_2_O_2_ and MPS treated samples and nine examples (7–15) among the higher VIP score metabolites related to each group of samples. (**b**) Molecular networking displaying log2 fold change of metabolite relative abundances: blue indicates metabolites present in high abundance after MPS treatment while red after H_2_O_2_ treatment, and numbers with green backgrounds (1–5) represent the highlighted networks in (**c**) and (**d**). (**c**) Chemical information on metabolites considered important in MPS post-bleaching predicted using a systematic classification of unknown metabolites (CANOPUS). (**d**) Information on metabolites considered important in H_2_O_2_ post-bleaching predicted via CANOPUS.

Univariate analysis provided the fold changes of metabolite relative abundances between H_2_O_2_ and MPS treatments, as presented by the volcano plot (Fig. 2a). MS features with fold changes greater than 0.6 and a p-value less than 0.05 were labeled as “H_2_O_2_” indicating an upregulated trend. Unlike that, features with fold changes less than − 0.6 and a p-value below 0.05 were assigned the label “MPS, ” signifying a downregulated pattern. These criteria resulted in 2,138 significant features (MPS and H_2_O_2_) vs. 903 non-significant ones. Comparisons between both the H_2_O_2_ and MPS treatments and the control group can be found in Supplementary Fig. 3. These changes were then visualized in the context of different ion forms, and once metabolites with similar structures present similar fragmentation patterns, the mass spectrometry results were also mapped in the form of molecular networks^24^, i.e., an approach that allows clustering by the similarity of a huge amount of MS/MS spectra obtained from metabolomics experiments (Fig. 2b). About 9.10% of the data was annotated, an acceptable percentage compared to a typical ~ 10% of other human matrices^25^. It is important to highlight that ~ 70% of them were annotated by the suspect library^26^, an MS/MS library to propagate annotation on Global Natural Products Social (GNPS). Additionally, SIRIUS software^27^ predicted chemical classes (Fig. 2c, d), enabling the assessment of similarities and differences in samples bleached with H_2_O_2_ and MPS, respectively. In both H_2_O_2_ and MPS-bleached samples, there was a higher relative abundance of nitrogen-based compounds compared to controls (*e.g.*, amino acids and derivatives, (Supplementary information Fig. 3). Among the selected metabolites based on VIP score, two terpenes-derived spectra were detected mainly in MPS samples (Fig. 2c). In contrast, the teeth bleached with H_2_O_2_ revealed a more prevalent presence of alkaloids (Fig. 2d).

## Discussion

Different bleaching strategies could affect the molecular profile of human teeth in different ways or even have no significant difference or impact on teeth molecules (null hypothesis). The answers to these hypotheses were still unknown until this primer study. In this context, statistical analyses showed a significant difference in molecular profiles between the samples after bleaching strategies, thus, the null hypothesis was rejected. Overall, we assessed if metabolomics can be used to evaluate the molecular changes in teeth after two different bleaching methods (H_2_O_2_ and MPS). Notice that enamel-based tooth stains are primarily extrinsic surface-based stains. Dentin is naturally darker and more yellow than enamel, dentin is exposed as enamel thins due to age-related wear-and-tear or inadequate oral hygiene, gum recession, and so on. To understand the overall effect bleaching has on all stain types both dentin and enamel were included. Specific studies focusing on enamel and dentin will require separate studies as not all surface bleaching can reach the dentin to provide intrinsic bleaching effects. Here both actives were chosen for their similarity in being able to reach the dentin.

As experimental values of tooth colors are an average of acceptable color, and there is considerable inconstancy in human teeth colors^28,29^ due to different lifestyle factors (diet, smoking, drinking, medications), we designed the experiment so that the effect of bleaching could be obtained from the exact same teeth. Notice dentin, a calcified tissue beneath the enamel, can exhibit different susceptibility to bleaching agents compared to enamel due to its distinct composition and structure. This variation in bleaching susceptibility could lead to uneven color changes across the tooth samples, potentially introducing variability in the metabolomic data. Thus, further research and analyses may be necessary to elucidate the precise role of dentin composition in the observed molecular variations and their relationship with the bleaching process. Overall, all sectioned teeth in three parts undergoing bleaching treatments, either H_2_O_2_ or MPS, were treated until they achieved a similar change in whiteness index (WIO). In addition, Cohen’s d value provided insights about the effect size using WIO values. Cohen’s d values observed for the Control vs. H_2_O_2_ group, and Control vs. MPS group imply a substantial and significant difference between these groups compared to the control. In both cases, the effect size was considerably large, despite negative values, since the magnitude (absolute value) of Cohen’s d indicates the strength of the effect, regardless of its direction. Larger absolute values of Cohen’s d suggest larger effect sizes, regardless of whether the value is positive or negative^30,31^. It confirms the results obtained from *p*-values, i.e., the effect of both bleaching methods is statistically significant. On the other hand, Cohen’s d value suggests a negligible effect size when comparing the H_2_O_2_ vs. MPS group. This indicates a very small difference between both groups in terms of power bleaching. Although important, the color should be considered only one out of many other aspects used to evaluate different teeth bleaching methods. For instance, it is essential to consider the potential for bleaching sensitivity or discomfort experienced during the procedure. Bleaching sensitivity, often referred to as tooth sensitivity, can be a significant concern for individuals undergoing tooth whitening treatments, especially those involving hydrogen peroxide and carbamide peroxides, since some individuals may experience tooth sensitivity or mild irritation to the gums when using these oxidizing agents^32^. In this context, a previous study in animal model suggests that in terms of sensitivity, pulp damage, or even inflammation symptoms caused by oxidizing agents, peroxymonosulfate may be lighter than other peroxides^33^. However, to have a complete understanding further studies are highly encouraged.

Compared to the non-bleached controls, more polar metabolites remained in H_2_O_2_ and MPS bleached teeth. The control samples revealed more lipid-like molecules compared to the bleached ones. A previous report that evaluated the exposome of deciduous teeth in prenatal and postnatal suggests the presence of lipophilic molecules or lipid-like could be attributed to tooth external exposures^34^. As teeth are not a common matrix subjected to chemical characterization, many metabolites detected in this study remain without reference spectra in the public domain, and thus, a comprehensive chemical characterization is still a hard task. However, the lipid-like characterized here are endogenous lipids, a chemical class well-known with established anti-inflammatory and anti-hyperalgesic properties^35^. Noteworthy, these metabolites were observed in controls but not in bleached teeth. Thus, bleaching procedures appeared to remove some of the lipophilic molecules. Although the mechanism of action of hydrogen peroxide-based methods remains partially unknown, peroxide reactions generate free hydroxyl radicals (2^•^OH). These radicals can oxidize the melanoidin pigments which cause stains in the teeth, introducing OH groups into many molecules and resulting in teeth whitening^4^. Although the role of such molecules in staining is still unknown, such lipophilic constituents are part of the pellicles of the teeth and can serve as hydrophobic bacterial binding sites, which can promote the formation of microbial biofilms on tooth surfaces^36,37^ and cause the demineralization of teeth by acids produced by cariogenic microorganisms^38–40^. Despite no direct evidence of a molecular mechanism, our results suggest that the treatments used could dissolve that lipid film, reducing the chances of generating biofilm or even bacterial proliferation.

Pyridine alkaloids, piperidine alkaloids, pyrrolidine alkaloids^41^, and amino acids, were the main chemical classes observed among the top 47 metabolites with high contribution to group separation. These amino acids were mainly present in samples bleached with MPS, and their relative abundances were significantly increased compared to the H_2_O_2_ treatment. Although the reason for this is unclear, tooth bleaching methods can change the abundance of proteins in the gingival crevicular fluid^42^, which accumulates in the necks of teeth^43^. Since proteins are built by blocks of amino acids, the use of whitening peroxide-based products could cause the hydrolysis of proteins^44^ present in teeth samples. This is consistent with the higher concentration of free amino acids and dipeptides after dental whitening treatments (Supplementary Fig. 4). The different amino acid-derived spectra observed for the H_2_O_2_ and MPS treatments indicate that even though they are both oxidizing agents, their action results in different molecular profiles, and thus react differently with the available molecular diversity in teeth samples. Furthermore, in quantitative terms, out of all the features used to create the volcano plot between H_2_O_2_ and MPS treatments, 54% of metabolites had fold change to H_2_O_2_, and 46% related to MPS.

These observations are consistent with the following hypothesis. First, our results showed that both bleaching treatments remove lipid-like molecules. Further, there is an increase in amino acid derivatives, consistent with the breakdown of proteins by oxidizing agents. The two different bleaching treatments resulted in distinct patterns of amino acid derivatives, likely due to different reactivities with different amino acids in proteins. As lipids and proteins are key adherents to teeth^45^, the observations support the hypothesis that the bleaching treatments break down proteins and lipids, including colored small molecules, or perhaps even the release of protein-bound metals that can give rise to stains. Notably, further studies are needed to further explore if the whitening treatments have additional benefits. In this regard, previous research reported that a single amino acid could inhibit bacterial growth^45^ in human teeth, and dipeptides were associated with repairing human tooth enamel^46^. Despite being the first metabolomics-based method for assessing the pre and post-bleaching dental molecular profile, the results suggest that there is a potential link between biofilm and staining. Finally, we have now established a new metabolomics method that enables the analysis of the molecular composition of teeth and the effects of destaining strategies.

## Conclusion

Despite the limitations of this study, it is possible to conclude that both H_2_O_2_ and MPS bleaching treatments induced substantial molecular alterations in teeth compared to non-bleached controls. These changes were characterized by a reduction in lipophilic molecules, which were associated with stains. Furthermore, both H_2_O_2_ and MPS caused an increase in amino acid derivatives in bleached teeth samples, suggesting the breakdown of proteins by the oxidizing agents. These observations support the hypothesis that the bleaching treatments not only target external stains but also impact the release of stain molecules, such as their impact on lipophilic molecules, which could come from biofilm present on the surface and voids of the tooth surface. Additional bleaching studies may explore the benefits of these bleaching methods, such as their impact on lipophilic molecules and tooth enamel repair.

## Limitations

This study described how two different teeth destaining methods could impact the molecular composition of human teeth. However, there are a number of limitations to consider when interpreting the results. While we aimed for consistent changes in whiteness index (WIO), by cutting the same teeth into three parts and then performing each section to treatment, there is an inherent color variability due to lifestyle factors that could introduce bias and lead to uneven color changes across the tooth samples. Additionally, focusing on just two bleaching methods does not cover all possible conditions including variations in agent concentrations and application methods that can influence outcomes. Here we did not separate enamel from dentin, rather assessed the average picture of the molecular changes, In future work the enamel and dentin could be separated as there will likely be differing responses to bleaching. In addition, it is not known if these results translate to teeth originating from different communities or countries of origin, especially those that have very different diet patterns. In general, the long-term effects of these molecular changes on tooth health and color remain unknown. As an in vitro study, caution should be exercised in generalizing the findings to real dental offices. Therefore, for broader applicability, there is a need to develop in situ analysis of tooth staining. Tools such as the Iknife^47^, MassSpec Pen^48^, and SpiderMass^49^ could maybe be future approaches to assess the effects on the metabolome of teeth in vivo.

## Materials and methods

### Chemicals

LC–MS grade acetonitrile and water 0.1% formic acid were used for LC–MS analyses (Fisher Chemical; Thermo Fisher Scientific, Geel, Antwerp, Belgium). For the extraction process, ethyl acetate (J.T. Baker; Avantor, Center Valley, PA, USA), methanol, isopropyl alcohol, and water were used (Fisher Chemical; Thermo Fisher Scientific, Geel, Antwerp, Belgium), all HPLC grade. Analytical grade chemicals and synthetic hydroxyapatite also were used for sample preparation (Sigma-Aldrich; Millipore Sigma, St. Louis, MO, USA).

### Sample case calculation

To detect a significant difference in the mean Whiteness Index (WIO) between two groups (Control group and group X), the sample case calculation involved a two-sample t-test. For our calculations, we assumed the mean WIO of the control group set at 50; the mean WIO of Group X: 45; the common standard deviation set at 8.00; the desired level of significance (alpha) set at 0.05; the desired statistical power set at 0.8 (80%); using a sample size calculator with the formula = 2 × (Zα/2 + Zβ)^2^ × σ^2^/Δ^2^. Where: Zα/2 corresponds to the critical value of the standard normal distribution for the chosen alpha (e.g., around 1.96 for alpha = 0.05). Zβ corresponds to the critical value of the standard normal distribution for the desired power (approximately 0.84 for a power of 0.8). σ is the common standard deviation. Δ is the difference in means. Thus, sample size per group = 2 × (1.96 + 0.84)^2^ × 8^2^/(50–45)^2^ ≈ 95. Considering practical factors, rounding up to 100 teeth per group was a reasonable choice. This ensured a solid sample size that meets both statistical requirements and real-world data collection considerations.

### Sampling

**Figure 3:**
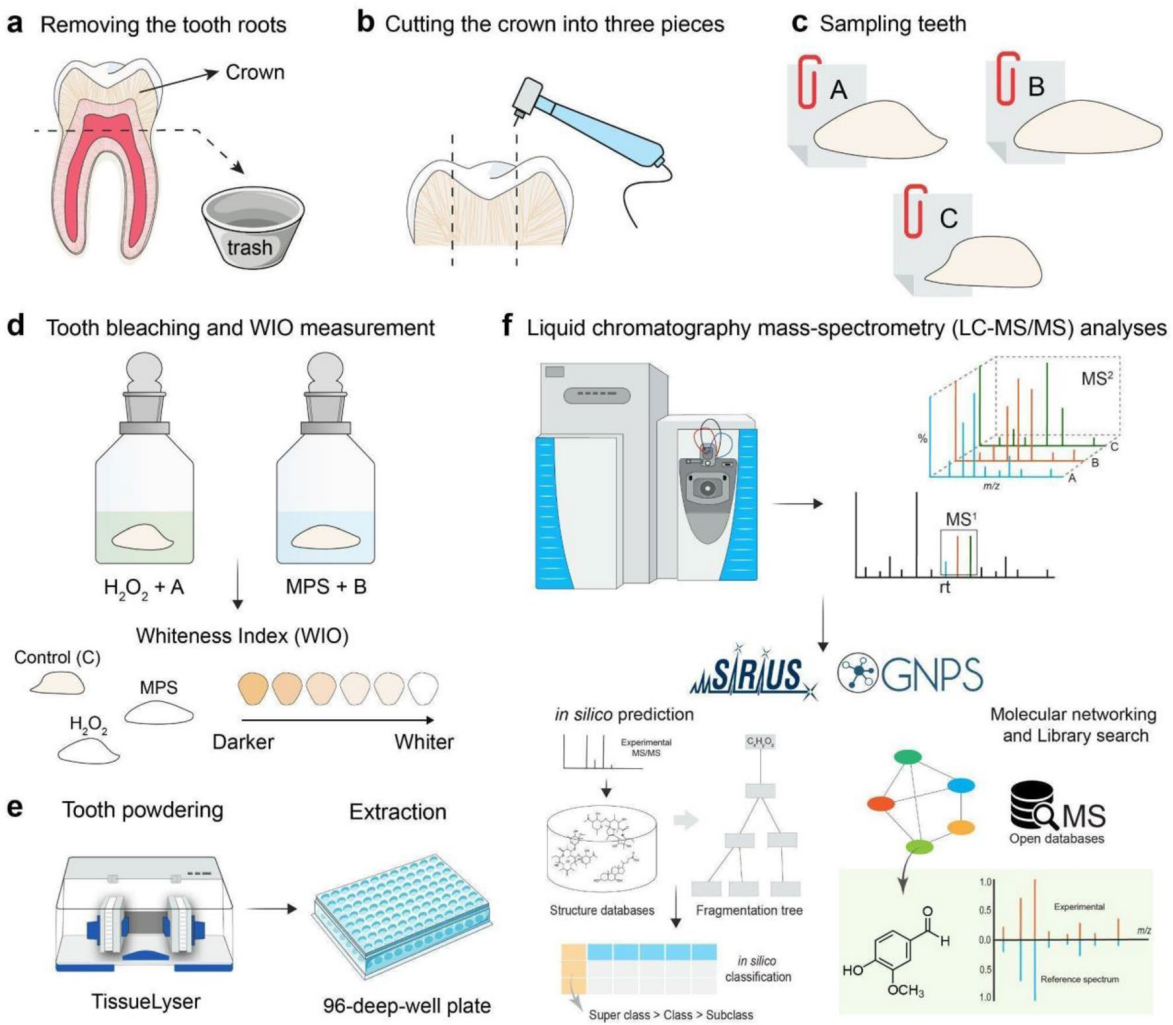
Overview teeth metabolome investigation. (**a**) The roots of the teeth were removed; (**b**) the crown was separated into three pieces; and (**c**) the samples randomized as the cut tooth sections were randomly added to bins A, B, and C, prior to destaining; (**d**) samples from groups A and B were bleached until their whiteness index (WIO) gave the same delta; (**e**) the samples were powdered in a TissueLyser and then, extracted using 96-deep-well plates; (**f**) LC–MS/MS obtained the molecular profiles for groups A, B, and control, and to understand the differences in the molecules detected, GNPS library search and in silico predictions via SIRIUS^27^ were used.

To evaluate the effects of the two different bleaching agents, hydrogen peroxide (H_2_O_2_) and peroxymonosulfate (MPS), 100 teeth were investigated in this study. Teeth were collected by Therametric (Therametric Technologies; Noblesville, IN, USA) under Institutional Review Board standards and are screened by them to exclude cracks, cavities, excess damage, excess staining, and so on. Once the teeth were provided, they were screened again to ensure that teeth appeared normal, undamaged, and large enough for analysis. The teeth were collected under HIPAA guidelines; thus, no patient information was collected. The teeth were stored for ~ 2 years at the time of processing. All teeth were provided in Thymol, a bacteriostatic agent, and they were always kept in Thymol at 4 °C (Celsius degrees) unless being treated. To understand the overall effect bleaching has on all stain types both dentin and enamel, cutting the teeth was needed. Figure 3 shows an overview of the workflow used. It includes a series of steps that were undertaken using teeth samples as a starting point. The experiment involved the assessment of changes in tooth whiteness and the corresponding metabolic modifications. The initial stage included the preparation of randomized. Tooth samples were cut into three random groups A, B, and C, where each group consisted of 100 teeth fragments (Fig. 3a–c), two of the teeth sections were subjected to either H_2_O_2_ or MPS neat treatments, and the bleaching of each tooth section was monitored before and after treatment with a Spectroshade Micro II (SpectroShade; Spectroshade USA, Oxnard CA, USA) to ensure they had similar changes in WIO levels (Fig. 3d)^15^. Baseline L*, a*, and b* values for all teeth were obtained using the Spectroshade Micro II via the CIE-Lab system, with the teeth oriented so color was measured through the enamel. The baseline values were then converted to XYZ values. X represents the reaction to the incident light on the retina, causing stimulation of the L cones (cones sensitive to longer wavelengths) within the human eye. Y indicates the luminance or brilliance of the given color. Z denotes the reaction to the light that triggers the S cones (cones sensitive to shorter wavelengths) in the human eye. Within this framework, the XYZ color space is frequently represented using chromaticity coordinates classified as x and y, where the relationship is defined by x = X/(X + Y + Z) and y = Y/(X + Y + Z)^50^. Thus, WIO was calculated as WIO = Y + 1075.012(x_n_−x) + 145.516 (y_n_−y), where (x, y) and (x_n_, y_n_) are the chromaticity coordinates of the sample and the reference whiteness index^23^. Lastly, all samples were stored in 0.1% thymol solution at 4 °C. Lastly, extracts produced from the grounded-up teeth, (Fig. 3e) resulted in LC–MS/MS data used in the metabolite annotation process (Fig. 3f).

### Sample preparation for metabolomics

All the samples were ground up using a TissueLyser (II QIAGEN; Redwood City, CA, USA) for 30 s at 30 Hz using metal jars (10 mL). The spoon and metal jar were sanitized with isopropyl alcohol:water (IPA:H_2_O) (1:1), and that procedure was performed between each sample. The teeth were stored in glass vials (5 mL) and then maintained at − 80 °C to avoid microbial growth before metabolomic analyses.

A total of 250 mg of each crushed tooth sample was plated for extraction in 96-well deep plates. In a single well, hydroxyapatite was used as the synthetic control of human teeth. Using a multichannel pipette, aliquots of 900 µL of MeOH:H_2_O:EtOAc (1:1:1) containing 1 mM of sulfadimethoxine (internal standard to extraction) were added at each 96-deep-well, and then the plates were vortexed (~ 10 s), sonicated for 90 min in an ultrasound bath, (Branson 2800; Branson, Danbury, CT, USA), and extracted overnight at 4 °C.

After the overnight extraction, the plates were centrifuged for 20 min at 2000 rpm and 4 °C. Thus, 600 µL of each sample were transferred to 2 mL 96-deep-well plates and dried in a speed vacuum concentrator. The samples were resuspended with 100 µL of MeOH:H_2_O (1:1) containing 1 mM sulfachloropyridazine (internal standard to metabolomic analyses). Then, the samples were vortexed for around 10 s, sonicated for 15 min, and centrifuged (2000 rpm) for 20 min (Sorvall; Thermo Fisher Scientific, Waltham, CA, USA). Lastly, using a multichannel pipette, aliquots of 80 µL of each sample were then transferred to the 200 µL ThermoScientific 96-well plate. A mix of MeOH:H_2_O (1:1) containing 1 mM sulfachloropyridazine was used as a blank, and a mix of sulfamethazine (C_12_H_14_N_4_O_2_S), sulfamethizole (C_9_H_10_N_4_O_2_S_2_), sulfachloropyridazine (C_10_H_9_ClN_4_O_2_S), sulfadimethoxine (C_12_H_14_N_4_O_4_S), amitriptyline (C_20_H_23_N*HCl), and coumarin-314 (C_18_H_19_NO_4_) was used as quality control (QC).

### Metabolomics analysis

The metabolomic analyses were performed in a Vanquish UHPLC system coupled to a Q-Exactive orbitrap mass spectrometer (Thermo Scientific; Thermo Fisher Scientific, Waltham, MA, USA), controlled by Thermo SII for Xcalibur software (Thermo Scientific; Thermo Fisher Scientific, Waltham, MA, USA). The chromatographic analysis was carried out on a C18 column, 50 × 2.1 mm, 1.7 μm particle size, 100 A pore size (Kinetex; Phenomenex, Torrance, CA, USA). A high-pressure binary system was used for gradient elution. The column and autosampler were kept at 40 and 25 °C, respectively. The flow rate was 0.5 mL/min, and the elution was carried out using ultra-pure water (solvent A) and acetonitrile (solvent B), both acidified with 0.1% of formic acid (FA). The gradient method was set as follows: 0–0.5 min, 5% B; 0.5–8.0 min, 5–100% B; 8.0–11.0 min, 100% B; 11.0–12.0 min, 100–5% B; and lastly, 12.0–14.0 min 5% B to stabilize the system before the subsequent analysis.

For mass spectrometry analyses, data-dependent acquisition (DDA) was used in an *m/z* range from 80 to 2000 Da with an electrospray source operating in the positive ionization mode. Before data acquisition, the sodium formate solution (Fisher Chemical; Thermo Fisher Scientific, Geel, Antwerp, Belgium) was used for external calibration with an error rate of less than 0.5 ppm. The spray voltage was set to 3.5 kV, sheath N_2_ gas pressure was set to 35 psi, and auxiliary N_2_ gas pressure was set to 10 psi. The ionization source was kept at 270 °C, 60 V S-lens RF level was applied, and the auxiliary gas heater was kept at 440 °C. Full scan MS^1^ was performed at 1.0 × 10^6^ with a resolution of 35,000 and a maximum ion injection of 100 ms. MS^2^ experiments were performed with a resolution of 17,500 with a max IT time of 60 ms, and topK6 was used for the 6 most abundant precursor ions per MS^1^. The MS^2^ precursor isolation window was set to 2 Da with an offset of 0.5 Da. The normalized collision energy (NCE) was set to a ramp from 20 to 40 eV, and the exclusion (MS^1^ and MS^2^) for unassigned ion charge states were set to 5 S-Lens, as well as isotope peaks.

### Data processing

The LC–MS/MS data were converted from RAW standard data format (Thermo Scientific; Thermo Fisher Scientific, Waltham, MA, USA) to mzML format using MSConvert 3.0.2^51^. Feature extraction of the resulting file was processed using MZmine 3.1.0^52^. The mass detection of MS^1^ and MS^2^ levels was performed using a signal noise of 1.0 × 10^5^ and 5.0 × 10^3^, respectively. The ADAP chromatogram builder was used to build the chromatogram, and a minimum group size of scans was set to 3, the minimum intensity of the group to 1.0 × 10^5^, and the highest to 3.0 × 10^5^ with an *m/z* tolerance of 10 ppm. The ADAP resolver module (wavelets) was applied to chromatographic deconvolution, and then, intensity window S/N was used as an S/N estimator with a signal-to-noise ratio set to 10, a minimum feature height of 1.0 × 10^4^, a coefficient of peak area of 1.70, a peak duration from 0.05 to 2.0 min and an RT wavelet range used to build a matrix of coefficients from 0.05 to 0.10 min. The isotope peak grouper module was applied to detect the isotopes with an *m/z* and RT tolerance of 10.0 ppm, and 0.2 min (charge 1 was used as standard) for the most intense isotope. To remove the duplicate features and aligner, the same *m/z* and RT tolerances were used, and the weight for *m/z* and RT was set to 3:1, respectively. The resulting peak list was then filtered to remove features from the blanks eventually and just features with isotope patterns and MS^2^ spectra associated were kept. Thus, a filtered peak list containing 4,304 features was exported as a .mgf and a .csv file containing feature information, which posteriorly was used for statistical analysis in RStudio(R) (RStudio; Posit, Boston, MA, USA)^53^, and MetaboAnalyst 5.0^54^.

### Feature-based molecular networking workflow

The output files (.mgf and .csv) obtained from MZmine 3 were then used in the Feature-Based Molecular Networking workflow^24^ on the GNPS platform (https://gnps.ucsdhttps://gnps.ucsd.edu.edu). The tolerances for the precursor ion mass (MS^1^) and the MS^2^ fragment ion were set to 0.02 and 0.02 Da, respectively. An edge was created when the MS/MS spectral comparisons had a cosine of 0.6 or greater with a minimum of four fragment ion matches to create the molecular network. The network topK was set to 10, maximum connected component size was set to 100. All spectra contained in the molecular networks were compared to the reference spectra available in the GNPS spectral libraries^55^, and a cosine of 0.6 and a minimum of 3 MS^2^ matches were applied. The suspect library^26^ was also used, and the job can be found at the following link, https://gnps.ucsd.edu/ProteoSAFe/status.jsp?task=4d51cce527d84e90aaa8f0f2799b7036. The results obtained from the univariate analysis described in data analysis section were used to create the molecular networks in Cytoscape software^56^ based on the fold change of each MS feature.

### Data analysis

Statistical analyses and the charts (Boxplots, PCA, heatmap, volcano plots) were performed on R version 4.0.2 (R Foundation for Statistical Computing, Vienna, Austria) and MetaboAnalyst 5.0^54^. Differences in teeth whiteness were tested with one-way ANOVA followed by the Tukey HSD test. The effect size was measured using Cohen’s d metrics^20^. Multivariate analysis was performed using the ‘mixOmics’ package^57^. Data was center log-ratio transformed before dimensionality reduction. Centroid separation was tested with PERMANOVA using the ‘vegan’ package. The performance of the pairwise PLS-DA models was calculated with four-fold cross-validation. For univariate analysis, a volcano plot generated on metabolite relative abundances was used to compare the two bleaching methods (Fold changes ± 0.6 and a *p*-value < 0.05 were). FDR was set to 0.05. CANOPUS^58^ and ClassyFire^59^ were used into SIRIUS^27^ for the predict chemical classes.

### Supplementary Information

Supplementary Information.

## Data Availability

The raw data used in this study are publicly available online at MassIVE (https://massive.ucsd.edu/) under the accession MSV000089540^60^. All data generated or analyzed during this study are included in this published article (and its Supplementary Information Files).

## Code availability

Data and code to generate multivariate models (PCA and PLS-DA) and heatmap are available on GitHub (https://github.com/simonezuffa/Manusript_Colgate).

## References

[1] Joiner A, Luo W (2017). Tooth colour and whiteness: A review. J. Dent..

[2] Abou-Neel EA (2016). Demineralization-remineralization dynamics in teeth and bone. Int. J. Nanomed..

[3] Alqahtani MQ (2014). Tooth-bleaching procedures and their controversial effects: A literature review. Saudi Dent. J..

[4] Kim D-H (2022). Nanoparticles as next-generation tooth-whitening agents: Progress and perspectives. ACS Nano.

[5] Hafidh, H. *How Dental Aesthetics Affects Self-Confidence and its Impact on Psychosocial Behaviour: A Clinical Study Within the University Of Leeds Ages* 18–25. https://lsmu.lt/cris/bitstream/20.500.12512/101443/1/hannan%20thesis%20final.pdf (2019).

[6] Begum Z, Chheda P, Shruthi CS, Sonika R (2014). Effect of ceramic thickness and luting agent shade on the color masking ability of laminate veneers. J. Indian Prosthodont. Soc..

[7] Goldberg M (2011). Dentin structure composition and mineralization. Front. Biosci. (Elite Ed.).

[8] Sawai M, Bhardwaj A, Jafri Z, Sultan N, Daing A (2015). Tooth polishing: The current status. J. Indian Soc. Periodontol..

[9] Sarembe S, Kiesow A, Pratten J, Webster C (2022). The impact on dental staining caused by beverages in combination with chlorhexidine digluconate. Eur. J. Dent..

[10] Mattioli R, Francioso A, Mosca L, Silva P (2020). Anthocyanins: A comprehensive review of their chemical properties and health effects on cardiovascular and neurodegenerative diseases. Molecules.

[11] Wang J, Zou D, Li Y, Liu P, Guo C (2023). Drug-induced tooth discoloration: An analysis of the us food and drug administration adverse event reporting system. Front. Pharmacol..

[12] Féliz-Matos L, Hernández LM, Abreu N (2014). Dental bleaching techniques; hydrogen-carbamide peroxides and light sources for activation, an update. Mini-review article. Open Dent. J..

[13] Aykut-Yetkiner A (2017). Color assessment after bleaching with hydrogen peroxide versus ozone: A randomized controlled clinical trial. Gen. Dent..

[14] AL-Omiri MK, Abul-Hassan RS, AlZarea BK, Lynch E (2016). Comparison of dental bleaching effects of ozone and hydrogen peroxide: An ex vivo study. Am. J. Dent..

[15] Al-Omiri MK, Al Nazeh AA, Kielbassa AM, Lynch E (2018). Randomized controlled clinical trial on bleaching sensitivity and whitening efficacy of hydrogen peroxide versus combinations of hydrogen peroxide and ozone. Sci. Rep..

[16] Bocci VA (2006). Scientific and medical aspects of ozone therapy, state of the art. Arch. Med. Res..

[17] Eachempati P, Kumbargere-Nagraj S, Kiran-Kumar-Krishanappa S, Gupta P, Yaylali IE (2018). Home-based chemically-induced whitening (bleaching) of teeth in adults. Cochrane Libr..

[18] Li Y (2023). Evaluation of oral and perioral irritation and sensitization potential of a whitening gel and a whitening toothpaste containing potassium monopersulfate. Am. J. Dent..

[19] del Pérez MM (2016). Development of a customized whiteness index for dentistry based on cielab color space. Dent. Mater..

[20] Lachenbruch PA, Cohen J (1989). Statistical power analysis for the behavioral sciences. J. Am. Stat. Assoc..

[21] Rangel-Huerta OD (2022). Metabolomics workflow for quality control of differently-processed pre-cooked chicken fillets. Food Chem..

[22] Sumner LW (2007). Proposed minimum reporting standards for chemical analysis chemical analysis working group (cawg) metabolomics standards initiative (msi). Metabolomics.

[23] Luo W, Westland S, Ellwood R, Pretty I, Cheung V (2009). Development of a whiteness index for dentistry. J. Dent..

[24] Nothias L-F (2020). Feature-based molecular networking in the gnps analysis environment. Nat. Methods.

[25] Gauglitz JM (2022). Enhancing untargeted metabolomics using metadata-based source annotation. Nat. Biotechnol..

[26] Bittremieux W (2022). Open access repository-scale propagated nearest neighbor suspect spectral library for untargeted metabolomics. BioRxiv.

[27] Dührkop K (2019). Sirius 4: A rapid tool for turning tandem mass spectra into metabolite structure information. Nat. Methods.

[28] Melgosa M (2020). Color inconstancy of natural teeth measured under white light-emitting diode illuminants. Dent. Mater..

[29] Paravina RD (2015). Color difference thresholds in dentistry. J. Esthet. Restor. Dent..

[30] Blanar CA, Munkittrick KR, Houlahan J, MacLatchy DL, Marcogliese DJ (2009). Pollution and parasitism in aquatic animals: A meta-analysis of effect size. Aquat. Toxicol..

[31] Cardi V, Leppanen J, Treasure J (2015). The effects of negative and positive mood induction on eating behaviour: A meta-analysis of laboratory studies in the healthy population and eating and weight disorders. Neurosci. Biobehav. Rev..

[32] Kielbassa AM, Maier M, Gieren AK, Eliav E (2015). Tooth sensitivity during and after vital tooth bleaching: A systematic review on an unsolved problem. Quintessence Int..

[33] Yang S, Sui B, Liu X, Sun J, Wang J (2022). A novel tooth bleaching gel based on peroxymonosulfate/polyphosphates advanced oxidation process: Effective whitening avoiding pulp damage and sensitivity. Chem. Eng. J..

[34] Yu M (2021). Tooth biomarkers to characterize the temporal dynamics of the fetal and early-life exposome. Environ. Int..

[35] Petrosino S (2018). Oral ultramicronized palmitoylethanolamide: Plasma and tissue levels and spinal anti-hyperalgesic effect. Front. Pharmacol..

[36] Hannig C, Hannig M (2009). The oral cavity—a key system to understand substratum-dependent bioadhesion on solid surfaces in man. Clin. Oral Investig..

[37] Kensche A, Reich M, Kümmerer K, Hannig M, Hannig C (2013). Lipids in preventive dentistry. Clin. Oral Investig..

[38] Slomiany BL (1986). Tooth surface-pellicle lipids and their role in the protection of dental enamel against lactic-acid diffusion in man. Arch. Oral Biol..

[39] Nomura R (2020). Contribution of severe dental caries induced by streptococcus mutans to the pathogenicity of infective endocarditis. Infect. Immun..

[40] Donadio G (2022). Diterpenoid constituents of psiadia punctulata and evaluation of their antimicrobial activity. J. Nat. Prod..

[41] Eerkens JW (2018). Dental calculus as a source of ancient alkaloids: Detection of nicotine by lc-ms in calculus samples from the Americas. J. Archaeol. Sci. Rep..

[42] Colares VLP (2019). Hydrogen peroxide-based products alter inflammatory and tissue damage-related proteins in the gingival crevicular fluid of healthy volunteers: A randomized trial. Sci. Rep..

[43] Challacombe, S. J., Shirlaw, P. J. & Thornhill, M. H. Chapter 102—immunology of diseases of the oral cavity. In *Mucosal Immunology (Fourth Edition)* (eds. Mestecky, J. *et al*.) 1943–1983 (Academic Press, 2015).

[44] Ailes I, Tohidi D, Ngo L, Keenan K (2019). Effect of hydrogen peroxide on hydrolysis of proteins. FASEB J..

[45] Kolderman E (2015). L-arginine destabilizes oral multi-species biofilm communities developed in human saliva. PLoS ONE.

[46] Mukherjee K, Ruan Q, Liberman D, White SN, Moradian-Oldak J (2016). Repairing human tooth enamel with leucine-rich amelogenin peptide–chitosan hydrogel. J. Mater. Res..

[47] Balog J (2010). Identification of biological tissues by rapid evaporative ionization mass spectrometry. Anal. Chem..

[48] Zhang J (2017). Nondestructive tissue analysis for ex vivo and in vivo cancer diagnosis using a handheld mass spectrometry system. Sci. Transl. Med..

[49] Fatou B (2016). In vivo real-time mass spectrometry for guided surgery application. Sci. Rep..

[50] Pan Q, Westland S (2018). Tooth color and whitening—digital technologies. J. Dent..

[51] Chambers MC (2012). A cross-platform toolkit for mass spectrometry and proteomics. Nat. Biotechnol..

[52] Schmid R (2023). Integrative analysis of multimodal mass spectrometry data in mzmine 3. Nat. Biotechnol..

[53] Racine JS (2012). Rstudio: A platform-independent ide for r and sweave. J. Appl. Econom..

[54] Pang Z (2022). Using metaboanalyst 5.0 for lc–hrms spectra processing, multi-omics integration and covariate adjustment of global metabolomics data. Nat. Protoc..

[55] Wang M (2016). Sharing and community curation of mass spectrometry data with global natural products social molecular networking. Nat. Biotechnol..

[56] Shannon P (2003). Cytoscape: A software environment for integrated models. Genome Res..

[57] Lê-Cao K-A, Welham Z (2021). Multivariate Data Integration Using R: Methods and Applications with the Mixomics Package.

[58] Dührkop K (2021). Systematic classification of unknown metabolites using high-resolution fragmentation mass spectra. Nat. Biotechnol..

[59] DjoumbouFeunang Y (2016). Classyfire: Automated chemical classification with a comprehensive, computable taxonomy. J. Cheminform..

[60] Dorrestein PC (2022). Massive msv000089540—gnps—phase 3_300 human teeth samples. Datasett.

