# Supplementary material for: Ex vivo study of molecular changes of stained teeth following hydrogen peroxide and peroxymonosulfate treatments
Source: Sci Rep. 2023 Sep 28;13:16349. doi: 10.1038/s41598-023-43201-y (PMC10539445; doi:10.1038/s41598-023-43201-y)
Supplement: Supplementary file 1 — Supplementary Information. [file 41598_2023_43201_MOESM1_ESM.docx]

***Ex Vivo* Study of Molecular Changes of Stained Teeth Following Hydrogen Peroxide and Peroxymonosulfate Treatments**

**Supplementary Information**

**Paulo Wender P. Gomes^1,2^, Simone Zuffa^1,2^, Anelize Bauermeister^1,2^, Andrés Mauricio Caraballo-Rodríguez^1,2^, Haoqi Nina Zhao^1,2^, Helena Mannochio-Russo^1,2^, Cajetan Dogo-isonagie^3^, Om Patel^3^, Paloma Pimenta^3^, Jennifer Gronlund^3^, Stacey Lavender^3^, Shira Pilch^3^, Venda Maloney^3^, Michael North^3^, Pieter C. Dorrestein^1,2*^**

^1^Collaborative Mass Spectrometry Innovation Center, Skaggs School of Pharmacy and Pharmaceutical Sciences, University of California, San Diego, La Jolla, CA, USA

^2^Skaggs School of Pharmacy and Pharmaceutical Sciences, University of California, San Diego, La Jolla, CA, USA

^3^Colgate-Palmolive, Global Technology Center, Piscataway, NJ, USA

^*^To whom correspondence should be addressed: pdorrestein@health.ucsd.edu

**content**

- **Supplementary Figure 1. Principal Coordinates Analysis (PCoA).** The PCoA shows a clear separation between control and distaining methods according to the Analysis of Variance (ANOVA), *F* = 469.84 (PC1) and 242.72 (PC2), *p* < 0.001.
- **Supplementary Figure 2. Different tooth bleaching strategies lead to distinct tooth biochemical profiles**. Pairwise PLS-DA models constructed on the CLR transformed feature table show close to perfect separation between control and different treatments (a,b,c). Scores plot of control vs. MPS (b) reveals possible misclassification of MPS samples. Model performances were calculated using random 4-fold cross-validation. The asterisk in the scores plots represents group centroids.
- **Supplementary Figure 3. Unpaired univariate analyses and annotated metabolites in control samples**. a) Volcano plot between control/H_2_O_2_ and selected metabolite (1) among the VIP metabolites that had matches against the reference MS/MS GNPS library. b) Volcano plot between control/MPS and selected metabolites (2-6) among the VIP metabolites that had matches against the reference MS/MS GNPS library. c) Mirror plot between experimental and reference spectrum of the metabolites that were annotated using GNPS library.
- **Supplementary Figure 4. Molecular family of consistent features of amino acid-derived detected in H_2_O_2_/MPS.** a) Annotated amino acid derived in both bleached samples; b) Each annotated metabolite has a mirror plot comparing a library MS/MS spectrum (top) and the teeth metabolite MS/MS spectrum (bottom) based on cosine similarity. Proposed chemical names and structures were listed when available.
- **Supplementary Table 1. Effect size results**: **Cohen’s d estimate values.** a) Cohen’s d estimate for Control vs. H_2_O_2_. b) Cohen’s d estimate Control vs. MPS. c) Cohen’s d estimate for H_2_O_2_ vs. MPS.
- **Supplementary Table 2. Features with variable importance projection (VIP).** A total of 3,315 mass spectrometry features presented a VIP > 1.00.


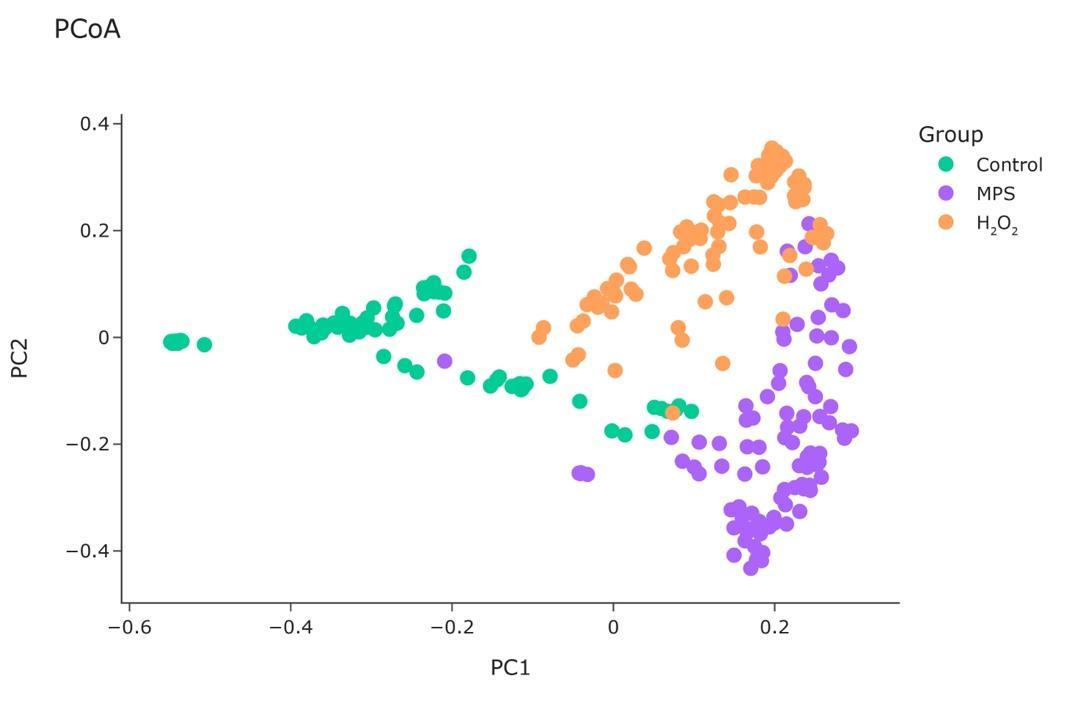


**Supplementary Figure 1. Principal Coordinates Analysis (PCoA).** The PCoA shows a clear separation between control and distaining methods according to the Analysis of Variance (ANOVA), *F* = 469.84 (PC1) and 242.72 (PC2), *p* < 0.001.

**
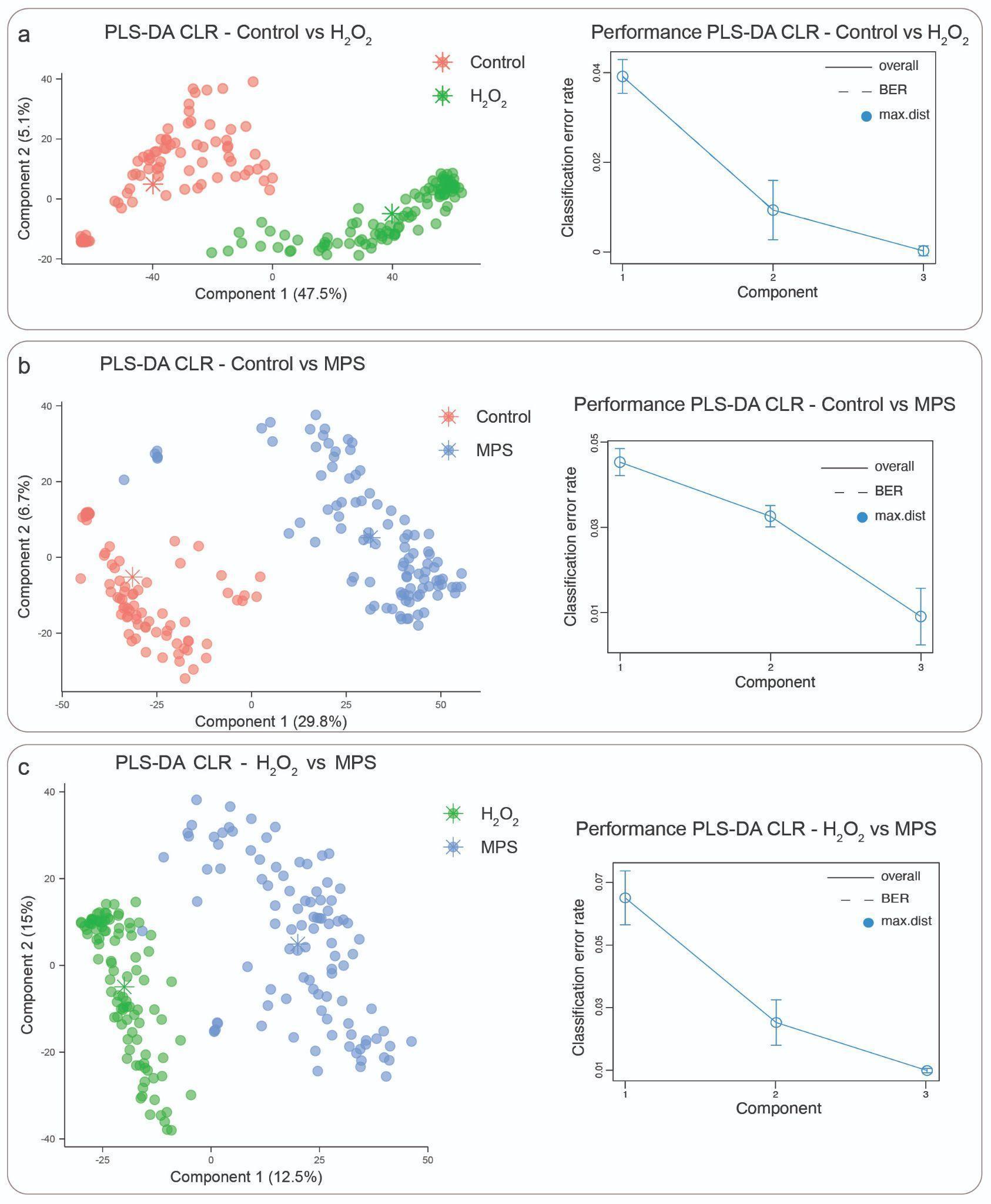
**

**Supplementary Figure 2**. **Different tooth bleaching strategies lead to distinct tooth biochemical profiles**. Pairwise PLS-DA models constructed on the CLR transformed feature table show close to perfect separation between control and different treatments (a,b,c). Scores plot of control vs. MPS (b) reveals possible misclassification of MPS samples. Model performances were calculated using random 4-fold cross-validation. The asterisk in the scores plots represents group centroids.

**
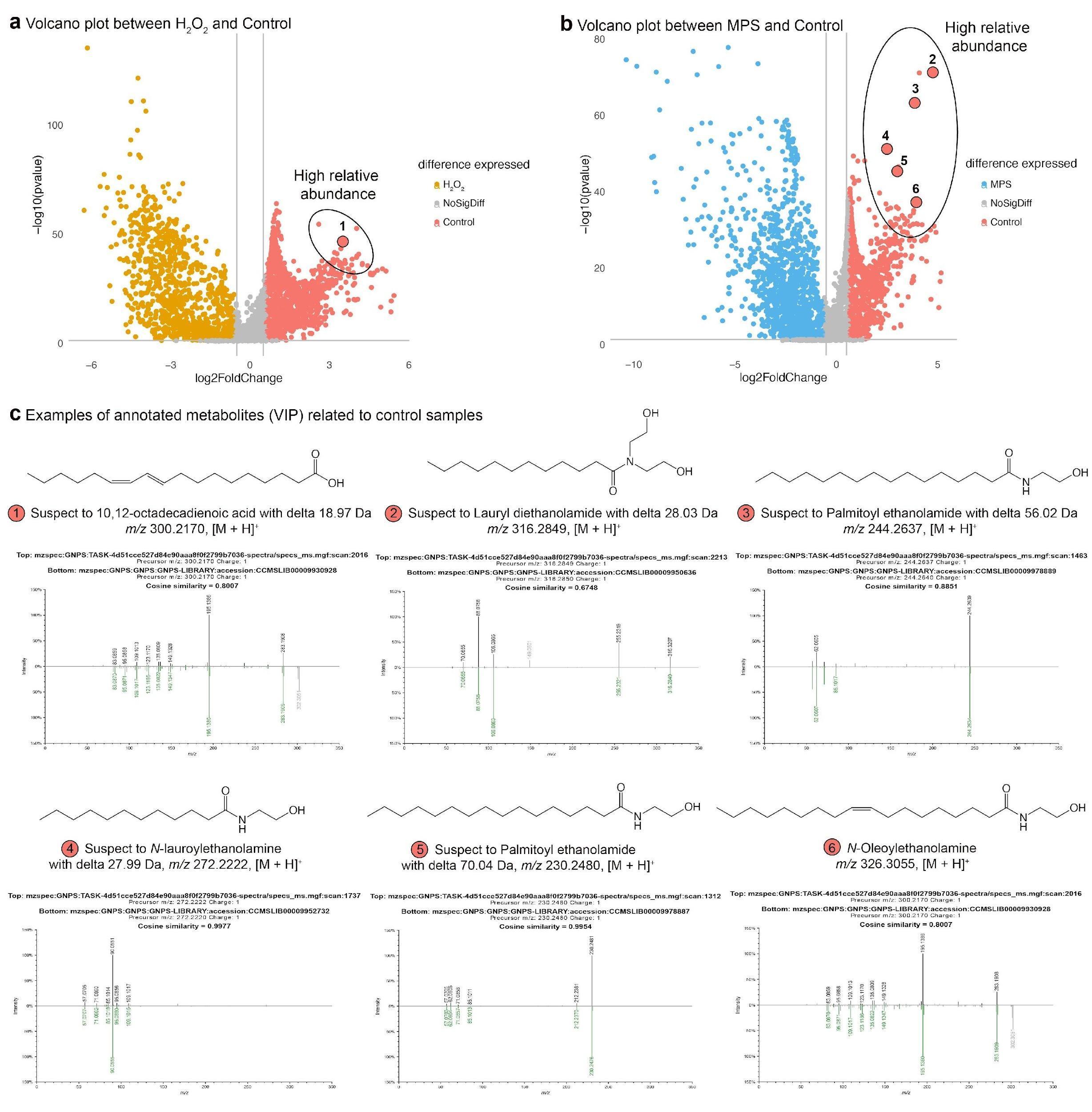
**

**Supplementary Figure 3. Unpaired univariate analyses and annotated metabolites in control samples.** a) Volcano plot between control/H_2_O_2_ and selected metabolite (1) among the VIP metabolites that had matches against the reference MS/MS GNPS library. b) Volcano plot between control/MPS and selected metabolites (2-6) among the VIP metabolites that had matches against the reference MS/MS GNPS library. c) Mirror plot between experimental and reference spectrum of the metabolites that were annotated using GNPS library.

**
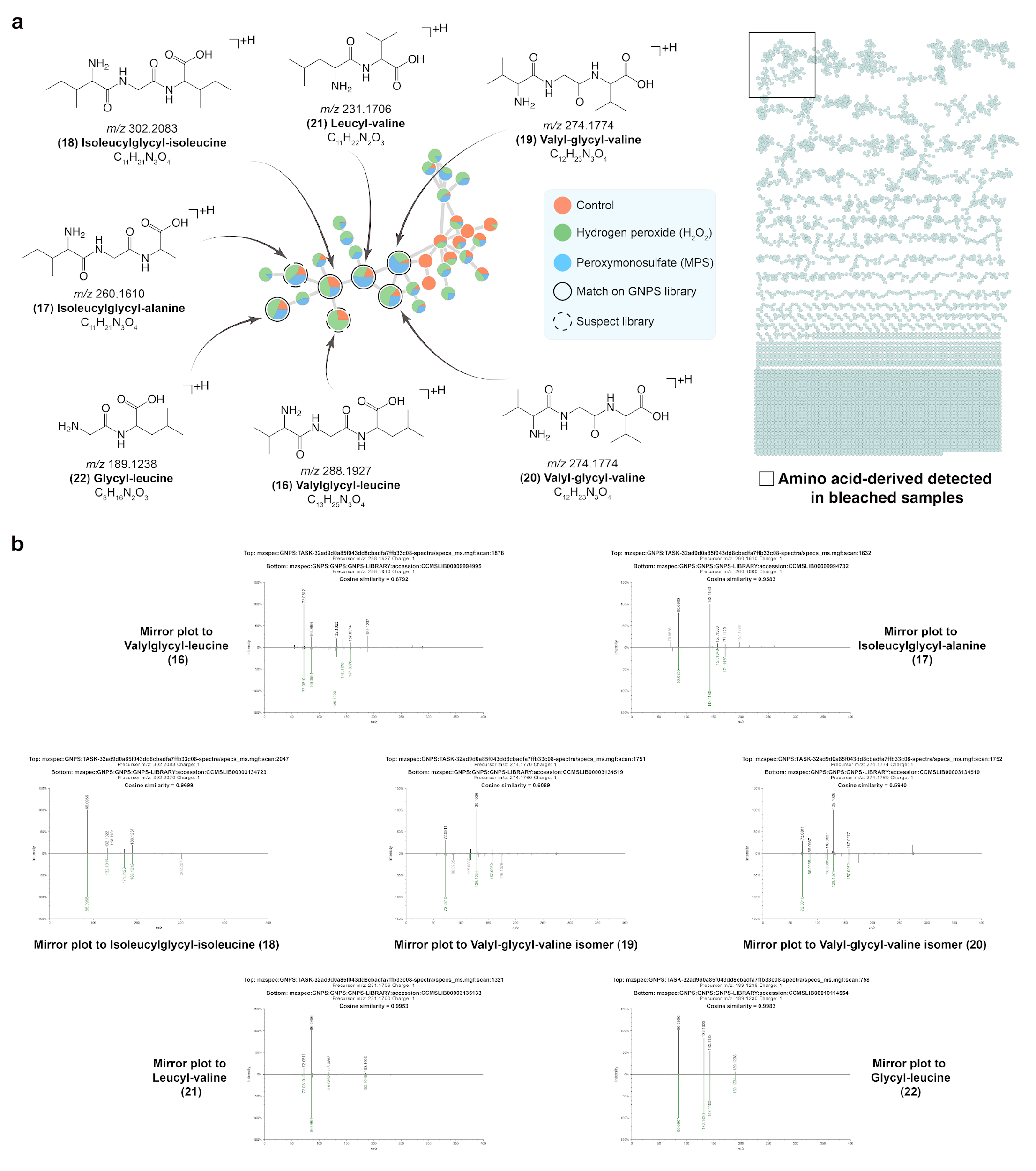
**

**Supplementary Figure 4. Molecular family of consistent features of amino acid-derived detected in H_2_O_2_/MPS.** a) Annotated amino acid-derived in both bleached samples; b) Each annotated metabolite has a mirror plot comparing a library MS/MS spectrum (top) and the teeth metabolite MS/MS spectrum (bottom) based on cosine similarity. Proposed chemical names and structures were listed when available.

**Supplementary Table 1. Effect size based on** **Cohen’s d estimate values.** a) Cohen’s d estimate for Control vs. H_2_O_2_. b) Cohen’s d estimate Control vs. MPS. c) Cohen’s d estimate for H_2_O_2_ vs. MPS.

| **Group** | **Cohen’s d estimate** | **Classification** |
| --- | --- | --- |
| Control vs. H_2_O_2_ | -2.312481 | Large |
| Control vs. MPS | -2.034352 | Large |
| H_2_O_2_ vs. MPS | 0.169950 | Negligible |

**Supplementary Table 2. Features with variable importance projection (VIP).** A total of 3,315 mass spectrometry features presented a VIP > 1.0.

| **Cluster ID_*m/z*** | **VIP score** | **Group Contribution** |
| --- | --- | --- |
| 783_191.1394 | 1.596358074 | H2O2 |
| 1706_270.1453 | 1.580194292 | H2O2 |
| 307_146.0815 | 1.564200211 | H2O2 |
| 239_138.0554 | 1.562742856 | H2O2 |
| 93_114.0552 | 1.560911963 | H2O2 |
| 174_128.0709 | 1.550045018 | H2O2 |
| 1550_253.109 | 1.545672255 | H2O2 |
| 420_158.0814 | 1.540334078 | H2O2 |
| 1304_230.15 | 1.526412118 | H2O2 |
| 1560_254.1075 | 1.518461408 | H2O2 |
| 1173_222.124 | 1.515629801 | H2O2 |
| 150_124.0875 | 1.495753293 | H2O2 |
| 747_188.1074 | 1.490408312 | H2O2 |
| 1677_266.1138 | 1.488397574 | H2O2 |
| 572_173.0925 | 1.480262168 | H2O2 |
| 847_196.1086 | 1.479316712 | H2O2 |
| 1585_256.1658 | 1.478215904 | H2O2 |
| 1140_218.1865 | 1.472535622 | H2O2 |
| 171_127.0869 | 1.469527399 | H2O2 |
| 461_162.0915 | 1.469242353 | H2O2 |
| 1557_253.18 | 1.468505435 | Control |
| 37_102.0915 | 1.462960046 | H2O2 |
| 815_194.093 | 1.462513437 | H2O2 |
| 733_187.0871 | 1.458972058 | H2O2 |
| 1454_244.1546 | 1.458904362 | Control |
| 2185_314.2691 | 1.458241064 | Control |
| 83_112.0759 | 1.456489654 | H2O2 |
| 1731_272.1858 | 1.45348127 | Control |
| 1982_297.2427 | 1.451315002 | Control |
| 1195_224.1031 | 1.450491944 | H2O2 |
| 124_122.0603 | 1.44875943 | H2O2 |
| 184_129.1025 | 1.447786838 | H2O2 |
| 1577_255.1957 | 1.445298229 | Control |
| 805_193.1341 | 1.442360094 | H2O2 |
| 465_162.1028 | 1.441007079 | H2O2 |
| 1734_272.2222 | 1.438114599 | Control |
| 960_206.1181 | 1.435991586 | H2O2 |
| 2117_308.2797 | 1.430855565 | Control |
| 383_154.1229 | 1.429521364 | H2O2 |
| 878_199.1082 | 1.429137425 | H2O2 |
| 1078_214.1189 | 1.428830561 | H2O2 |
| 250_139.087 | 1.427441256 | H2O2 |
| 581_173.1288 | 1.426668333 | H2O2 |
| 909_201.1028 | 1.425965451 | H2O2 |
| 483_164.0708 | 1.423265673 | H2O2 |
| 2346_329.0053 | 1.423249848 | Control |
| 768_190.123 | 1.422147953 | H2O2 |
| 316_148.0761 | 1.420740488 | H2O2 |
| 755_189.1027 | 1.420123209 | H2O2 |
| 2455_336.2023 | 1.418418605 | H2O2 |
| 1105_216.1343 | 1.417712157 | H2O2 |
| 530_168.1385 | 1.415402009 | H2O2 |
| 3222_424.3636 | 1.415209582 | Control |
| 488_164.1185 | 1.41347219 | H2O2 |
| 3026_394.353 | 1.41341485 | Control |
| 2268_322.2954 | 1.412682492 | Control |
| 1763_275.2081 | 1.409786763 | H2O2 |
| 492_165.0662 | 1.409010391 | H2O2 |
| 314_148.076 | 1.408815971 | H2O2 |
| 1458_244.1909 | 1.407392687 | Control |
| 3460_468.3898 | 1.407269498 | Control |
| 1011_209.154 | 1.406695781 | Control |
| 3517_478.3012 | 1.405273106 | H2O2 |
| 245_139.0757 | 1.404441513 | H2O2 |
| 1584_256.1547 | 1.404070788 | H2O2 |
| 1684_267.1722 | 1.403789982 | Control |
| 486_164.1072 | 1.403369915 | H2O2 |
| 1753_274.2015 | 1.402250096 | Control |
| 2626_350.3267 | 1.401581589 | Control |
| 546_170.1179 | 1.400755781 | H2O2 |
| 3366_452.2494 | 1.400537948 | Control |
| 1689_268.1293 | 1.399842765 | H2O2 |
| 1854_286.2742 | 1.398660761 | Control |
| 2293_325.1876 | 1.397625731 | H2O2 |
| 395_155.1181 | 1.397345148 | H2O2 |
| 1842_286.1403 | 1.396822018 | H2O2 |
| 745_188.0711 | 1.395456704 | H2O2 |
| 2241_318.3005 | 1.39474379 | Control |
| 1145_219.1132 | 1.39426465 | H2O2 |
| 2461_336.311 | 1.392767335 | Control |
| 2414_333.3001 | 1.390905133 | Control |
| 347_151.0966 | 1.388903229 | Control |
| 697_183.1134 | 1.388818611 | H2O2 |
| 2775_364.3424 | 1.387994583 | Control |
| 3353_449.3489 | 1.387925515 | H2O2 |
| 603_176.1073 | 1.38690296 | H2O2 |
| 2917_380.3374 | 1.385789151 | Control |
| 1090_215.1181 | 1.385785767 | H2O2 |
| 1702_269.2477 | 1.384510805 | Control |
| 426_158.1541 | 1.384060952 | Control |
| 1223_226.1186 | 1.382930793 | H2O2 |
| 1128_218.118 | 1.382433962 | H2O2 |
| 1315_231.1229 | 1.381951641 | H2O2 |
| 3299_438.3792 | 1.38184624 | Control |
| 919_202.098 | 1.380455538 | H2O2 |
| 911_201.1238 | 1.378524221 | H2O2 |
| 2390_331.2482 | 1.377890677 | Control |
| 1756_274.2742 | 1.377240401 | Control |
| 578_173.1288 | 1.376920164 | H2O2 |
| 513_166.1229 | 1.375525625 | H2O2 |
| 198_131.0706 | 1.374710788 | H2O2 |
| 1488_246.2429 | 1.373986444 | Control |
| 3678_503.4574 | 1.37300384 | H2O2 |
| 335_150.0916 | 1.372435157 | H2O2 |
| 2132_310.1881 | 1.371979457 | H2O2 |
| 107_115.0868 | 1.371162903 | H2O2 |
| 627_178.123 | 1.368703056 | H2O2 |
| 953_205.1339 | 1.36652691 | H2O2 |
| 6_88.0759 | 1.366423714 | Control |
| 14_90.526 | 1.366423714 | Control |
| 22_97.065 | 1.366423714 | Control |
| 116_118.0865 | 1.366423714 | Control |
| 117_118.0866 | 1.366423714 | Control |
| 134_123.0806 | 1.366423714 | Control |
| 135_123.0807 | 1.366423714 | Control |
| 137_123.0807 | 1.366423714 | Control |
| 140_123.0808 | 1.366423714 | Control |
| 141_123.0808 | 1.366423714 | Control |
| 160_125.9866 | 1.366423714 | Control |
| 200_132.056 | 1.366423714 | Control |
| 267_141.0548 | 1.366423714 | Control |
| 270_141.0915 | 1.366423714 | Control |
| 280_141.959 | 1.366423714 | Control |
| 325_149.0601 | 1.366423714 | Control |
| 363_153.0912 | 1.366423714 | Control |
| 386_155.0705 | 1.366423714 | Control |
| 387_155.0705 | 1.366423714 | Control |
| 413_157.0862 | 1.366423714 | Control |
| 453_160.0971 | 1.366423714 | Control |
| 454_161.0923 | 1.366423714 | Control |
| 493_165.0912 | 1.366423714 | Control |
| 590_174.1241 | 1.366423714 | Control |
| 662_181.1226 | 1.366423714 | Control |
| 681_182.9857 | 1.366423714 | Control |
| 692_183.1021 | 1.366423714 | Control |
| 693_183.1021 | 1.366423714 | Control |
| 778_191.0821 | 1.366423714 | Control |
| 798_193.0862 | 1.366423714 | Control |
| 799_193.0863 | 1.366423714 | Control |
| 820_194.1182 | 1.366423714 | Control |
| 857_197.0813 | 1.366423714 | Control |
| 865_197.1289 | 1.366423714 | Control |
| 959_206.1179 | 1.366423714 | Control |
| 1003_209.1287 | 1.366423714 | Control |
| 1019_210.1128 | 1.366423714 | Control |
| 1020_210.1128 | 1.366423714 | Control |
| 1028_211.0791 | 1.366423714 | Control |
| 1063_213.1126 | 1.366423714 | Control |
| 1077_214.1077 | 1.366423714 | Control |
| 1086_215.0704 | 1.366423714 | Control |
| 1087_215.0918 | 1.366423714 | Control |
| 1088_215.0918 | 1.366423714 | Control |
| 1121_217.1226 | 1.366423714 | Control |
| 1147_219.1377 | 1.366423714 | Control |
| 1206_225.1124 | 1.366423714 | Control |
| 1216_225.1488 | 1.366423714 | Control |
| 1235_226.1802 | 1.366423714 | Control |
| 1240_227.1029 | 1.366423714 | Control |
| 1241_227.1031 | 1.366423714 | Control |
| 1247_227.1281 | 1.366423714 | Control |
| 1319_231.1593 | 1.366423714 | Control |
| 1325_232.1179 | 1.366423714 | Control |
| 1326_232.1182 | 1.366423714 | Control |
| 1351_234.1492 | 1.366423714 | Control |
| 1352_234.1492 | 1.366423714 | Control |
| 1364_236.1285 | 1.366423714 | Control |
| 1421_241.1913 | 1.366423714 | Control |
| 1435_242.2844 | 1.366423714 | Control |
| 1441_243.1229 | 1.366423714 | Control |
| 1467_245.0956 | 1.366423714 | Control |
| 1468_245.1174 | 1.366423714 | Control |
| 1473_245.175 | 1.366423714 | Control |
| 1486_246.1703 | 1.366423714 | Control |
| 1503_248.1286 | 1.366423714 | Control |
| 1511_248.1648 | 1.366423714 | Control |
| 1516_249.1123 | 1.366423714 | Control |
| 1517_249.1123 | 1.366423714 | Control |
| 1518_249.1346 | 1.366423714 | Control |
| 1522_249.1486 | 1.366423714 | Control |
| 1528_250.1441 | 1.366423714 | Control |
| 1534_251.1278 | 1.366423714 | Control |
| 1535_251.128 | 1.366423714 | Control |
| 1596_256.3001 | 1.366423714 | Control |
| 1597_257.1173 | 1.366423714 | Control |
| 1624_260.1283 | 1.366423714 | Control |
| 1625_260.1285 | 1.366423714 | Control |
| 1642_261.1236 | 1.366423714 | Control |
| 1643_261.1487 | 1.366423714 | Control |
| 1644_261.1488 | 1.366423714 | Control |
| 1648_262.1439 | 1.366423714 | Control |
| 1651_262.1653 | 1.366423714 | Control |
| 1661_263.1643 | 1.366423714 | Control |
| 1669_265.107 | 1.366423714 | Control |
| 1670_265.1071 | 1.366423714 | Control |
| 1675_265.1437 | 1.366423714 | Control |
| 1688_268.1004 | 1.366423714 | Control |
| 1693_269.138 | 1.366423714 | Control |
| 1703_270.1127 | 1.366423714 | Control |
| 1710_270.1701 | 1.366423714 | Control |
| 1716_271.1172 | 1.366423714 | Control |
| 1759_275.1643 | 1.366423714 | Control |
| 1760_275.1644 | 1.366423714 | Control |
| 1761_275.1644 | 1.366423714 | Control |
| 1770_276.1811 | 1.366423714 | Control |
| 1777_277.1436 | 1.366423714 | Control |
| 1778_277.1437 | 1.366423714 | Control |
| 1779_277.1438 | 1.366423714 | Control |
| 1780_277.1802 | 1.366423714 | Control |
| 1799_280.0948 | 1.366423714 | Control |
| 1805_281.1384 | 1.366423714 | Control |
| 1818_283.1542 | 1.366423714 | Control |
| 1830_284.3313 | 1.366423714 | Control |
| 1832_285.1125 | 1.366423714 | Control |
| 1836_285.1891 | 1.366423714 | Control |
| 1853_286.2742 | 1.366423714 | Control |
| 1857_287.1278 | 1.366423714 | Control |
| 1858_287.1279 | 1.366423714 | Control |
| 1859_287.128 | 1.366423714 | Control |
| 1860_287.1281 | 1.366423714 | Control |
| 1863_287.1644 | 1.366423714 | Control |
| 1864_287.1646 | 1.366423714 | Control |
| 1865_287.1646 | 1.366423714 | Control |
| 1874_288.1808 | 1.366423714 | Control |
| 1876_288.1809 | 1.366423714 | Control |
| 1882_288.2543 | 1.366423714 | Control |
| 1886_289.1074 | 1.366423714 | Control |
| 1887_289.1435 | 1.366423714 | Control |
| 1888_289.1436 | 1.366423714 | Control |
| 1889_289.1436 | 1.366423714 | Control |
| 1890_289.1436 | 1.366423714 | Control |
| 1899_290.16 | 1.366423714 | Control |
| 1903_290.16 | 1.366423714 | Control |
| 1920_291.1227 | 1.366423714 | Control |
| 1921_291.1229 | 1.366423714 | Control |
| 1922_291.1597 | 1.366423714 | Control |
| 1923_291.1606 | 1.366423714 | Control |
| 1929_292.1763 | 1.366423714 | Control |
| 1936_293.1384 | 1.366423714 | Control |
| 1937_293.1384 | 1.366423714 | Control |
| 1938_293.1384 | 1.366423714 | Control |
| 1939_293.1386 | 1.366423714 | Control |
| 1940_293.1494 | 1.366423714 | Control |
| 1944_293.1867 | 1.366423714 | Control |
| 1948_294.1916 | 1.366423714 | Control |
| 1949_294.1916 | 1.366423714 | Control |
| 1950_294.1917 | 1.366423714 | Control |
| 1957_295.1165 | 1.366423714 | Control |
| 1960_295.1535 | 1.366423714 | Control |
| 1964_296.1285 | 1.366423714 | Control |
| 1966_296.1857 | 1.366423714 | Control |
| 1968_297.1158 | 1.366423714 | Control |
| 1970_297.1488 | 1.366423714 | Control |
| 1973_297.1699 | 1.366423714 | Control |
| 1984_297.2543 | 1.366423714 | Control |
| 1993_299.128 | 1.366423714 | Control |
| 1994_299.1489 | 1.366423714 | Control |
| 2006_299.2118 | 1.366423714 | Control |
| 2014_300.1962 | 1.366423714 | Control |
| 2028_301.1802 | 1.366423714 | Control |
| 2029_301.2164 | 1.366423714 | Control |
| 2030_301.2164 | 1.366423714 | Control |
| 2031_301.2164 | 1.366423714 | Control |
| 2037_302.1446 | 1.366423714 | Control |
| 2051_302.2692 | 1.366423714 | Control |
| 2057_303.1593 | 1.366423714 | Control |
| 2058_303.1594 | 1.366423714 | Control |
| 2059_303.16 | 1.366423714 | Control |
| 2061_303.232 | 1.366423714 | Control |
| 2063_304.1468 | 1.366423714 | Control |
| 2064_304.1613 | 1.366423714 | Control |
| 2071_304.1759 | 1.366423714 | Control |
| 2077_305.1386 | 1.366423714 | Control |
| 2078_305.1386 | 1.366423714 | Control |
| 2079_305.1386 | 1.366423714 | Control |
| 2080_305.1752 | 1.366423714 | Control |
| 2081_305.1755 | 1.366423714 | Control |
| 2082_305.1756 | 1.366423714 | Control |
| 2095_306.1915 | 1.366423714 | Control |
| 2096_306.1915 | 1.366423714 | Control |
| 2098_306.1916 | 1.366423714 | Control |
| 2099_306.1916 | 1.366423714 | Control |
| 2108_307.1177 | 1.366423714 | Control |
| 2109_307.1543 | 1.366423714 | Control |
| 2115_308.186 | 1.366423714 | Control |
| 2120_309.1595 | 1.366423714 | Control |
| 2121_309.1697 | 1.366423714 | Control |
| 2124_309.189 | 1.366423714 | Control |
| 2128_310.1443 | 1.366423714 | Control |
| 2129_310.165 | 1.366423714 | Control |
| 2130_310.1805 | 1.366423714 | Control |
| 2133_310.2018 | 1.366423714 | Control |
| 2136_311.1313 | 1.366423714 | Control |
| 2139_311.1479 | 1.366423714 | Control |
| 2140_311.1482 | 1.366423714 | Control |
| 2142_311.1646 | 1.366423714 | Control |
| 2160_313.1437 | 1.366423714 | Control |
| 2162_313.147 | 1.366423714 | Control |
| 2163_313.18 | 1.366423714 | Control |
| 2164_313.18 | 1.366423714 | Control |
| 2165_313.18 | 1.366423714 | Control |
| 2174_314.1499 | 1.366423714 | Control |
| 2176_314.1832 | 1.366423714 | Control |
| 2177_314.1833 | 1.366423714 | Control |
| 2179_314.1873 | 1.366423714 | Control |
| 2180_314.1873 | 1.366423714 | Control |
| 2187_315.1225 | 1.366423714 | Control |
| 2190_315.123 | 1.366423714 | Control |
| 2194_315.1593 | 1.366423714 | Control |
| 2195_315.1594 | 1.366423714 | Control |
| 2196_315.1596 | 1.366423714 | Control |
| 2197_315.1597 | 1.366423714 | Control |
| 2199_315.1861 | 1.366423714 | Control |
| 2202_316.1262 | 1.366423714 | Control |
| 2211_316.2276 | 1.366423714 | Control |
| 2217_317.175 | 1.366423714 | Control |
| 2218_317.1751 | 1.366423714 | Control |
| 2222_317.2114 | 1.366423714 | Control |
| 2223_318.1777 | 1.366423714 | Control |
| 2224_318.1781 | 1.366423714 | Control |
| 2232_318.1915 | 1.366423714 | Control |
| 2233_318.1916 | 1.366423714 | Control |
| 2234_318.1916 | 1.366423714 | Control |
| 2235_318.1916 | 1.366423714 | Control |
| 2236_318.1917 | 1.366423714 | Control |
| 2237_318.1922 | 1.366423714 | Control |
| 2242_319.1543 | 1.366423714 | Control |
| 2243_319.1543 | 1.366423714 | Control |
| 2244_319.1544 | 1.366423714 | Control |
| 2245_319.1545 | 1.366423714 | Control |
| 2247_319.227 | 1.366423714 | Control |
| 2260_321.17 | 1.366423714 | Control |
| 2261_321.17 | 1.366423714 | Control |
| 2262_321.2403 | 1.366423714 | Control |
| 2265_322.1864 | 1.366423714 | Control |
| 2269_323.1281 | 1.366423714 | Control |
| 2272_323.1751 | 1.366423714 | Control |
| 2279_324.1809 | 1.366423714 | Control |
| 2280_324.1809 | 1.366423714 | Control |
| 2287_325.1416 | 1.366423714 | Control |
| 2289_325.1438 | 1.366423714 | Control |
| 2290_325.1643 | 1.366423714 | Control |
| 2291_325.1643 | 1.366423714 | Control |
| 2304_326.1754 | 1.366423714 | Control |
| 2305_326.1755 | 1.366423714 | Control |
| 2306_326.1756 | 1.366423714 | Control |
| 2315_327.1431 | 1.366423714 | Control |
| 2316_327.1593 | 1.366423714 | Control |
| 2317_327.1594 | 1.366423714 | Control |
| 2318_327.1594 | 1.366423714 | Control |
| 2319_327.1595 | 1.366423714 | Control |
| 2325_328.1623 | 1.366423714 | Control |
| 2326_328.1626 | 1.366423714 | Control |
| 2327_328.1652 | 1.366423714 | Control |
| 2328_328.1759 | 1.366423714 | Control |
| 2329_328.1911 | 1.366423714 | Control |
| 2330_328.1911 | 1.366423714 | Control |
| 2331_328.1912 | 1.366423714 | Control |
| 2332_328.1912 | 1.366423714 | Control |
| 2333_328.1913 | 1.366423714 | Control |
| 2334_328.1914 | 1.366423714 | Control |
| 2335_328.1914 | 1.366423714 | Control |
| 2339_328.233 | 1.366423714 | Control |
| 2350_329.1655 | 1.366423714 | Control |
| 2351_329.175 | 1.366423714 | Control |
| 2352_329.1751 | 1.366423714 | Control |
| 2353_329.1751 | 1.366423714 | Control |
| 2354_329.1752 | 1.366423714 | Control |
| 2355_329.1752 | 1.366423714 | Control |
| 2358_329.194 | 1.366423714 | Control |
| 2359_329.1943 | 1.366423714 | Control |
| 2366_330.1677 | 1.366423714 | Control |
| 2367_330.1703 | 1.366423714 | Control |
| 2368_330.178 | 1.366423714 | Control |
| 2369_330.1783 | 1.366423714 | Control |
| 2370_330.1821 | 1.366423714 | Control |
| 2380_331.1544 | 1.366423714 | Control |
| 2381_331.1544 | 1.366423714 | Control |
| 2382_331.1544 | 1.366423714 | Control |
| 2383_331.1545 | 1.366423714 | Control |
| 2385_331.1909 | 1.366423714 | Control |
| 2386_331.1913 | 1.366423714 | Control |
| 2402_332.2071 | 1.366423714 | Control |
| 2406_333.1314 | 1.366423714 | Control |
| 2407_333.1464 | 1.366423714 | Control |
| 2408_333.1699 | 1.366423714 | Control |
| 2409_333.1703 | 1.366423714 | Control |
| 2410_333.1704 | 1.366423714 | Control |
| 2412_333.2072 | 1.366423714 | Control |
| 2420_334.202 | 1.366423714 | Control |
| 2428_334.2954 | 1.366423714 | Control |
| 2429_335.1486 | 1.366423714 | Control |
| 2430_335.1491 | 1.366423714 | Control |
| 2431_335.162 | 1.366423714 | Control |
| 2432_335.1621 | 1.366423714 | Control |
| 2433_335.1622 | 1.366423714 | Control |
| 2434_335.1623 | 1.366423714 | Control |
| 2446_336.181 | 1.366423714 | Control |
| 2448_336.2021 | 1.366423714 | Control |
| 2452_336.2023 | 1.366423714 | Control |
| 2454_336.2023 | 1.366423714 | Control |
| 2456_336.2178 | 1.366423714 | Control |
| 2460_336.2539 | 1.366423714 | Control |
| 2466_337.3147 | 1.366423714 | Control |
| 2468_338.1601 | 1.366423714 | Control |
| 2469_338.1966 | 1.366423714 | Control |
| 2470_338.2122 | 1.366423714 | Control |
| 2471_338.2123 | 1.366423714 | Control |
| 2479_339.1805 | 1.366423714 | Control |
| 2480_339.1937 | 1.366423714 | Control |
| 2488_339.3014 | 1.366423714 | Control |
| 2490_340.1548 | 1.366423714 | Control |
| 2491_340.1758 | 1.366423714 | Control |
| 2492_341.1384 | 1.366423714 | Control |
| 2493_341.1386 | 1.366423714 | Control |
| 2494_341.1386 | 1.366423714 | Control |
| 2496_341.1753 | 1.366423714 | Control |
| 2497_341.1754 | 1.366423714 | Control |
| 2501_342.1706 | 1.366423714 | Control |
| 2502_342.171 | 1.366423714 | Control |
| 2503_342.1711 | 1.366423714 | Control |
| 2512_343.1364 | 1.366423714 | Control |
| 2513_343.1534 | 1.366423714 | Control |
| 2514_343.1539 | 1.366423714 | Control |
| 2518_343.1544 | 1.366423714 | Control |
| 2520_343.1544 | 1.366423714 | Control |
| 2521_343.1544 | 1.366423714 | Control |
| 2522_343.1748 | 1.366423714 | Control |
| 2524_343.1908 | 1.366423714 | Control |
| 2525_343.1908 | 1.366423714 | Control |
| 2526_343.1908 | 1.366423714 | Control |
| 2532_344.1859 | 1.366423714 | Control |
| 2533_344.1861 | 1.366423714 | Control |
| 2534_344.1861 | 1.366423714 | Control |
| 2536_344.1861 | 1.366423714 | Control |
| 2550_344.3526 | 1.366423714 | Control |
| 2551_345.1336 | 1.366423714 | Control |
| 2553_345.1701 | 1.366423714 | Control |
| 2554_345.1702 | 1.366423714 | Control |
| 2555_345.1702 | 1.366423714 | Control |
| 2556_345.1703 | 1.366423714 | Control |
| 2557_345.1703 | 1.366423714 | Control |
| 2567_346.2018 | 1.366423714 | Control |
| 2576_346.223 | 1.366423714 | Control |
| 2580_347.1493 | 1.366423714 | Control |
| 2583_347.1858 | 1.366423714 | Control |
| 2584_347.186 | 1.366423714 | Control |
| 2593_348.181 | 1.366423714 | Control |
| 2602_348.2177 | 1.366423714 | Control |
| 2603_348.2179 | 1.366423714 | Control |
| 2608_349.1412 | 1.366423714 | Control |
| 2609_349.1648 | 1.366423714 | Control |
| 2610_349.165 | 1.366423714 | Control |
| 2614_350.1727 | 1.366423714 | Control |
| 2615_350.1966 | 1.366423714 | Control |
| 2616_350.1965 | 1.366423714 | Control |
| 2617_350.1966 | 1.366423714 | Control |
| 2618_350.1967 | 1.366423714 | Control |
| 2623_350.2179 | 1.366423714 | Control |
| 2628_351.1555 | 1.366423714 | Control |
| 2629_351.157 | 1.366423714 | Control |
| 2630_351.1571 | 1.366423714 | Control |
| 2631_351.1573 | 1.366423714 | Control |
| 2632_351.1802 | 1.366423714 | Control |
| 2635_352.1758 | 1.366423714 | Control |
| 2637_352.2121 | 1.366423714 | Control |
| 2653_353.186 | 1.366423714 | Control |
| 2658_354.2068 | 1.366423714 | Control |
| 2663_355.1519 | 1.366423714 | Control |
| 2664_355.1522 | 1.366423714 | Control |
| 2666_355.2015 | 1.366423714 | Control |
| 2675_356.1705 | 1.366423714 | Control |
| 2676_356.1862 | 1.366423714 | Control |
| 2677_356.1862 | 1.366423714 | Control |
| 2678_356.1862 | 1.366423714 | Control |
| 2679_356.207 | 1.366423714 | Control |
| 2680_356.207 | 1.366423714 | Control |
| 2681_356.2225 | 1.366423714 | Control |
| 2688_357.1312 | 1.366423714 | Control |
| 2691_357.1701 | 1.366423714 | Control |
| 2696_358.2019 | 1.366423714 | Control |
| 2697_358.2021 | 1.366423714 | Control |
| 2701_358.2383 | 1.366423714 | Control |
| 2702_358.2386 | 1.366423714 | Control |
| 2705_358.3682 | 1.366423714 | Control |
| 2706_359.1314 | 1.366423714 | Control |
| 2707_359.1314 | 1.366423714 | Control |
| 2708_359.1479 | 1.366423714 | Control |
| 2709_359.1492 | 1.366423714 | Control |
| 2710_359.1858 | 1.366423714 | Control |
| 2711_359.1858 | 1.366423714 | Control |
| 2712_359.186 | 1.366423714 | Control |
| 2714_359.2057 | 1.366423714 | Control |
| 2716_360.1808 | 1.366423714 | Control |
| 2717_360.1809 | 1.366423714 | Control |
| 2722_360.1811 | 1.366423714 | Control |
| 2723_360.1813 | 1.366423714 | Control |
| 2724_360.2021 | 1.366423714 | Control |
| 2727_360.2173 | 1.366423714 | Control |
| 2730_361.1648 | 1.366423714 | Control |
| 2731_361.1648 | 1.366423714 | Control |
| 2732_361.1649 | 1.366423714 | Control |
| 2733_361.165 | 1.366423714 | Control |
| 2734_361.202 | 1.366423714 | Control |
| 2737_362.1964 | 1.366423714 | Control |
| 2738_362.1964 | 1.366423714 | Control |
| 2744_362.2177 | 1.366423714 | Control |
| 2745_362.2338 | 1.366423714 | Control |
| 2748_363.1805 | 1.366423714 | Control |
| 2749_363.1806 | 1.366423714 | Control |
| 2750_363.1806 | 1.366423714 | Control |
| 2751_363.1807 | 1.366423714 | Control |
| 2755_363.2371 | 1.366423714 | Control |
| 2758_364.1763 | 1.366423714 | Control |
| 2761_364.2121 | 1.366423714 | Control |
| 2762_364.2121 | 1.366423714 | Control |
| 2763_364.2123 | 1.366423714 | Control |
| 2764_364.2124 | 1.366423714 | Control |
| 2770_364.2335 | 1.366423714 | Control |
| 2776_365.1156 | 1.366423714 | Control |
| 2780_365.173 | 1.366423714 | Control |
| 2782_366.1915 | 1.366423714 | Control |
| 2783_366.1915 | 1.366423714 | Control |
| 2784_366.1917 | 1.366423714 | Control |
| 2789_366.2282 | 1.366423714 | Control |
| 2794_367.1312 | 1.366423714 | Control |
| 2795_367.1521 | 1.366423714 | Control |
| 2796_367.1522 | 1.366423714 | Control |
| 2799_367.1745 | 1.366423714 | Control |
| 2801_367.1746 | 1.366423714 | Control |
| 2806_368.2073 | 1.366423714 | Control |
| 2807_368.2436 | 1.366423714 | Control |
| 2808_369.1313 | 1.366423714 | Control |
| 2809_369.1676 | 1.366423714 | Control |
| 2810_369.1812 | 1.366423714 | Control |
| 2816_369.3841 | 1.366423714 | Control |
| 2817_370.1856 | 1.366423714 | Control |
| 2819_370.2022 | 1.366423714 | Control |
| 2827_371.147 | 1.366423714 | Control |
| 2828_371.1479 | 1.366423714 | Control |
| 2831_371.1856 | 1.366423714 | Control |
| 2836_372.202 | 1.366423714 | Control |
| 2837_372.2172 | 1.366423714 | Control |
| 2838_372.2191 | 1.366423714 | Control |
| 2842_373.1646 | 1.366423714 | Control |
| 2843_373.1648 | 1.366423714 | Control |
| 2844_373.1649 | 1.366423714 | Control |
| 2850_374.1975 | 1.366423714 | Control |
| 2854_374.2334 | 1.366423714 | Control |
| 2855_374.2336 | 1.366423714 | Control |
| 2856_374.2338 | 1.366423714 | Control |
| 2858_375.1627 | 1.366423714 | Control |
| 2859_375.1806 | 1.366423714 | Control |
| 2860_375.1806 | 1.366423714 | Control |
| 2861_375.1807 | 1.366423714 | Control |
| 2862_375.1808 | 1.366423714 | Control |
| 2864_376.2124 | 1.366423714 | Control |
| 2865_376.2124 | 1.366423714 | Control |
| 2866_376.2124 | 1.366423714 | Control |
| 2872_376.2335 | 1.366423714 | Control |
| 2874_376.2493 | 1.366423714 | Control |
| 2875_376.2503 | 1.366423714 | Control |
| 2878_377.1598 | 1.366423714 | Control |
| 2879_377.1964 | 1.366423714 | Control |
| 2884_378.1916 | 1.366423714 | Control |
| 2885_378.1919 | 1.366423714 | Control |
| 2891_378.2283 | 1.366423714 | Control |
| 2892_378.2288 | 1.366423714 | Control |
| 2897_379.175 | 1.366423714 | Control |
| 2898_379.1755 | 1.366423714 | Control |
| 2899_379.1755 | 1.366423714 | Control |
| 2900_379.1947 | 1.366423714 | Control |
| 2902_379.2313 | 1.366423714 | Control |
| 2904_380.1703 | 1.366423714 | Control |
| 2906_380.2068 | 1.366423714 | Control |
| 2907_380.207 | 1.366423714 | Control |
| 2908_380.2071 | 1.366423714 | Control |
| 2913_380.2285 | 1.366423714 | Control |
| 2914_380.2799 | 1.366423714 | Control |
| 2919_381.1676 | 1.366423714 | Control |
| 2921_382.1862 | 1.366423714 | Control |
| 2926_383.1468 | 1.366423714 | Control |
| 2934_384.2019 | 1.366423714 | Control |
| 2935_384.2019 | 1.366423714 | Control |
| 2936_384.2022 | 1.366423714 | Control |
| 2937_384.2023 | 1.366423714 | Control |
| 2942_385.1624 | 1.366423714 | Control |
| 2943_385.1625 | 1.366423714 | Control |
| 2953_385.379 | 1.366423714 | Control |
| 2954_386.1786 | 1.366423714 | Control |
| 2956_386.2702 | 1.366423714 | Control |
| 2963_388.2123 | 1.366423714 | Control |
| 2967_388.2491 | 1.366423714 | Control |
| 2973_390.1913 | 1.366423714 | Control |
| 2975_390.2278 | 1.366423714 | Control |
| 2976_390.2279 | 1.366423714 | Control |
| 2977_390.228 | 1.366423714 | Control |
| 2978_390.2281 | 1.366423714 | Control |
| 2979_390.2282 | 1.366423714 | Control |
| 2985_391.1755 | 1.366423714 | Control |
| 2989_392.1836 | 1.366423714 | Control |
| 2991_392.2069 | 1.366423714 | Control |
| 2995_392.2284 | 1.366423714 | Control |
| 2999_392.2446 | 1.366423714 | Control |
| 3000_392.2448 | 1.366423714 | Control |
| 3001_392.2452 | 1.366423714 | Control |
| 3010_393.2108 | 1.366423714 | Control |
| 3015_394.1862 | 1.366423714 | Control |
| 3018_394.2227 | 1.366423714 | Control |
| 3019_394.2228 | 1.366423714 | Control |
| 3020_394.2229 | 1.366423714 | Control |
| 3021_394.223 | 1.366423714 | Control |
| 3027_395.1856 | 1.366423714 | Control |
| 3029_396.1268 | 1.366423714 | Control |
| 3030_396.2007 | 1.366423714 | Control |
| 3031_396.2018 | 1.366423714 | Control |
| 3032_396.2177 | 1.366423714 | Control |
| 3035_396.2536 | 1.366423714 | Control |
| 3037_397.1626 | 1.366423714 | Control |
| 3039_397.2018 | 1.366423714 | Control |
| 3040_398.1787 | 1.366423714 | Control |
| 3041_398.2174 | 1.366423714 | Control |
| 3042_398.2172 | 1.366423714 | Control |
| 3050_399.2172 | 1.366423714 | Control |
| 3055_400.1581 | 1.366423714 | Control |
| 3058_400.2124 | 1.366423714 | Control |
| 3060_400.2486 | 1.366423714 | Control |
| 3067_401.1403 | 1.366423714 | Control |
| 3068_401.1573 | 1.366423714 | Control |
| 3073_402.2275 | 1.366423714 | Control |
| 3075_402.2644 | 1.366423714 | Control |
| 3080_403.1752 | 1.366423714 | Control |
| 3081_403.1752 | 1.366423714 | Control |
| 3082_403.2125 | 1.366423714 | Control |
| 3085_404.1893 | 1.366423714 | Control |
| 3088_404.2438 | 1.366423714 | Control |
| 3089_404.244 | 1.366423714 | Control |
| 3096_406.2225 | 1.366423714 | Control |
| 3097_406.2225 | 1.366423714 | Control |
| 3106_407.147 | 1.366423714 | Control |
| 3107_407.1524 | 1.366423714 | Control |
| 3108_407.1704 | 1.366423714 | Control |
| 3109_407.2184 | 1.366423714 | Control |
| 3110_407.2259 | 1.366423714 | Control |
| 3113_408.181 | 1.366423714 | Control |
| 3114_408.2012 | 1.366423714 | Control |
| 3115_408.2179 | 1.366423714 | Control |
| 3120_408.2384 | 1.366423714 | Control |
| 3121_408.2384 | 1.366423714 | Control |
| 3135_409.2013 | 1.366423714 | Control |
| 3137_410.1963 | 1.366423714 | Control |
| 3138_410.2171 | 1.366423714 | Control |
| 3139_410.2344 | 1.366423714 | Control |
| 3149_411.2176 | 1.366423714 | Control |
| 3150_411.2282 | 1.366423714 | Control |
| 3154_412.179 | 1.366423714 | Control |
| 3155_412.2122 | 1.366423714 | Control |
| 3160_413.2327 | 1.366423714 | Control |
| 3164_414.1739 | 1.366423714 | Control |
| 3170_415.1909 | 1.366423714 | Control |
| 3180_416.2436 | 1.366423714 | Control |
| 3188_419.2068 | 1.366423714 | Control |
| 3189_420.2376 | 1.366423714 | Control |
| 3190_420.2377 | 1.366423714 | Control |
| 3191_420.2381 | 1.366423714 | Control |
| 3198_421.2009 | 1.366423714 | Control |
| 3203_422.217 | 1.366423714 | Control |
| 3210_423.1803 | 1.366423714 | Control |
| 3211_423.217 | 1.366423714 | Control |
| 3212_423.2176 | 1.366423714 | Control |
| 3214_423.2277 | 1.366423714 | Control |
| 3217_424.2123 | 1.366423714 | Control |
| 3218_424.2328 | 1.366423714 | Control |
| 3225_425.1951 | 1.366423714 | Control |
| 3226_425.1958 | 1.366423714 | Control |
| 3227_425.1959 | 1.366423714 | Control |
| 3230_425.2326 | 1.366423714 | Control |
| 3236_426.2279 | 1.366423714 | Control |
| 3237_426.2649 | 1.366423714 | Control |
| 3240_427.1748 | 1.366423714 | Control |
| 3241_427.2114 | 1.366423714 | Control |
| 3242_427.2117 | 1.366423714 | Control |
| 3243_427.2118 | 1.366423714 | Control |
| 3248_429.2275 | 1.366423714 | Control |
| 3249_430.2219 | 1.366423714 | Control |
| 3250_430.2224 | 1.366423714 | Control |
| 3259_432.2376 | 1.366423714 | Control |
| 3260_432.2378 | 1.366423714 | Control |
| 3265_433.2012 | 1.366423714 | Control |
| 3277_435.2493 | 1.366423714 | Control |
| 3278_435.2532 | 1.366423714 | Control |
| 3284_436.2717 | 1.366423714 | Control |
| 3285_436.2852 | 1.366423714 | Control |
| 3290_437.374 | 1.366423714 | Control |
| 3291_438.2122 | 1.366423714 | Control |
| 3292_438.2483 | 1.366423714 | Control |
| 3303_439.2485 | 1.366423714 | Control |
| 3304_439.2485 | 1.366423714 | Control |
| 3305_439.2492 | 1.366423714 | Control |
| 3306_439.2487 | 1.366423714 | Control |
| 3307_439.2488 | 1.366423714 | Control |
| 3309_439.2771 | 1.366423714 | Control |
| 3310_440.2102 | 1.366423714 | Control |
| 3315_441.2276 | 1.366423714 | Control |
| 3316_441.2281 | 1.366423714 | Control |
| 3319_442.2227 | 1.366423714 | Control |
| 3320_442.2229 | 1.366423714 | Control |
| 3325_443.2193 | 1.366423714 | Control |
| 3327_444.2378 | 1.366423714 | Control |
| 3328_444.2381 | 1.366423714 | Control |
| 3329_444.2388 | 1.366423714 | Control |
| 3330_444.2747 | 1.366423714 | Control |
| 3334_446.1984 | 1.366423714 | Control |
| 3339_448.2325 | 1.366423714 | Control |
| 3340_448.2329 | 1.366423714 | Control |
| 3343_448.2855 | 1.366423714 | Control |
| 3348_449.1934 | 1.366423714 | Control |
| 3349_449.1958 | 1.366423714 | Control |
| 3350_449.196 | 1.366423714 | Control |
| 3352_449.2542 | 1.366423714 | Control |
| 3355_450.2337 | 1.366423714 | Control |
| 3361_451.212 | 1.366423714 | Control |
| 3362_451.2478 | 1.366423714 | Control |
| 3363_451.2482 | 1.366423714 | Control |
| 3364_451.2489 | 1.366423714 | Control |
| 3365_452.2446 | 1.366423714 | Control |
| 3367_452.2653 | 1.366423714 | Control |
| 3370_452.395 | 1.366423714 | Control |
| 3371_453.1889 | 1.366423714 | Control |
| 3372_453.2275 | 1.366423714 | Control |
| 3373_453.2276 | 1.366423714 | Control |
| 3374_453.2277 | 1.366423714 | Control |
| 3377_454.2596 | 1.366423714 | Control |
| 3383_455.2063 | 1.366423714 | Control |
| 3384_455.2066 | 1.366423714 | Control |
| 3385_455.2431 | 1.366423714 | Control |
| 3386_455.2434 | 1.366423714 | Control |
| 3387_455.2435 | 1.366423714 | Control |
| 3388_455.2435 | 1.366423714 | Control |
| 3392_456.2379 | 1.366423714 | Control |
| 3399_458.2534 | 1.366423714 | Control |
| 3400_458.2538 | 1.366423714 | Control |
| 3406_459.2531 | 1.366423714 | Control |
| 3407_460.2232 | 1.366423714 | Control |
| 3408_460.2686 | 1.366423714 | Control |
| 3409_460.2685 | 1.366423714 | Control |
| 3413_461.269 | 1.366423714 | Control |
| 3414_461.2691 | 1.366423714 | Control |
| 3415_461.2691 | 1.366423714 | Control |
| 3416_461.2692 | 1.366423714 | Control |
| 3417_461.2725 | 1.366423714 | Control |
| 3418_461.2911 | 1.366423714 | Control |
| 3421_462.2484 | 1.366423714 | Control |
| 3423_462.2748 | 1.366423714 | Control |
| 3428_463.2112 | 1.366423714 | Control |
| 3429_463.2117 | 1.366423714 | Control |
| 3435_464.2629 | 1.366423714 | Control |
| 3438_465.2268 | 1.366423714 | Control |
| 3439_465.227 | 1.366423714 | Control |
| 3440_465.2633 | 1.366423714 | Control |
| 3441_465.2634 | 1.366423714 | Control |
| 3443_466.2802 | 1.366423714 | Control |
| 3451_467.2427 | 1.366423714 | Control |
| 3452_467.2429 | 1.366423714 | Control |
| 3453_467.243 | 1.366423714 | Control |
| 3454_467.243 | 1.366423714 | Control |
| 3455_467.2431 | 1.366423714 | Control |
| 3457_468.2749 | 1.366423714 | Control |
| 3461_468.3901 | 1.366423714 | Control |
| 3462_469.2222 | 1.366423714 | Control |
| 3464_469.2583 | 1.366423714 | Control |
| 3465_469.2587 | 1.366423714 | Control |
| 3466_469.2587 | 1.366423714 | Control |
| 3471_470.2541 | 1.366423714 | Control |
| 3472_470.2542 | 1.366423714 | Control |
| 3473_470.2543 | 1.366423714 | Control |
| 3474_470.2546 | 1.366423714 | Control |
| 3475_470.2548 | 1.366423714 | Control |
| 3476_470.2556 | 1.366423714 | Control |
| 3477_470.2611 | 1.366423714 | Control |
| 3478_470.2905 | 1.366423714 | Control |
| 3481_471.238 | 1.366423714 | Control |
| 3483_472.2692 | 1.366423714 | Control |
| 3484_472.2694 | 1.366423714 | Control |
| 3485_472.2696 | 1.366423714 | Control |
| 3486_472.2698 | 1.366423714 | Control |
| 3488_473.2172 | 1.366423714 | Control |
| 3489_473.2324 | 1.366423714 | Control |
| 3490_473.2325 | 1.366423714 | Control |
| 3491_473.2327 | 1.366423714 | Control |
| 3493_473.2726 | 1.366423714 | Control |
| 3494_474.2482 | 1.366423714 | Control |
| 3495_474.2484 | 1.366423714 | Control |
| 3496_474.2661 | 1.366423714 | Control |
| 3497_475.2475 | 1.366423714 | Control |
| 3498_475.2481 | 1.366423714 | Control |
| 3499_476.2176 | 1.366423714 | Control |
| 3500_476.2633 | 1.366423714 | Control |
| 3502_476.3005 | 1.366423714 | Control |
| 3504_477.1914 | 1.366423714 | Control |
| 3505_477.225 | 1.366423714 | Control |
| 3506_477.2269 | 1.366423714 | Control |
| 3507_477.2269 | 1.366423714 | Control |
| 3508_477.2274 | 1.366423714 | Control |
| 3509_477.2633 | 1.366423714 | Control |
| 3510_477.2633 | 1.366423714 | Control |
| 3511_477.2636 | 1.366423714 | Control |
| 3514_478.2582 | 1.366423714 | Control |
| 3515_478.2657 | 1.366423714 | Control |
| 3521_479.2066 | 1.366423714 | Control |
| 3522_479.2424 | 1.366423714 | Control |
| 3523_479.2425 | 1.366423714 | Control |
| 3524_479.2792 | 1.366423714 | Control |
| 3528_480.426 | 1.366423714 | Control |
| 3530_481.2035 | 1.366423714 | Control |
| 3531_481.2218 | 1.366423714 | Control |
| 3532_481.2219 | 1.366423714 | Control |
| 3533_481.2222 | 1.366423714 | Control |
| 3534_481.2584 | 1.366423714 | Control |
| 3535_481.2584 | 1.366423714 | Control |
| 3536_481.2586 | 1.366423714 | Control |
| 3537_482.291 | 1.366423714 | Control |
| 3538_482.2926 | 1.366423714 | Control |
| 3545_483.2372 | 1.366423714 | Control |
| 3546_483.2373 | 1.366423714 | Control |
| 3547_483.2375 | 1.366423714 | Control |
| 3548_483.2511 | 1.366423714 | Control |
| 3552_484.2343 | 1.366423714 | Control |
| 3555_484.27 | 1.366423714 | Control |
| 3556_484.2702 | 1.366423714 | Control |
| 3557_484.306 | 1.366423714 | Control |
| 3558_484.3056 | 1.366423714 | Control |
| 3559_484.3059 | 1.366423714 | Control |
| 3563_485.2535 | 1.366423714 | Control |
| 3564_485.2536 | 1.366423714 | Control |
| 3566_486.2486 | 1.366423714 | Control |
| 3567_486.2494 | 1.366423714 | Control |
| 3568_486.2505 | 1.366423714 | Control |
| 3571_486.2844 | 1.366423714 | Control |
| 3572_486.2848 | 1.366423714 | Control |
| 3573_486.2851 | 1.366423714 | Control |
| 3574_486.2853 | 1.366423714 | Control |
| 3575_486.2853 | 1.366423714 | Control |
| 3577_487.2328 | 1.366423714 | Control |
| 3580_488.2637 | 1.366423714 | Control |
| 3581_488.2642 | 1.366423714 | Control |
| 3582_488.2654 | 1.366423714 | Control |
| 3584_488.3003 | 1.366423714 | Control |
| 3586_489.2252 | 1.366423714 | Control |
| 3587_489.2269 | 1.366423714 | Control |
| 3588_489.2273 | 1.366423714 | Control |
| 3589_489.2274 | 1.366423714 | Control |
| 3590_489.2491 | 1.366423714 | Control |
| 3592_489.2972 | 1.366423714 | Control |
| 3593_489.3587 | 1.366423714 | Control |
| 3595_490.2435 | 1.366423714 | Control |
| 3596_490.2596 | 1.366423714 | Control |
| 3599_491.2428 | 1.366423714 | Control |
| 3600_491.2429 | 1.366423714 | Control |
| 3602_491.2431 | 1.366423714 | Control |
| 3606_492.2766 | 1.366423714 | Control |
| 3613_493.221 | 1.366423714 | Control |
| 3614_493.2223 | 1.366423714 | Control |
| 3615_493.2221 | 1.366423714 | Control |
| 3616_493.2224 | 1.366423714 | Control |
| 3618_493.2576 | 1.366423714 | Control |
| 3619_493.2578 | 1.366423714 | Control |
| 3620_493.2579 | 1.366423714 | Control |
| 3621_493.2582 | 1.366423714 | Control |
| 3622_493.2584 | 1.366423714 | Control |
| 3623_494.2525 | 1.366423714 | Control |
| 3624_494.2616 | 1.366423714 | Control |
| 3627_495.238 | 1.366423714 | Control |
| 3628_495.2378 | 1.366423714 | Control |
| 3629_495.2545 | 1.366423714 | Control |
| 3630_495.2743 | 1.366423714 | Control |
| 3631_496.2703 | 1.366423714 | Control |
| 3633_496.3059 | 1.366423714 | Control |
| 3638_497.2302 | 1.366423714 | Control |
| 3639_497.2307 | 1.366423714 | Control |
| 3640_497.2538 | 1.366423714 | Control |
| 3641_497.2539 | 1.366423714 | Control |
| 3645_498.2491 | 1.366423714 | Control |
| 3646_498.2853 | 1.366423714 | Control |
| 3647_498.2855 | 1.366423714 | Control |
| 3648_498.2855 | 1.366423714 | Control |
| 3649_498.2856 | 1.366423714 | Control |
| 3650_498.2858 | 1.366423714 | Control |
| 3653_499.2322 | 1.366423714 | Control |
| 3654_499.2467 | 1.366423714 | Control |
| 3655_499.2694 | 1.366423714 | Control |
| 3657_499.3511 | 1.366423714 | Control |
| 3659_500.2283 | 1.366423714 | Control |
| 3660_500.2645 | 1.366423714 | Control |
| 3661_500.265 | 1.366423714 | Control |
| 3662_500.3 | 1.366423714 | Control |
| 3663_500.3005 | 1.366423714 | Control |
| 3664_500.3007 | 1.366423714 | Control |
| 3665_500.3008 | 1.366423714 | Control |
| 3666_500.3008 | 1.366423714 | Control |
| 3669_501.3038 | 1.366423714 | Control |
| 3670_501.3042 | 1.366423714 | Control |
| 3672_502.2792 | 1.366423714 | Control |
| 3673_502.28 | 1.366423714 | Control |
| 3674_502.3477 | 1.366423714 | Control |
| 3679_504.259 | 1.366423714 | Control |
| 3680_504.2591 | 1.366423714 | Control |
| 3681_505.2224 | 1.366423714 | Control |
| 3682_505.2226 | 1.366423714 | Control |
| 3683_505.2226 | 1.366423714 | Control |
| 3685_505.2582 | 1.366423714 | Control |
| 3686_505.2583 | 1.366423714 | Control |
| 3687_505.3174 | 1.366423714 | Control |
| 3690_506.2918 | 1.366423714 | Control |
| 3691_506.3283 | 1.366423714 | Control |
| 3696_507.2366 | 1.366423714 | Control |
| 3697_507.2386 | 1.366423714 | Control |
| 3698_507.2387 | 1.366423714 | Control |
| 3699_507.2387 | 1.366423714 | Control |
| 3702_508.2714 | 1.366423714 | Control |
| 3703_508.2725 | 1.366423714 | Control |
| 3705_509.2179 | 1.366423714 | Control |
| 3706_509.2538 | 1.366423714 | Control |
| 3707_509.2531 | 1.366423714 | Control |
| 3708_509.2535 | 1.366423714 | Control |
| 3710_510.2872 | 1.366423714 | Control |
| 3712_510.3228 | 1.366423714 | Control |
| 3716_511.2329 | 1.366423714 | Control |
| 3720_511.3725 | 1.366423714 | Control |
| 3723_512.2657 | 1.366423714 | Control |
| 3724_512.2659 | 1.366423714 | Control |
| 3725_512.2662 | 1.366423714 | Control |
| 3726_512.3019 | 1.366423714 | Control |
| 3727_512.3019 | 1.366423714 | Control |
| 3728_512.3019 | 1.366423714 | Control |
| 3729_512.3023 | 1.366423714 | Control |
| 3733_513.226 | 1.366423714 | Control |
| 3734_513.2262 | 1.366423714 | Control |
| 3735_513.2627 | 1.366423714 | Control |
| 3736_513.2705 | 1.366423714 | Control |
| 3739_513.3888 | 1.366423714 | Control |
| 3740_514.2808 | 1.366423714 | Control |
| 3741_514.2809 | 1.366423714 | Control |
| 3742_514.281 | 1.366423714 | Control |
| 3743_514.2811 | 1.366423714 | Control |
| 3744_514.2813 | 1.366423714 | Control |
| 3745_514.3172 | 1.366423714 | Control |
| 3746_514.3176 | 1.366423714 | Control |
| 3748_515.2052 | 1.366423714 | Control |
| 3749_515.242 | 1.366423714 | Control |
| 3750_515.2422 | 1.366423714 | Control |
| 3751_515.2424 | 1.366423714 | Control |
| 3752_516.2603 | 1.366423714 | Control |
| 3753_516.2957 | 1.366423714 | Control |
| 3754_516.2955 | 1.366423714 | Control |
| 3755_516.2959 | 1.366423714 | Control |
| 3756_516.2962 | 1.366423714 | Control |
| 3758_517.2205 | 1.366423714 | Control |
| 3759_517.2214 | 1.366423714 | Control |
| 3760_517.2577 | 1.366423714 | Control |
| 3761_517.2636 | 1.366423714 | Control |
| 3765_518.2397 | 1.366423714 | Control |
| 3766_518.2584 | 1.366423714 | Control |
| 3767_518.2739 | 1.366423714 | Control |
| 3768_518.2754 | 1.366423714 | Control |
| 3769_518.2918 | 1.366423714 | Control |
| 3770_518.2918 | 1.366423714 | Control |
| 3774_519.2363 | 1.366423714 | Control |
| 3775_519.2364 | 1.366423714 | Control |
| 3776_519.2365 | 1.366423714 | Control |
| 3779_520.307 | 1.366423714 | Control |
| 3782_521.2157 | 1.366423714 | Control |
| 3785_522.2856 | 1.366423714 | Control |
| 3786_522.2863 | 1.366423714 | Control |
| 3787_522.2871 | 1.366423714 | Control |
| 3788_522.3236 | 1.366423714 | Control |
| 3789_522.3233 | 1.366423714 | Control |
| 3793_523.233 | 1.366423714 | Control |
| 3794_523.3173 | 1.366423714 | Control |
| 3795_524.2662 | 1.366423714 | Control |
| 3796_524.2659 | 1.366423714 | Control |
| 3797_524.2661 | 1.366423714 | Control |
| 3799_524.3386 | 1.366423714 | Control |
| 3800_524.3386 | 1.366423714 | Control |
| 3801_524.364 | 1.366423714 | Control |
| 3803_525.0354 | 1.366423714 | Control |
| 3804_526.2814 | 1.366423714 | Control |
| 3805_526.2815 | 1.366423714 | Control |
| 3806_526.2818 | 1.366423714 | Control |
| 3808_526.3175 | 1.366423714 | Control |
| 3809_526.3176 | 1.366423714 | Control |
| 3810_526.3176 | 1.366423714 | Control |
| 3811_526.3177 | 1.366423714 | Control |
| 3813_527.2647 | 1.366423714 | Control |
| 3814_527.2648 | 1.366423714 | Control |
| 3816_527.331 | 1.366423714 | Control |
| 3817_528.2523 | 1.366423714 | Control |
| 3818_528.259 | 1.366423714 | Control |
| 3819_528.2606 | 1.366423714 | Control |
| 3820_528.2607 | 1.366423714 | Control |
| 3822_528.2954 | 1.366423714 | Control |
| 3823_528.2965 | 1.366423714 | Control |
| 3824_528.297 | 1.366423714 | Control |
| 3825_528.2969 | 1.366423714 | Control |
| 3828_529.6688 | 1.366423714 | Control |
| 3829_530.2754 | 1.366423714 | Control |
| 3830_530.2754 | 1.366423714 | Control |
| 3831_530.2756 | 1.366423714 | Control |
| 3832_530.2757 | 1.366423714 | Control |
| 3834_530.312 | 1.366423714 | Control |
| 3835_531.2366 | 1.366423714 | Control |
| 3836_531.2368 | 1.366423714 | Control |
| 3837_531.2693 | 1.366423714 | Control |
| 3838_531.3119 | 1.366423714 | Control |
| 3839_532.2522 | 1.366423714 | Control |
| 3840_532.255 | 1.366423714 | Control |
| 3841_532.2904 | 1.366423714 | Control |
| 3843_533.2162 | 1.366423714 | Control |
| 3844_533.2813 | 1.366423714 | Control |
| 3846_534.2863 | 1.366423714 | Control |
| 3848_535.2102 | 1.366423714 | Control |
| 3851_536.3019 | 1.366423714 | Control |
| 3852_536.3026 | 1.366423714 | Control |
| 3853_536.3028 | 1.366423714 | Control |
| 3854_536.3031 | 1.366423714 | Control |
| 3855_536.304 | 1.366423714 | Control |
| 3856_537.2493 | 1.366423714 | Control |
| 3857_537.2495 | 1.366423714 | Control |
| 3859_538.2814 | 1.366423714 | Control |
| 3860_538.2814 | 1.366423714 | Control |
| 3861_538.3181 | 1.366423714 | Control |
| 3862_538.3182 | 1.366423714 | Control |
| 3863_538.3182 | 1.366423714 | Control |
| 3864_538.3184 | 1.366423714 | Control |
| 3865_538.3185 | 1.366423714 | Control |
| 3867_539.0505 | 1.366423714 | Control |
| 3868_539.3105 | 1.366423714 | Control |
| 3870_539.7107 | 1.366423714 | Control |
| 3872_540.2607 | 1.366423714 | Control |
| 3876_540.2964 | 1.366423714 | Control |
| 3877_540.2967 | 1.366423714 | Control |
| 3878_540.2971 | 1.366423714 | Control |
| 3879_540.3331 | 1.366423714 | Control |
| 3880_540.3333 | 1.366423714 | Control |
| 3883_541.2445 | 1.366423714 | Control |
| 3884_542.2758 | 1.366423714 | Control |
| 3885_542.2758 | 1.366423714 | Control |
| 3886_542.2761 | 1.366423714 | Control |
| 3887_542.2762 | 1.366423714 | Control |
| 3888_542.312 | 1.366423714 | Control |
| 3889_542.312 | 1.366423714 | Control |
| 3890_542.3123 | 1.366423714 | Control |
| 3891_542.3124 | 1.366423714 | Control |
| 3893_543.2599 | 1.366423714 | Control |
| 3894_543.323 | 1.366423714 | Control |
| 3897_544.2553 | 1.366423714 | Control |
| 3898_544.2913 | 1.366423714 | Control |
| 3899_544.291 | 1.366423714 | Control |
| 3900_544.2912 | 1.366423714 | Control |
| 3901_544.3448 | 1.366423714 | Control |
| 3903_545.3232 | 1.366423714 | Control |
| 3904_546.2697 | 1.366423714 | Control |
| 3905_546.2699 | 1.366423714 | Control |
| 3906_546.2706 | 1.366423714 | Control |
| 3907_546.2899 | 1.366423714 | Control |
| 3910_546.3748 | 1.366423714 | Control |
| 3915_548.2473 | 1.366423714 | Control |
| 3917_548.3386 | 1.366423714 | Control |
| 3919_549.2475 | 1.366423714 | Control |
| 3923_550.2812 | 1.366423714 | Control |
| 3924_550.3182 | 1.366423714 | Control |
| 3925_550.3184 | 1.366423714 | Control |
| 3928_552.2966 | 1.366423714 | Control |
| 3929_552.2973 | 1.366423714 | Control |
| 3930_552.2976 | 1.366423714 | Control |
| 3931_552.2978 | 1.366423714 | Control |
| 3934_553.2932 | 1.366423714 | Control |
| 3937_554.3116 | 1.366423714 | Control |
| 3938_554.3118 | 1.366423714 | Control |
| 3939_554.3119 | 1.366423714 | Control |
| 3940_554.4265 | 1.366423714 | Control |
| 3941_555.2589 | 1.366423714 | Control |
| 3945_556.2895 | 1.366423714 | Control |
| 3946_556.2907 | 1.366423714 | Control |
| 3947_556.3271 | 1.366423714 | Control |
| 3951_557.291 | 1.366423714 | Control |
| 3955_558.2694 | 1.366423714 | Control |
| 3956_558.306 | 1.366423714 | Control |
| 3957_558.3063 | 1.366423714 | Control |
| 3958_558.3064 | 1.366423714 | Control |
| 3962_560.2835 | 1.366423714 | Control |
| 3963_560.2846 | 1.366423714 | Control |
| 3964_560.2849 | 1.366423714 | Control |
| 3965_561.2462 | 1.366423714 | Control |
| 3967_562.2634 | 1.366423714 | Control |
| 3968_562.2646 | 1.366423714 | Control |
| 3969_562.2646 | 1.366423714 | Control |
| 3975_564.3323 | 1.366423714 | Control |
| 3978_566.3118 | 1.366423714 | Control |
| 3982_567.308 | 1.366423714 | Control |
| 3983_568.2909 | 1.366423714 | Control |
| 3984_568.2909 | 1.366423714 | Control |
| 3985_568.291 | 1.366423714 | Control |
| 3988_569.0608 | 1.366423714 | Control |
| 3990_570.3066 | 1.366423714 | Control |
| 3991_570.343 | 1.366423714 | Control |
| 3993_572.2863 | 1.366423714 | Control |
| 3994_572.3222 | 1.366423714 | Control |
| 3995_573.2854 | 1.366423714 | Control |
| 3998_574.2803 | 1.366423714 | Control |
| 4000_574.3008 | 1.366423714 | Control |
| 4003_576.2805 | 1.366423714 | Control |
| 4009_580.4419 | 1.366423714 | Control |
| 4010_581.2362 | 1.366423714 | Control |
| 4014_582.4579 | 1.366423714 | Control |
| 4016_583.7357 | 1.366423714 | Control |
| 4018_584.3223 | 1.366423714 | Control |
| 4022_586.3008 | 1.366423714 | Control |
| 4023_587.2642 | 1.366423714 | Control |
| 4025_588.3166 | 1.366423714 | Control |
| 4027_590.2753 | 1.366423714 | Control |
| 4030_594.307 | 1.366423714 | Control |
| 4033_596.285 | 1.366423714 | Control |
| 4034_596.3225 | 1.366423714 | Control |
| 4036_598.4528 | 1.366423714 | Control |
| 4044_603.2957 | 1.366423714 | Control |
| 4045_604.3121 | 1.366423714 | Control |
| 4047_605.3115 | 1.366423714 | Control |
| 4049_606.458 | 1.366423714 | Control |
| 4051_608.2852 | 1.366423714 | Control |
| 4056_610.2321 | 1.366423714 | Control |
| 4058_612.3171 | 1.366423714 | Control |
| 4064_615.2951 | 1.366423714 | Control |
| 4066_617.3114 | 1.366423714 | Control |
| 4067_617.3117 | 1.366423714 | Control |
| 4070_618.3064 | 1.366423714 | Control |
| 4071_619.3274 | 1.366423714 | Control |
| 4074_621.306 | 1.366423714 | Control |
| 4075_622.3383 | 1.366423714 | Control |
| 4076_623.3373 | 1.366423714 | Control |
| 4077_623.3878 | 1.366423714 | Control |
| 4079_624.3162 | 1.366423714 | Control |
| 4080_624.4683 | 1.366423714 | Control |
| 4082_626.4843 | 1.366423714 | Control |
| 4083_627.2942 | 1.366423714 | Control |
| 4095_636.3143 | 1.366423714 | Control |
| 4096_636.3167 | 1.366423714 | Control |
| 4097_638.2935 | 1.366423714 | Control |
| 4098_638.3322 | 1.366423714 | Control |
| 4099_638.3325 | 1.366423714 | Control |
| 4100_638.3326 | 1.366423714 | Control |
| 4102_640.3489 | 1.366423714 | Control |
| 4104_641.2716 | 1.366423714 | Control |
| 4105_641.3486 | 1.366423714 | Control |
| 4110_643.2879 | 1.366423714 | Control |
| 4114_645.4011 | 1.366423714 | Control |
| 4116_648.3544 | 1.366423714 | Control |
| 4119_650.3311 | 1.366423714 | Control |
| 4121_650.3707 | 1.366423714 | Control |
| 4122_650.4842 | 1.366423714 | Control |
| 4126_653.311 | 1.366423714 | Control |
| 4129_654.3268 | 1.366423714 | Control |
| 4131_655.3278 | 1.366423714 | Control |
| 4133_657.3046 | 1.366423714 | Control |
| 4140_662.3695 | 1.366423714 | Control |
| 4141_664.3393 | 1.366423714 | Control |
| 4142_664.3475 | 1.366423714 | Control |
| 4143_664.3476 | 1.366423714 | Control |
| 4145_666.3263 | 1.366423714 | Control |
| 4146_666.3656 | 1.366423714 | Control |
| 4147_666.3657 | 1.366423714 | Control |
| 4148_666.366 | 1.366423714 | Control |
| 4150_667.3253 | 1.366423714 | Control |
| 4153_668.3431 | 1.366423714 | Control |
| 4154_668.4948 | 1.366423714 | Control |
| 4156_670.3379 | 1.366423714 | Control |
| 4158_670.5104 | 1.366423714 | Control |
| 4163_675.2923 | 1.366423714 | Control |
| 4170_678.3641 | 1.366423714 | Control |
| 4171_678.3645 | 1.366423714 | Control |
| 4173_680.3429 | 1.366423714 | Control |
| 4174_680.3415 | 1.366423714 | Control |
| 4177_682.3577 | 1.366423714 | Control |
| 4178_683.3195 | 1.366423714 | Control |
| 4180_685.2978 | 1.366423714 | Control |
| 4181_685.298 | 1.366423714 | Control |
| 4182_685.3007 | 1.366423714 | Control |
| 4188_689.4925 | 1.366423714 | Control |
| 4189_690.4019 | 1.366423714 | Control |
| 4191_692.3809 | 1.366423714 | Control |
| 4192_692.3827 | 1.366423714 | Control |
| 4193_694.3577 | 1.366423714 | Control |
| 4194_694.358 | 1.366423714 | Control |
| 4195_694.5104 | 1.366423714 | Control |
| 4196_696.3756 | 1.366423714 | Control |
| 4205_703.3114 | 1.366423714 | Control |
| 4207_706.3957 | 1.366423714 | Control |
| 4208_706.396 | 1.366423714 | Control |
| 4209_706.3975 | 1.366423714 | Control |
| 4212_708.3752 | 1.366423714 | Control |
| 4213_708.3757 | 1.366423714 | Control |
| 4214_708.3757 | 1.366423714 | Control |
| 4216_710.3528 | 1.366423714 | Control |
| 4219_712.5211 | 1.366423714 | Control |
| 4221_714.5368 | 1.366423714 | Control |
| 4225_719.4457 | 1.366423714 | Control |
| 4229_727.4508 | 1.366423714 | Control |
| 4235_741.4591 | 1.366423714 | Control |
| 4239_749.4642 | 1.366423714 | Control |
| 4258_800.4002 | 1.366423714 | Control |
| 4259_802.4167 | 1.366423714 | Control |
| 4263_810.5581 | 1.366423714 | Control |
| 4266_828.4323 | 1.366423714 | Control |
| 4267_828.4327 | 1.366423714 | Control |
| 4270_830.4485 | 1.366423714 | Control |
| 4271_832.506 | 1.366423714 | Control |
| 4277_845.3875 | 1.366423714 | Control |
| 4286_872.6315 | 1.366423714 | Control |
| 4287_876.5322 | 1.366423714 | Control |
| 4295_980.5953 | 1.366423714 | Control |
| 4297_998.5053 | 1.366423714 | Control |
| 4298_1010.5275 | 1.366423714 | Control |
| 4299_1014.485 | 1.366423714 | Control |
| 4300_1015.4825 | 1.366423714 | Control |
| 4301_1026.5374 | 1.366423714 | Control |
| 4302_1038.5587 | 1.366423714 | Control |
| 721_185.1654 | 1.366207672 | H2O2 |
| 484_164.0709 | 1.36355057 | H2O2 |
| 437_159.113 | 1.362840891 | H2O2 |
| 1611_258.1703 | 1.362138962 | Control |
| 547_170.1179 | 1.359748066 | H2O2 |
| 1953_294.2066 | 1.358741981 | Control |
| 3134_408.3686 | 1.358566624 | Control |
| 410_156.1385 | 1.35760749 | H2O2 |
| 464_162.1028 | 1.357407937 | H2O2 |
| 1051_212.1032 | 1.357393536 | H2O2 |
| 3731_512.4169 | 1.356087819 | Control |
| 1236_226.1803 | 1.351779597 | Control |
| 187_129.1389 | 1.350023209 | H2O2 |
| 2741_362.2176 | 1.349910559 | Control |
| 1279_228.196 | 1.347095799 | Control |
| 339_150.128 | 1.34610147 | H2O2 |
| 2654_353.2187 | 1.345158849 | H2O2 |
| 1453_244.1545 | 1.345105952 | Control |
| 636_179.1069 | 1.342208483 | H2O2 |
| 908_201.1027 | 1.340468815 | H2O2 |
| 592_175.087 | 1.340171931 | H2O2 |
| 3086_404.2228 | 1.339093068 | H2O2 |
| 2321_327.2025 | 1.337104327 | H2O2 |
| 644_179.1547 | 1.336738251 | H2O2 |
| 3261_432.2382 | 1.335986607 | Control |
| 337_150.0917 | 1.335621473 | H2O2 |
| 271_141.1026 | 1.334868719 | H2O2 |
| 3544_482.4055 | 1.333800421 | Control |
| 1053_212.1396 | 1.33378768 | H2O2 |
| 20_97.0649 | 1.33342041 | H2O2 |
| 544_170.0815 | 1.333330668 | H2O2 |
| 285_142.123 | 1.332447518 | H2O2 |
| 2636_352.1983 | 1.332250855 | H2O2 |
| 732_187.087 | 1.331593281 | H2O2 |
| 1194_224.103 | 1.330990272 | H2O2 |
| 491_165.0662 | 1.330756964 | H2O2 |
| 282_142.0866 | 1.330362699 | H2O2 |
| 1049_211.1445 | 1.330260006 | H2O2 |
| 986_208.1084 | 1.32994236 | H2O2 |
| 3369_452.3949 | 1.328759448 | Control |
| 3216_423.4312 | 1.32874383 | H2O2 |
| 577_173.1078 | 1.328458058 | H2O2 |
| 2033_301.2851 | 1.328246151 | H2O2 |
| 1367_236.1396 | 1.327500909 | H2O2 |
| 1187_223.1443 | 1.326953058 | H2O2 |
| 1999_299.1718 | 1.326453195 | H2O2 |
| 476_163.0867 | 1.326048272 | H2O2 |
| 112_117.1025 | 1.325450283 | H2O2 |
| 532_169.0764 | 1.324228939 | H2O2 |
| 3636_496.4213 | 1.322256187 | Control |
| 3293_438.2699 | 1.321172107 | H2O2 |
| 926_202.1806 | 1.320889575 | H2O2 |
| 260_140.1074 | 1.320167308 | H2O2 |
| 1599_257.139 | 1.320110486 | H2O2 |
| 336_150.0917 | 1.316220857 | H2O2 |
| 3541_482.2963 | 1.315618543 | H2O2 |
| 3992_570.458 | 1.315348547 | Control |
| 24_98.5122 | 1.312260451 | Control |
| 643_179.1547 | 1.311705024 | H2O2 |
| 3174_415.212 | 1.310463067 | Control |
| 3812_526.4326 | 1.309954004 | Control |
| 2379_330.3369 | 1.309914336 | Control |
| 448_160.0871 | 1.309596267 | H2O2 |
| 1796_279.1458 | 1.309560368 | H2O2 |
| 3949_556.4423 | 1.309439588 | Control |
| 645_180.0771 | 1.308585122 | H2O2 |
| 210_135.0557 | 1.308403763 | H2O2 |
| 309_147.092 | 1.306873651 | H2O2 |
| 1998_299.1718 | 1.306702987 | H2O2 |
| 3281_436.2545 | 1.30562938 | H2O2 |
| 1021_210.124 | 1.305558064 | H2O2 |
| 2993_392.2283 | 1.305443913 | H2O2 |
| 3410_460.2695 | 1.305247757 | Control |
| 2805_368.1935 | 1.30441763 | H2O2 |
| 2477_339.1417 | 1.304295546 | Control |
| 152_125.0713 | 1.303750756 | H2O2 |
| 584_174.0917 | 1.302549435 | H2O2 |
| 574_173.1077 | 1.301326583 | H2O2 |
| 1462_244.2273 | 1.301203603 | Control |
| 702_183.1497 | 1.301189543 | H2O2 |
| 192_130.123 | 1.301070961 | H2O2 |
| 259_140.1074 | 1.300419607 | H2O2 |
| 295_144.1023 | 1.30024903 | H2O2 |
| 527_167.1546 | 1.296916806 | H2O2 |
| 1308_230.1753 | 1.296259889 | Control |
| 90_113.0711 | 1.29602622 | H2O2 |
| 2314_327.1416 | 1.295447344 | Control |
| 1673_265.1298 | 1.294912214 | H2O2 |
| 1217_225.1598 | 1.294783759 | H2O2 |
| 432_159.0918 | 1.294360701 | H2O2 |
| 247_139.087 | 1.294018252 | H2O2 |
| 1141_218.2117 | 1.292074231 | Control |
| 1640_261.1236 | 1.290299455 | H2O2 |
| 537_169.0972 | 1.284389284 | H2O2 |
| 130_123.0556 | 1.284383185 | H2O2 |
| 501_165.1026 | 1.28362873 | H2O2 |
| 480_163.1231 | 1.283598351 | H2O2 |
| 2073_304.2485 | 1.283314113 | Control |
| 334_150.0553 | 1.282609644 | H2O2 |
| 523_167.1182 | 1.281343775 | H2O2 |
| 186_129.1025 | 1.280290084 | H2O2 |
| 705_184.1337 | 1.280034116 | H2O2 |
| 435_159.0919 | 1.280012633 | H2O2 |
| 2547_344.3161 | 1.279847674 | H2O2 |
| 170_127.0869 | 1.279779411 | H2O2 |
| 310_147.092 | 1.279330694 | H2O2 |
| 1397_239.1545 | 1.279110172 | H2O2 |
| 416_157.0974 | 1.275624439 | H2O2 |
| 429_158.9615 | 1.274230216 | Control |
| 548_170.1541 | 1.272509786 | H2O2 |
| 228_137.0714 | 1.272161257 | H2O2 |
| 2025_301.1652 | 1.271088971 | H2O2 |
| 220_136.0761 | 1.2701371 | H2O2 |
| 169_127.0869 | 1.270127052 | H2O2 |
| 225_136.1125 | 1.26941372 | H2O2 |
| 317_148.0873 | 1.268764518 | H2O2 |
| 2699_358.2231 | 1.267763619 | H2O2 |
| 358_152.1073 | 1.267588621 | H2O2 |
| 921_202.1231 | 1.266929559 | H2O2 |
| 553_171.1019 | 1.266481559 | Control |
| 165_126.0917 | 1.265197898 | H2O2 |
| 958_206.0929 | 1.263589749 | H2O2 |
| 564_172.0971 | 1.263075321 | H2O2 |
| 446_160.076 | 1.262080047 | H2O2 |
| 1843_286.1404 | 1.261893386 | H2O2 |
| 273_141.1026 | 1.261575157 | H2O2 |
| 1615_258.2066 | 1.260895731 | Control |
| 298_145.0764 | 1.260352299 | H2O2 |
| 632_179.0819 | 1.258779903 | H2O2 |
| 2435_335.1716 | 1.258392447 | H2O2 |
| 896_200.1075 | 1.258007658 | H2O2 |
| 2143_311.1718 | 1.255068044 | H2O2 |
| 3262_432.2596 | 1.254782818 | H2O2 |
| 3540_482.2963 | 1.254629105 | H2O2 |
| 300_145.0764 | 1.252869443 | H2O2 |
| 838_195.1385 | 1.252199517 | Control |
| 422_158.1178 | 1.250971691 | H2O2 |
| 1662_264.1326 | 1.250530822 | H2O2 |
| 1961_295.1767 | 1.247817056 | H2O2 |
| 607_176.1186 | 1.247646563 | H2O2 |
| 1442_243.1232 | 1.246889395 | H2O2 |
| 3053_399.2511 | 1.246776044 | Control |
| 3033_396.2233 | 1.245461637 | H2O2 |
| 1971_297.1562 | 1.244606298 | H2O2 |
| 602_176.0822 | 1.244521282 | H2O2 |
| 3101_406.2439 | 1.244498277 | Control |
| 457_161.1075 | 1.244124542 | H2O2 |
| 985_208.1084 | 1.243873781 | H2O2 |
| 1082_214.1553 | 1.243606869 | H2O2 |
| 4029_592.3 | 1.243561128 | Control |
| 928_203.0821 | 1.24356024 | H2O2 |
| 877_199.1082 | 1.243233248 | H2O2 |
| 1071_213.1489 | 1.242945521 | Control |
| 330_149.0713 | 1.241583474 | H2O2 |
| 164_126.0917 | 1.240111373 | H2O2 |
| 661_181.1226 | 1.240087889 | H2O2 |
| 682_183.0769 | 1.239999431 | H2O2 |
| 1586_256.1658 | 1.238154125 | H2O2 |
| 1437_243.0979 | 1.23797115 | H2O2 |
| 528_168.1023 | 1.237924605 | H2O2 |
| 2987_391.2864 | 1.237444799 | Control |
| 1382_238.1186 | 1.236243856 | H2O2 |
| 1612_258.1816 | 1.236162686 | H2O2 |
| 2019_300.2899 | 1.23595568 | H2O2 |
| 190_130.0866 | 1.235161279 | H2O2 |
| 1065_213.1237 | 1.2350072 | H2O2 |
| 894_200.1034 | 1.234687491 | H2O2 |
| 97_114.0916 | 1.233672291 | H2O2 |
| 990_208.1448 | 1.233535797 | H2O2 |
| 1263_227.2007 | 1.233489437 | Control |
| 1323_232.1083 | 1.232947346 | H2O2 |
| 599_175.1234 | 1.232087139 | H2O2 |
| 74_110.0603 | 1.230710579 | H2O2 |
| 456_161.1075 | 1.23066986 | H2O2 |
| 36_102.0915 | 1.228394663 | H2O2 |
| 252_139.1234 | 1.227489189 | H2O2 |
| 890_199.1447 | 1.226581952 | H2O2 |
| 1098_215.1546 | 1.226150982 | H2O2 |
| 266_140.1187 | 1.22612181 | H2O2 |
| 1892_289.1653 | 1.225840691 | H2O2 |
| 2010_300.1561 | 1.224030232 | H2O2 |
| 2835_372.1977 | 1.223377678 | Control |
| 327_149.0712 | 1.223075984 | H2O2 |
| 503_165.1389 | 1.222978603 | H2O2 |
| 2020_300.2899 | 1.222346334 | H2O2 |
| 1252_227.1393 | 1.222254707 | H2O2 |
| 371_153.1025 | 1.221379905 | H2O2 |
| 272_141.1026 | 1.220971245 | H2O2 |
| 1532_250.1551 | 1.220294034 | H2O2 |
| 849_196.1086 | 1.220040617 | H2O2 |
| 248_139.087 | 1.219582596 | H2O2 |
| 713_185.1289 | 1.21956763 | H2O2 |
| 3294_438.2701 | 1.217769289 | H2O2 |
| 374_153.1389 | 1.21662226 | H2O2 |
| 1058_213.0874 | 1.214131049 | H2O2 |
| 765_190.098 | 1.213917574 | H2O2 |
| 2207_316.2123 | 1.212067193 | H2O2 |
| 400_156.0658 | 1.210007685 | H2O2 |
| 1844_286.1405 | 1.209183284 | H2O2 |
| 3821_528.2708 | 1.209035496 | Control |
| 813_193.1705 | 1.208513964 | H2O2 |
| 452_160.0969 | 1.206805861 | H2O2 |
| 1207_225.1236 | 1.206372911 | H2O2 |
| 397_155.1181 | 1.205290074 | H2O2 |
| 333_149.1076 | 1.205177375 | H2O2 |
| 2126_309.2272 | 1.203620418 | Control |
| 1676_265.6582 | 1.203544278 | Control |
| 631_179.0819 | 1.202741689 | H2O2 |
| 3610_492.7505 | 1.201904694 | Control |
| 1685_267.1956 | 1.200828595 | Control |
| 966_206.1907 | 1.200074535 | H2O2 |
| 238_137.1078 | 1.198537586 | H2O2 |
| 236_137.1077 | 1.198108417 | H2O2 |
| 2101_306.1918 | 1.196574672 | Control |
| 1316_231.1232 | 1.196302749 | H2O2 |
| 3626_494.2961 | 1.194985658 | H2O2 |
| 4168_677.334 | 1.194434606 | Control |
| 943_204.1386 | 1.194094211 | H2O2 |
| 399_156.0658 | 1.193880965 | H2O2 |
| 2939_384.2957 | 1.193171497 | Control |
| 540_169.1338 | 1.193152422 | H2O2 |
| 1641_261.1236 | 1.193022325 | H2O2 |
| 3323_442.3376 | 1.19184859 | Control |
| 275_141.139 | 1.190636905 | H2O2 |
| 401_156.0658 | 1.1904683 | H2O2 |
| 2298_325.2223 | 1.190100894 | Control |
| 860_197.0926 | 1.190049152 | H2O2 |
| 72_110.0603 | 1.189496666 | H2O2 |
| 738_187.1235 | 1.189074155 | H2O2 |
| 2442_335.224 | 1.188342652 | Control |
| 2852_374.2177 | 1.188065969 | Control |
| 814_193.1705 | 1.187794497 | H2O2 |
| 4243_766.5319 | 1.187444883 | Control |
| 389_155.0818 | 1.187131702 | H2O2 |
| 933_204.1026 | 1.186477243 | H2O2 |
| 3950_556.7807 | 1.185978705 | Control |
| 1227_226.1188 | 1.184413696 | H2O2 |
| 2045_302.208 | 1.183293039 | Control |
| 3396_457.3288 | 1.181742413 | Control |
| 767_190.0981 | 1.181593591 | H2O2 |
| 2084_305.2147 | 1.181007514 | Control |
| 2463_337.1871 | 1.180797485 | H2O2 |
| 1741_272.2587 | 1.180319155 | Control |
| 404_156.1022 | 1.180297574 | H2O2 |
| 3926_550.4071 | 1.179926187 | Control |
| 1270_228.1344 | 1.17985698 | H2O2 |
| 1579_255.1958 | 1.179439718 | Control |
| 3896_543.3993 | 1.178048759 | Control |
| 1705_270.1451 | 1.177981265 | H2O2 |
| 3667_500.3795 | 1.177443918 | Control |
| 657_181.0975 | 1.177310841 | H2O2 |
| 3215_423.2767 | 1.177285024 | Control |
| 2499_341.1963 | 1.177099593 | H2O2 |
| 2655_353.2187 | 1.177091906 | H2O2 |
| 1559_253.1914 | 1.176375082 | H2O2 |
| 1029_211.0968 | 1.175277679 | Control |
| 356_152.0709 | 1.174517412 | H2O2 |
| 1941_293.161 | 1.174296586 | H2O2 |
| 2178_314.187 | 1.17365789 | Control |
| 2310_326.2539 | 1.173145931 | Control |
| 929_203.1393 | 1.171997516 | H2O2 |
| 3539_482.2933 | 1.17169473 | Control |
| 2839_372.2299 | 1.171314668 | Control |
| 269_141.0914 | 1.171196533 | Control |
| 2778_365.1364 | 1.171155393 | Control |
| 2756_363.2565 | 1.171095354 | Control |
| 1959_295.1532 | 1.170963845 | Control |
| 2754_363.2202 | 1.170419628 | Control |
| 1723_271.6584 | 1.170085766 | Control |
| 4183_686.5416 | 1.169983505 | Control |
| 25_99.0806 | 1.169463554 | H2O2 |
| 3194_420.2595 | 1.1691284 | H2O2 |
| 1755_274.2016 | 1.169034673 | Control |
| 1383_238.1188 | 1.16898612 | H2O2 |
| 2619_350.2027 | 1.168912917 | Control |
| 893_199.1697 | 1.168769198 | H2O2 |
| 211_135.0807 | 1.168285238 | Control |
| 1645_261.1924 | 1.168191427 | H2O2 |
| 123_120.0811 | 1.167919168 | H2O2 |
| 4226_722.5053 | 1.167905442 | Control |
| 4237_746.5633 | 1.167461273 | Control |
| 1030_211.0968 | 1.166677988 | Control |
| 3784_521.386 | 1.166576912 | Control |
| 1563_254.15 | 1.166481851 | H2O2 |
| 707_185.0925 | 1.166262722 | H2O2 |
| 3100_406.2263 | 1.165895446 | Control |
| 3826_528.3935 | 1.165834868 | Control |
| 4224_716.5523 | 1.164899295 | Control |
| 3272_434.2747 | 1.164266696 | H2O2 |
| 1774_277.1284 | 1.164151145 | Control |
| 601_176.0822 | 1.163812591 | H2O2 |
| 3402_458.3219 | 1.163778938 | Control |
| 4264_818.4014 | 1.163458408 | Control |
| 292_144.1023 | 1.162649062 | H2O2 |
| 874_198.1857 | 1.162539334 | Control |
| 4255_796.5425 | 1.162325314 | Control |
| 3401_458.2894 | 1.162067216 | Control |
| 4108_642.5155 | 1.162037988 | Control |
| 689_183.102 | 1.161470104 | Control |
| 626_178.123 | 1.161217892 | H2O2 |
| 3382_454.7527 | 1.161053401 | Control |
| 2698_358.2227 | 1.16083254 | H2O2 |
| 2760_364.1997 | 1.160474586 | Control |
| 742_187.1446 | 1.160387715 | H2O2 |
| 4303_1040.6321 | 1.160308017 | Control |
| 3527_480.3369 | 1.160183721 | Control |
| 2612_349.195 | 1.159818631 | Control |
| 538_169.0973 | 1.159227192 | H2O2 |
| 3693_506.3805 | 1.159132278 | Control |
| 792_192.1026 | 1.159077328 | H2O2 |
| 638_179.1183 | 1.158631657 | H2O2 |
| 3182_416.2676 | 1.157480242 | Control |
| 694_183.1133 | 1.15653941 | H2O2 |
| 508_166.0866 | 1.156308101 | H2O2 |
| 3358_450.2906 | 1.155458965 | Control |
| 3747_514.3776 | 1.155105198 | Control |
| 4172_678.4792 | 1.154430512 | Control |
| 2278_324.1417 | 1.154065779 | Control |
| 4041_601.3748 | 1.153894356 | Control |
| 3184_417.3149 | 1.15318854 | Control |
| 4204_702.5367 | 1.153048946 | Control |
| 4052_608.3004 | 1.152918786 | Control |
| 205_133.0764 | 1.152850341 | H2O2 |
| 1348_234.1338 | 1.152258612 | Control |
| 3737_513.2737 | 1.152165274 | Control |
| 230_137.0714 | 1.151690219 | H2O2 |
| 579_173.1288 | 1.151479334 | H2O2 |
| 2523_343.1885 | 1.150824997 | Control |
| 328_149.0712 | 1.150796218 | H2O2 |
| 2259_321.1443 | 1.150617882 | Control |
| 1083_214.1553 | 1.150526067 | H2O2 |
| 1036_211.1081 | 1.15014174 | H2O2 |
| 3560_484.3666 | 1.149538484 | Control |
| 3062_400.2907 | 1.148876001 | Control |
| 4217_711.5057 | 1.148684845 | Control |
| 651_180.1386 | 1.148508266 | H2O2 |
| 2591_347.2616 | 1.148351658 | Control |
| 2561_345.2461 | 1.148295379 | Control |
| 3028_395.3999 | 1.148154721 | H2O2 |
| 3412_460.2944 | 1.148023151 | Control |
| 2213_316.2849 | 1.147802959 | Control |
| 3609_492.3641 | 1.146870693 | Control |
| 331_149.1076 | 1.146444412 | H2O2 |
| 1403_240.1344 | 1.146265922 | H2O2 |
| 3286_436.2903 | 1.145963785 | Control |
| 2281_324.2023 | 1.145768233 | H2O2 |
| 1487_246.1815 | 1.145757439 | Control |
| 1497_247.1681 | 1.145625457 | Control |
| 541_169.1338 | 1.145372206 | H2O2 |
| 1719_271.1769 | 1.145352968 | H2O2 |
| 712_185.1289 | 1.145243141 | H2O2 |
| 195_130.1343 | 1.144523585 | H2O2 |
| 4135_658.5105 | 1.144071011 | Control |
| 2867_376.2332 | 1.143634922 | Control |
| 1790_278.2117 | 1.143292292 | Control |
| 2966_388.2335 | 1.143202005 | H2O2 |
| 3692_506.3334 | 1.14312129 | Control |
| 3326_443.2331 | 1.143065301 | Control |
| 3432_463.3429 | 1.14256507 | Control |
| 2601_348.2024 | 1.14248989 | H2O2 |
| 3045_398.2404 | 1.142477812 | H2O2 |
| 3480_471.195 | 1.142114164 | Control |
| 2167_313.1872 | 1.142041483 | H2O2 |
| 1717_271.1543 | 1.141690108 | Control |
| 4244_767.8857 | 1.141279241 | Control |
| 81_112.0759 | 1.140976525 | H2O2 |
| 3317_441.3298 | 1.140938842 | Control |
| 277_141.9589 | 1.140471874 | Control |
| 2487_339.2895 | 1.140458331 | Control |
| 265_140.1186 | 1.139543688 | H2O2 |
| 883_199.1237 | 1.139325716 | H2O2 |
| 3347_448.3378 | 1.138542538 | Control |
| 1027_210.1604 | 1.138497754 | H2O2 |
| 1264_227.2008 | 1.137959771 | Control |
| 843_195.1497 | 1.137706219 | H2O2 |
| 1862_287.1627 | 1.137281215 | Control |
| 3479_470.351 | 1.136779388 | Control |
| 1381_238.0898 | 1.136675718 | Control |
| 1740_272.2587 | 1.136628457 | Control |
| 3186_418.244 | 1.136328975 | Control |
| 3689_506.2614 | 1.135749151 | Control |
| 4048_606.3858 | 1.134889418 | Control |
| 4162_674.3512 | 1.134726766 | Control |
| 3233_425.2514 | 1.134541542 | Control |
| 3162_413.2512 | 1.134398244 | H2O2 |
| 3651_498.7783 | 1.134279503 | Control |
| 3177_415.2501 | 1.133669529 | Control |
| 133_123.0806 | 1.133539795 | Control |
| 859_197.0926 | 1.132974327 | H2O2 |
| 2848_373.2773 | 1.132903891 | Control |
| 1232_226.1552 | 1.132637679 | H2O2 |
| 4185_687.4443 | 1.132631791 | Control |
| 3676_503.2661 | 1.132078262 | Control |
| 611_176.1438 | 1.131982217 | H2O2 |
| 1808_282.1512 | 1.131677048 | Control |
| 4233_739.3828 | 1.130901731 | Control |
| 2964_388.2125 | 1.130614689 | Control |
| 2822_370.7067 | 1.130107441 | Control |
| 1410_241.1065 | 1.13002669 | Control |
| 2568_346.2226 | 1.12999342 | Control |
| 2102_306.2179 | 1.12981972 | Control |
| 961_206.1292 | 1.129733413 | H2O2 |
| 4035_598.3591 | 1.129550374 | Control |
| 4296_996.6059 | 1.128469062 | Control |
| 3235_425.7308 | 1.128349178 | Control |
| 2083_305.1791 | 1.127826551 | Control |
| 3422_462.2716 | 1.127729706 | Control |
| 1406_240.1707 | 1.127623469 | H2O2 |
| 3512_477.301 | 1.127221165 | H2O2 |
| 3684_505.2439 | 1.127118594 | Control |
| 407_156.1133 | 1.126979958 | H2O2 |
| 80_111.1171 | 1.126927811 | Control |
| 3078_402.7041 | 1.126797995 | Control |
| 3607_492.3169 | 1.126661844 | Control |
| 61_107.9669 | 1.126329199 | Control |
| 1224_226.1186 | 1.125692807 | H2O2 |
| 2203_316.1756 | 1.125541599 | Control |
| 372_153.1025 | 1.124609234 | H2O2 |
| 802_193.0977 | 1.124268251 | H2O2 |
| 4304_1058.0665 | 1.124027004 | Control |
| 764_190.0979 | 1.123805733 | H2O2 |
| 2797_367.1528 | 1.123587347 | Control |
| 4084_627.4584 | 1.123203228 | Control |
| 620_177.1277 | 1.123134897 | H2O2 |
| 290_143.9971 | 1.123090265 | Control |
| 2251_320.1711 | 1.122278666 | H2O2 |
| 3270_433.7411 | 1.122117136 | Control |
| 3302_439.2163 | 1.121065321 | Control |
| 741_187.1445 | 1.121062306 | H2O2 |
| 3051_399.218 | 1.120800783 | Control |
| 143_123.0919 | 1.120507272 | H2O2 |
| 109_116.1072 | 1.12048919 | H2O2 |
| 2421_334.2225 | 1.120470345 | Control |
| 4262_810.4028 | 1.120302913 | Control |
| 3570_486.268 | 1.120232517 | Control |
| 3597_490.2797 | 1.119494202 | Control |
| 3246_427.5744 | 1.11943244 | Control |
| 3169_414.72 | 1.11940412 | Control |
| 3289_437.1934 | 1.119063015 | Control |
| 2564_346.1803 | 1.119000142 | Control |
| 2787_366.2118 | 1.118746118 | Control |
| 4011_581.2903 | 1.118299658 | Control |
| 2960_387.2064 | 1.117515917 | Control |
| 1426_242.1502 | 1.117344386 | H2O2 |
| 906_201.1004 | 1.117321685 | Control |
| 4123_651.8265 | 1.116483007 | Control |
| 2802_367.1854 | 1.116071776 | Control |
| 94_114.0915 | 1.116024959 | Control |
| 1665_264.1812 | 1.11586996 | H2O2 |
| 367_153.0913 | 1.115587769 | Control |
| 2475_339.0761 | 1.115113428 | Control |
| 3324_442.7196 | 1.114969069 | Control |
| 4187_689.3967 | 1.114604316 | Control |
| 2200_315.2032 | 1.1145173 | H2O2 |
| 4134_658.3224 | 1.114506221 | Control |
| 3228_425.2147 | 1.114200796 | Control |
| 58_105.0701 | 1.114086695 | H2O2 |
| 342_151.0869 | 1.11404938 | H2O2 |
| 2161_313.1438 | 1.113586209 | Control |
| 4291_923.454 | 1.113283846 | Control |
| 185_129.1025 | 1.113256769 | H2O2 |
| 3578_487.2702 | 1.112864445 | Control |
| 939_204.1234 | 1.112595309 | Control |
| 3446_466.3011 | 1.112500972 | H2O2 |
| 3168_414.7198 | 1.112420626 | Control |
| 2535_344.1861 | 1.112412117 | Control |
| 4201_698.8769 | 1.111919401 | Control |
| 3431_463.2482 | 1.111444952 | Control |
| 3275_434.7361 | 1.110445469 | Control |
| 2566_346.1873 | 1.109887501 | Control |
| 4091_634.3281 | 1.109845086 | Control |
| 904_200.2014 | 1.109258292 | Control |
| 1282_228.2324 | 1.109062605 | Control |
| 845_195.1692 | 1.108925169 | Control |
| 2689_357.1474 | 1.108718113 | Control |
| 671_182.1181 | 1.108564003 | H2O2 |
| 4157_670.4175 | 1.108265794 | Control |
| 533_169.0861 | 1.108205403 | Control |
| 2880_377.2359 | 1.107571386 | Control |
| 2735_362.1522 | 1.106799535 | Control |
| 351_151.1233 | 1.106092207 | H2O2 |
| 3430_463.2475 | 1.105580677 | Control |
| 256_140.071 | 1.105574948 | H2O2 |
| 1543_251.1856 | 1.104404111 | Control |
| 444_160.0759 | 1.10413846 | H2O2 |
| 2823_371.0927 | 1.103979158 | Control |
| 1407_240.1708 | 1.103884113 | H2O2 |
| 617_177.1026 | 1.103221394 | H2O2 |
| 377_154.0865 | 1.102911031 | H2O2 |
| 2193_315.1231 | 1.102763951 | Control |
| 396_155.1181 | 1.102750127 | H2O2 |
| 1813_282.1967 | 1.102726001 | H2O2 |
| 934_204.1027 | 1.102270853 | H2O2 |
| 2110_307.194 | 1.101918066 | Control |
| 2507_342.2493 | 1.101465549 | Control |
| 3234_425.2701 | 1.100639815 | Control |
| 1556_253.1698 | 1.100118924 | H2O2 |
| 2752_363.201 | 1.099964301 | Control |
| 4169_678.3211 | 1.099935388 | Control |
| 4238_747.6992 | 1.099807601 | Control |
| 2901_379.2105 | 1.099368629 | Control |
| 3322_442.2416 | 1.09928068 | Control |
| 3732_512.7635 | 1.099119975 | Control |
| 1438_243.0996 | 1.099099972 | Control |
| 1249_227.1392 | 1.099039526 | H2O2 |
| 585_174.0917 | 1.09901948 | H2O2 |
| 2068_304.1757 | 1.098954146 | Control |
| 4282_857.4205 | 1.098745296 | Control |
| 3335_446.2539 | 1.098618409 | Control |
| 2201_315.6736 | 1.098032942 | Control |
| 4184_687.4442 | 1.097966258 | Control |
| 2170_313.1994 | 1.097963119 | Control |
| 925_202.1443 | 1.097893847 | Control |
| 3043_398.2221 | 1.097758134 | Control |
| 3063_400.2925 | 1.097675439 | Control |
| 2931_383.2643 | 1.09758085 | Control |
| 2003_299.2076 | 1.097363106 | H2O2 |
| 1167_221.1651 | 1.097022499 | H2O2 |
| 2639_352.2332 | 1.096903242 | Control |
| 1600_257.1579 | 1.096750904 | Control |
| 1054_212.1396 | 1.096683239 | H2O2 |
| 640_179.1183 | 1.094836058 | H2O2 |
| 677_182.9855 | 1.094378049 | Control |
| 4118_648.8229 | 1.094295581 | Control |
| 2544_344.2337 | 1.094241809 | Control |
| 3200_421.2304 | 1.093759204 | Control |
| 3598_490.7681 | 1.093585728 | Control |
| 3199_421.2097 | 1.093220669 | Control |
| 1683_267.1699 | 1.092914038 | Control |
| 4117_648.4078 | 1.092867534 | Control |
| 2638_352.2331 | 1.092574347 | Control |
| 2127_309.2791 | 1.092195388 | Control |
| 2393_332.1497 | 1.092033466 | Control |
| 4167_676.4394 | 1.091820312 | Control |
| 2903_379.6934 | 1.091818259 | Control |
| 1465_245.0811 | 1.091308364 | Control |
| 308_146.1179 | 1.090736263 | H2O2 |
| 4057_611.8091 | 1.090380368 | Control |
| 4273_840.4697 | 1.089893946 | Control |
| 3845_533.3268 | 1.089656348 | Control |
| 213_135.1019 | 1.089501895 | Control |
| 4242_762.0549 | 1.089494768 | Control |
| 1678_266.1605 | 1.089261499 | H2O2 |
| 2191_315.123 | 1.088817184 | Control |
| 593_175.087 | 1.088377462 | H2O2 |
| 947_205.0863 | 1.088301594 | H2O2 |
| 827_194.1756 | 1.087918232 | Control |
| 286_142.1594 | 1.087822938 | H2O2 |
| 50_102.1279 | 1.086768254 | Control |
| 872_198.1494 | 1.086681225 | H2O2 |
| 2814_369.2752 | 1.086225317 | Control |
| 3966_561.9464 | 1.086089964 | Control |
| 2983_390.249 | 1.086018625 | Control |
| 4060_612.9598 | 1.08593829 | Control |
| 1789_278.1904 | 1.085848903 | Control |
| 1646_261.2426 | 1.085657503 | Control |
| 3146_410.2941 | 1.085554427 | Control |
| 3637_496.7627 | 1.085031722 | Control |
| 3519_478.3223 | 1.084977801 | Control |
| 3267_433.2169 | 1.084836426 | Control |
| 1137_218.1542 | 1.084426606 | H2O2 |
| 718_185.1653 | 1.083939519 | Control |
| 3219_424.2525 | 1.083890635 | Control |
| 2257_320.2434 | 1.083594161 | Control |
| 3791_522.3646 | 1.083580576 | Control |
| 4289_906.9492 | 1.08353375 | Control |
| 868_197.154 | 1.083484983 | Control |
| 2648_352.2337 | 1.08329206 | Control |
| 4020_584.4272 | 1.083213115 | Control |
| 3458_468.29 | 1.083086438 | Control |
| 3529_480.7327 | 1.082722821 | Control |
| 4106_642.3862 | 1.082107971 | Control |
| 4062_614.3546 | 1.081602127 | Control |
| 1506_248.1391 | 1.081460799 | Control |
| 4160_672.4112 | 1.081393798 | H2O2 |
| 216_136.0219 | 1.081193372 | Control |
| 1422_241.2038 | 1.08052571 | Control |
| 1744_273.17 | 1.080501114 | Control |
| 2401_332.2071 | 1.080131588 | Control |
| 855_196.145 | 1.079579757 | H2O2 |
| 4001_575.2727 | 1.07952735 | Control |
| 415_157.0973 | 1.078986484 | H2O2 |
| 2489_339.6643 | 1.078929564 | Control |
| 3960_558.9425 | 1.078902242 | Control |
| 3927_551.347 | 1.078885834 | H2O2 |
| 1368_236.1496 | 1.078720056 | Control |
| 1558_253.1913 | 1.07860575 | Control |
| 913_201.1392 | 1.078586352 | H2O2 |
| 1591_256.1762 | 1.078431358 | Control |
| 4137_659.413 | 1.078320092 | Control |
| 649_180.1023 | 1.078058983 | Control |
| 2607_349.0369 | 1.078054938 | Control |
| 2036_301.2934 | 1.077707969 | Control |
| 586_174.103 | 1.076682248 | H2O2 |
| 1283_228.2324 | 1.076413351 | Control |
| 4089_631.3813 | 1.076235536 | Control |
| 2606_348.2745 | 1.075528072 | Control |
| 2670_355.2344 | 1.074805884 | H2O2 |
| 2021_300.3265 | 1.074665402 | Control |
| 1884_288.2899 | 1.072723461 | Control |
| 156_125.1077 | 1.072454099 | H2O2 |
| 3763_517.6109 | 1.07241451 | Control |
| 3715_511.2268 | 1.071919078 | Control |
| 1727_272.1609 | 1.071839816 | H2O2 |
| 403_156.1021 | 1.071790926 | H2O2 |
| 2443_336.1551 | 1.071613844 | Control |
| 4281_850.4138 | 1.071374109 | Control |
| 3264_432.7567 | 1.07131928 | Control |
| 1942_293.1614 | 1.070998079 | H2O2 |
| 209_135.0556 | 1.07090379 | H2O2 |
| 3342_448.2565 | 1.070601576 | Control |
| 1070_213.1393 | 1.070491285 | H2O2 |
| 447_160.0761 | 1.07043236 | H2O2 |
| 1313_231.1109 | 1.07042237 | Control |
| 2403_332.2798 | 1.070277641 | Control |
| 1745_273.1707 | 1.070260516 | Control |
| 1653_262.1655 | 1.069567999 | H2O2 |
| 1837_285.1925 | 1.069334715 | H2O2 |
| 3397_458.2071 | 1.069092008 | Control |
| 4026_588.9647 | 1.068457645 | Control |
| 1439_243.1129 | 1.068336532 | H2O2 |
| 884_199.1237 | 1.067873043 | H2O2 |
| 589_174.1032 | 1.067798384 | H2O2 |
| 460_162.0664 | 1.06776905 | H2O2 |
| 2803_367.2692 | 1.067661286 | Control |
| 1373_237.1276 | 1.067167755 | Control |
| 2538_344.2256 | 1.066291912 | Control |
| 3694_506.7636 | 1.065854635 | Control |
| 1138_218.1865 | 1.065805277 | Control |
| 1991_298.3107 | 1.065081016 | Control |
| 2847_373.2452 | 1.065013546 | Control |
| 3447_466.3173 | 1.064990583 | Control |
| 775_190.1344 | 1.064643112 | H2O2 |
| 3022_394.2443 | 1.064341092 | H2O2 |
| 1840_285.1926 | 1.064163899 | Control |
| 948_205.0975 | 1.064043313 | Control |
| 1428_242.1543 | 1.06384576 | Control |
| 3981_567.278 | 1.063738898 | Control |
| 2703_358.2387 | 1.062940133 | Control |
| 795_192.1386 | 1.0626388 | H2O2 |
| 294_144.1023 | 1.06261881 | H2O2 |
| 2592_347.3158 | 1.062482918 | Control |
| 1284_228.2688 | 1.062426824 | Control |
| 1905_290.1603 | 1.060845076 | Control |
| 338_150.1029 | 1.060050788 | H2O2 |
| 2356_329.1822 | 1.060031238 | Control |
| 1109_216.1388 | 1.059601776 | Control |
| 3381_454.7515 | 1.059528949 | Control |
| 1997_299.162 | 1.058832814 | Control |
| 3850_535.4017 | 1.058652315 | Control |
| 2927_383.1679 | 1.058542612 | Control |
| 1546_252.1343 | 1.058532069 | H2O2 |
| 3256_431.7293 | 1.058413199 | Control |
| 1621_259.1079 | 1.058253631 | Control |
| 2729_360.3626 | 1.057649134 | Control |
| 1728_272.1612 | 1.05733106 | H2O2 |
| 3239_426.358 | 1.057286804 | Control |
| 2920_382.1474 | 1.056765842 | Control |
| 194_130.1231 | 1.056725666 | H2O2 |
| 1335_232.1546 | 1.056377585 | Control |
| 1202_225.0658 | 1.056042922 | Control |
| 4040_600.4687 | 1.056038506 | H2O2 |
| 2687_356.3889 | 1.055854966 | Control |
| 679_182.9856 | 1.055119008 | Control |
| 2486_339.2397 | 1.054866097 | Control |
| 2362_329.3164 | 1.054423201 | Control |
| 3450_467.2069 | 1.053467593 | Control |
| 3989_569.6404 | 1.052703813 | Control |
| 2800_367.1746 | 1.052333482 | Control |
| 688_183.102 | 1.052070361 | Control |
| 3895_543.3418 | 1.051898543 | H2O2 |
| 3171_415.1914 | 1.051664778 | Control |
| 3395_457.2962 | 1.051644741 | Control |
| 1782_278.1601 | 1.051400928 | Control |
| 348_151.0967 | 1.051355627 | Control |
| 1312_230.248 | 1.051163119 | Control |
| 179_128.1073 | 1.049329138 | Control |
| 3999_574.3002 | 1.04908083 | Control |
| 1387_239.1177 | 1.04881478 | H2O2 |
| 1047_211.1443 | 1.048758707 | Control |
| 2105_306.6772 | 1.048627895 | Control |
| 3561_484.7537 | 1.046399703 | Control |
| 341_151.0355 | 1.046365737 | Control |
| 646_180.1023 | 1.046306321 | H2O2 |
| 3312_440.2679 | 1.045838319 | Control |
| 1136_218.1542 | 1.045588856 | H2O2 |
| 1838_285.1925 | 1.044989171 | H2O2 |
| 1272_228.1594 | 1.044733795 | Control |
| 1466_245.0922 | 1.044629656 | Control |
| 1393_239.1492 | 1.044093368 | Control |
| 2624_350.2726 | 1.044060545 | Control |
| 1322_231.1958 | 1.043286666 | Control |
| 2206_316.1942 | 1.043256433 | Control |
| 2604_348.238 | 1.043186118 | Control |
| 1943_293.1747 | 1.042975234 | Control |
| 494_165.0912 | 1.042955133 | Control |
| 2785_366.208 | 1.042562973 | Control |
| 3783_521.3283 | 1.042309156 | H2O2 |
| 2373_330.21 | 1.041862933 | Control |
| 1254_227.1545 | 1.041307067 | H2O2 |
| 659_181.0976 | 1.041264016 | H2O2 |
| 1801_280.1766 | 1.041157869 | H2O2 |
| 2915_380.2832 | 1.040859165 | Control |
| 2548_344.3161 | 1.04038414 | Control |
| 3778_520.273 | 1.039997879 | Control |
| 264_140.1186 | 1.039846844 | Control |
| 3771_518.3237 | 1.039744925 | Control |
| 1821_283.2004 | 1.038818831 | Control |
| 2517_343.1543 | 1.03862181 | Control |
| 167_126.1155 | 1.03857458 | Control |
| 1146_219.1132 | 1.038565094 | Control |
| 2483_339.2032 | 1.037753233 | H2O2 |
| 361_152.1185 | 1.037688909 | H2O2 |
| 1114_216.1709 | 1.037663409 | H2O2 |
| 1797_279.1593 | 1.03753003 | Control |
| 2945_385.1794 | 1.03748336 | Control |
| 1781_278.0675 | 1.03736866 | Control |
| 2141_311.1644 | 1.036821251 | Control |
| 485_164.1073 | 1.036150783 | H2O2 |
| 2088_306.1624 | 1.035912546 | Control |
| 3503_476.3071 | 1.035523773 | Control |
| 2882_377.3264 | 1.035174693 | Control |
| 2097_306.1915 | 1.035014197 | Control |
| 3487_472.7352 | 1.034787171 | Control |
| 1900_290.16 | 1.033996054 | Control |
| 2250_320.1642 | 1.033878908 | Control |
| 3773_518.3696 | 1.033830548 | Control |
| 1104_216.1234 | 1.033814761 | Control |
| 3827_529.3335 | 1.033749614 | H2O2 |
| 3095_405.2672 | 1.033430753 | Control |
| 1201_224.1759 | 1.032772912 | H2O2 |
| 2166_313.1801 | 1.032640912 | Control |
| 108_115.1232 | 1.03259622 | H2O2 |
| 562_172.076 | 1.032409299 | H2O2 |
| 3296_438.2982 | 1.032147754 | Control |
| 2046_302.2081 | 1.031726772 | Control |
| 1186_223.1331 | 1.031693887 | Control |
| 1242_227.1179 | 1.031566945 | H2O2 |
| 4256_800.3976 | 1.031190008 | Control |
| 543_170.0815 | 1.031058428 | H2O2 |
| 1400_239.1756 | 1.03092346 | Control |
| 833_195.1134 | 1.030314172 | Control |
| 3014_393.287 | 1.030246681 | Control |
| 2056_303.1544 | 1.029854627 | Control |
| 176_128.1073 | 1.029817353 | Control |
| 2294_325.1988 | 1.029719794 | Control |
| 3193_420.2595 | 1.029462875 | Control |
| 635_179.1069 | 1.029313906 | Control |
| 4002_575.2805 | 1.028969278 | Control |
| 2449_336.2022 | 1.026756123 | Control |
| 118_119.0607 | 1.026750084 | Control |
| 2458_336.2381 | 1.026282677 | Control |
| 4155_669.3358 | 1.026227036 | Control |
| 3124_408.2593 | 1.025600038 | Control |
| 2277_323.6714 | 1.025480571 | Control |
| 1287_229.1185 | 1.025275995 | H2O2 |
| 858_197.0926 | 1.024702459 | H2O2 |
| 675_182.1543 | 1.024292452 | H2O2 |
| 2389_331.2482 | 1.024161186 | Control |
| 1377_237.1486 | 1.024028148 | Control |
| 496_165.1025 | 1.023387828 | H2O2 |
| 1151_220.1083 | 1.023324564 | H2O2 |
| 3208_422.294 | 1.023191017 | Control |
| 1547_252.1343 | 1.022329928 | H2O2 |
| 670_181.1703 | 1.022218236 | H2O2 |
| 1143_218.2118 | 1.021571236 | Control |
| 605_176.1074 | 1.021156551 | H2O2 |
| 2050_302.2692 | 1.020150998 | Control |
| 2183_314.2325 | 1.019565826 | Control |
| 4274_841.4236 | 1.019538487 | Control |
| 2586_347.1928 | 1.019329539 | Control |
| 4012_581.803 | 1.019269787 | Control |
| 3131_408.3144 | 1.019244831 | Control |
| 2085_305.2229 | 1.019147567 | Control |
| 306_146.0716 | 1.018857975 | H2O2 |
| 284_142.123 | 1.018806105 | H2O2 |
| 1787_278.1609 | 1.017198267 | Control |
| 4166_676.4394 | 1.017067092 | Control |
| 3152_411.7281 | 1.017012879 | Control |
| 4200_697.6336 | 1.016743823 | Control |
| 105_115.0756 | 1.016608339 | Control |
| 2958_386.7381 | 1.015976532 | Control |
| 305_146.0604 | 1.015867514 | H2O2 |
| 760_189.1391 | 1.014779742 | H2O2 |
| 2992_392.2282 | 1.014014857 | Control |
| 2811_369.2133 | 1.013795144 | Control |
| 470_163.0755 | 1.013526317 | Control |
| 667_181.134 | 1.013006081 | Control |
| 1328_232.1338 | 1.012659658 | H2O2 |
| 92_113.9639 | 1.012370594 | Control |
| 125_122.0603 | 1.011781809 | H2O2 |
| 3918_548.7844 | 1.011633112 | Control |
| 2959_387.2003 | 1.011529783 | H2O2 |
| 1748_274.1543 | 1.011226322 | H2O2 |
| 3357_450.27 | 1.011012738 | H2O2 |
| 1470_245.1286 | 1.010386313 | H2O2 |
| 1726_272.1493 | 1.00989562 | Control |
| 2506_342.2141 | 1.00929118 | H2O2 |
| 2039_302.1603 | 1.00928216 | H2O2 |
| 4107_642.3863 | 1.008107435 | Control |
| 3459_468.3686 | 1.00785396 | Control |
| 3321_442.2301 | 1.007335568 | Control |
| 2736_362.1966 | 1.007265953 | Control |
| 4086_628.3851 | 1.005780214 | H2O2 |
| 2175_314.1817 | 1.005620341 | Control |
| 2786_366.212 | 1.004521854 | Control |
| 2361_329.2553 | 1.004360602 | Control |
| 1835_285.1562 | 1.004054144 | H2O2 |
| 482_163.133 | 1.003983321 | Control |
| 1736_272.2222 | 1.003872267 | Control |
| 2320_327.1966 | 1.00376021 | Control |
| 1868_287.2082 | 1.003508718 | H2O2 |
| 1681_267.1593 | 1.003347566 | H2O2 |
| 555_171.113 | 1.003132988 | H2O2 |
| 1390_239.128 | 1.002853899 | Control |
| 2721_360.1809 | 1.002681737 | Control |
| 851_196.1168 | 1.002511053 | Control |
| 51_102.1279 | 1.00164013 | Control |
| 1061_213.1026 | 1.001497946 | Control |
| 219_136.0761 | 1.00135859 | Control |
| 2590_347.2253 | 1.001093067 | Control |
| 4138_659.413 | 1.000728991 | Control |
| 2925_382.3167 | 1.00046478 | Control |
| 1571_255.1593 | 1.00046327 | Control |
| 2263_322.1863 | 1.000001399 | Control |
| 648_180.1023 | 1.955917817 | MPS |
| 490_165.0549 | 1.946869411 | MPS |
| 3204_422.2393 | 1.943768959 | MPS |
| 2472_338.2188 | 1.943749552 | MPS |
| 2788_366.2135 | 1.938538634 | MPS |
| 1906_290.1605 | 1.93547693 | MPS |
| 2114_308.1712 | 1.933263605 | MPS |
| 2198_315.181 | 1.917083279 | MPS |
| 3442_466.2649 | 1.889348101 | MPS |
| 3444_466.301 | 1.883220035 | Control |
| 2715_359.2069 | 1.881727487 | MPS |
| 3378_454.2652 | 1.868560355 | Control |
| 3141_410.2391 | 1.865517016 | MPS |
| 2024_301.1652 | 1.865279964 | MPS |
| 3379_454.2652 | 1.864007756 | MPS |
| 3048_399.1999 | 1.828563895 | MPS |
| 3144_410.2751 | 1.822654585 | MPS |
| 1463_244.2637 | 1.814458672 | MPS |
| 3044_398.2394 | 1.814330396 | MPS |
| 3445_466.3011 | 1.811425037 | MPS |
| 1567_255.123 | 1.808822071 | MPS |
| 2565_346.187 | 1.807134211 | MPS |
| 3359_450.306 | 1.802417847 | MPS |
| 1928_292.1763 | 1.80208025 | MPS |
| 1176_222.1493 | 1.802073609 | MPS |
| 38_102.1278 | 1.80201706 | MPS |
| 100_114.0916 | 1.799554751 | MPS |
| 145_124.0761 | 1.797606426 | MPS |
| 258_140.0823 | 1.79750827 | MPS |
| 301_145.1339 | 1.796625263 | MPS |
| 353_151.1233 | 1.795868276 | MPS |
| 471_163.0756 | 1.795518161 | MPS |
| 477_163.0867 | 1.794914145 | MPS |
| 504_165.1389 | 1.794912241 | MPS |
| 575_173.1077 | 1.792669471 | MPS |
| 580_173.1288 | 1.792148189 | MPS |
| 633_179.082 | 1.791918873 | MPS |
| 678_182.9855 | 1.790745364 | MPS |
| 683_183.0769 | 1.790093377 | MPS |
| 715_185.129 | 1.789494717 | MPS |
| 735_187.097 | 1.788137957 | MPS |
| 797_192.1388 | 1.787140279 | MPS |
| 898_200.1397 | 1.787122068 | MPS |
| 915_201.1967 | 1.786584325 | MPS |
| 916_202.0716 | 1.786542507 | MPS |
| 918_202.098 | 1.78563133 | MPS |
| 938_204.1234 | 1.785130723 | MPS |
| 983_208.1084 | 1.78507343 | MPS |
| 1004_209.1288 | 1.784284995 | MPS |
| 1244_227.128 | 1.781179162 | MPS |
| 1290_229.1186 | 1.7806653 | MPS |
| 1301_229.2276 | 1.780470695 | MPS |
| 1317_231.1495 | 1.777445706 | MPS |
| 1354_234.1854 | 1.77605511 | MPS |
| 1404_240.1704 | 1.775112818 | MPS |
| 1450_244.1077 | 1.77286044 | MPS |
| 1482_246.1701 | 1.766701773 | Control |
| 1494_247.1555 | 1.766120324 | MPS |
| 1561_254.1179 | 1.764545423 | MPS |
| 1564_254.1502 | 1.757884436 | MPS |
| 1565_255.1061 | 1.757593425 | MPS |
| 1592_256.181 | 1.752381496 | MPS |
| 1634_260.1753 | 1.752004936 | MPS |
| 1637_261.0906 | 1.749733398 | MPS |
| 1655_262.1657 | 1.747330735 | MPS |
| 1698_269.1849 | 1.73964495 | MPS |
| 1707_270.1455 | 1.739333315 | Control |
| 1724_272.1431 | 1.737602051 | MPS |
| 1766_276.1702 | 1.731341176 | MPS |
| 1767_276.1703 | 1.731259827 | MPS |
| 1769_276.181 | 1.729520405 | MPS |
| 1791_278.248 | 1.723621465 | MPS |
| 1800_280.1642 | 1.72359879 | MPS |
| 1946_294.1807 | 1.723464964 | MPS |
| 1990_298.2742 | 1.721895466 | MPS |
| 2008_299.2695 | 1.721350138 | MPS |
| 2009_299.2697 | 1.713783411 | MPS |
| 2035_301.2852 | 1.711974642 | Control |
| 2052_302.2886 | 1.711974642 | Control |
| 2087_306.1485 | 1.711974642 | Control |
| 2093_306.1915 | 1.711974642 | Control |
| 2276_323.2673 | 1.711974642 | Control |
| 2363_329.3165 | 1.711974642 | Control |
| 2436_335.1712 | 1.711974642 | Control |
| 2578_346.3318 | 1.711974642 | Control |
| 2674_356.1682 | 1.711974642 | Control |
| 2686_356.3638 | 1.711974642 | Control |
| 2779_365.1364 | 1.711974642 | Control |
| 2820_370.2751 | 1.711974642 | Control |
| 2857_374.3268 | 1.711974642 | Control |
| 2871_376.2334 | 1.711974642 | Control |
| 2890_378.2128 | 1.711974642 | Control |
| 2916_380.3315 | 1.711974642 | Control |
| 2957_386.2713 | 1.711974642 | Control |
| 2998_392.2297 | 1.711974642 | Control |
| 3057_400.1966 | 1.711974642 | Control |
| 3066_400.3786 | 1.711974642 | Control |
| 3072_402.2068 | 1.711974642 | Control |
| 3178_415.3209 | 1.711974642 | Control |
| 3268_433.3322 | 1.711974642 | Control |
| 3279_435.3795 | 1.711974642 | Control |
| 3318_441.3342 | 1.711974642 | Control |
| 3380_454.3167 | 1.711974642 | Control |
| 3411_460.2898 | 1.711974642 | Control |
| 3425_462.3534 | 1.711974642 | Control |
| 3433_463.4108 | 1.711974642 | Control |
| 3520_478.3236 | 1.711974642 | Control |
| 3594_489.4266 | 1.711974642 | Control |
| 3604_491.3742 | 1.711974642 | Control |
| 3688_506.2551 | 1.711974642 | Control |
| 3695_507.2087 | 1.711974642 | Control |
| 3714_511.2104 | 1.711974642 | Control |
| 3792_522.4377 | 1.711974642 | Control |
| 3847_534.2865 | 1.711974642 | Control |
| 3858_538.2813 | 1.711974642 | Control |
| 3914_546.7953 | 1.711974642 | Control |
| 4061_614.2725 | 1.711974642 | Control |
| 4120_650.3601 | 1.711974642 | Control |
| 4253_790.5892 | 1.711974642 | Control |
| 4292_929.8428 | 1.711974642 | Control |
| 2256_320.2076 | 1.711974642 | Control |
| 3424_462.3063 | 1.711974642 | Control |
| 1548_253.1053 | 1.711974642 | Control |
| 2652_353.1578 | 1.711974642 | Control |
| 2759_364.1979 | 1.711974642 | Control |
| 1995_299.1491 | 1.711974642 | Control |
| 2922_382.2442 | 1.711974642 | Control |
| 3090_404.2646 | 1.711974642 | Control |
| 3634_496.3063 | 1.711974642 | Control |
| 2498_341.1962 | 1.711974642 | Control |
| 1572_255.1594 | 1.711974642 | Control |
| 2138_311.1471 | 1.711974642 | Control |
| 3585_488.3582 | 1.711974642 | Control |
| 2230_318.1915 | 1.711974642 | Control |
| 1581_256.1335 | 1.711974642 | Control |
| 2923_382.2444 | 1.711974642 | Control |
| 4069_618.0053 | 1.711974642 | Control |
| 175_128.0709 | 1.711974642 | Control |
| 3644_497.3588 | 1.711974642 | Control |
| 3562_485.113 | 1.711974642 | Control |
| 202_132.0772 | 1.711974642 | Control |
| 1509_248.1495 | 1.711974642 | Control |
| 3701_508.1898 | 1.711974642 | Control |
| 3338_447.283 | 1.711974642 | Control |
| 3977_565.3535 | 1.711974642 | Control |
| 787_191.1645 | 1.711974642 | Control |
| 1123_217.1549 | 1.711974642 | Control |
| 418_158.0031 | 1.711974642 | Control |
| 4223_716.4373 | 1.711974642 | Control |
| 4037_598.4894 | 1.711974642 | Control |
| 2573_346.2227 | 1.711974642 | Control |
| 1271_228.1498 | 1.711974642 | Control |
| 1911_290.1965 | 1.711974642 | Control |
| 2840_372.243 | 1.711974642 | Control |
| 753_189.0986 | 1.711974642 | Control |
| 2665_355.174 | 1.711974642 | Control |
| 3426_462.3534 | 1.711974642 | Control |
| 1193_224.082 | 1.711974642 | Control |
| 4240_752.516 | 1.711974642 | Control |
| 3881_540.3335 | 1.711974642 | Control |
| 4234_740.5525 | 1.711974642 | Control |
| 1113_216.1596 | 1.711974642 | Control |
| 4017_584.3015 | 1.711974642 | Control |
| 101_115.0755 | 1.711974642 | Control |
| 4215_708.4897 | 1.711974642 | Control |
| 1878_288.1927 | 1.711974642 | Control |
| 1285_229.1084 | 1.711974642 | Control |
| 2371_330.1916 | 1.711974642 | Control |
| 3337_447.234 | 1.711974642 | Control |
| 4241_760.4634 | 1.711974642 | Control |
| 534_169.0862 | 1.711974642 | Control |
| 4144_664.4635 | 1.711974642 | Control |
| 2620_350.2176 | 1.711974642 | Control |
| 730_186.1856 | 1.711974642 | Control |
| 1927_292.1646 | 1.711974642 | Control |
| 1742_273.1595 | 1.711974642 | Control |
| 3206_422.2742 | 1.711974642 | Control |
| 506_165.1389 | 1.711974642 | Control |
| 313_148.076 | 1.711974642 | Control |
| 2669_355.2248 | 1.711974642 | Control |
| 571_173.0713 | 1.711974642 | Control |
| 1672_265.1297 | 1.711974642 | Control |
| 1250_227.1393 | 1.711974642 | Control |
| 3468_469.2961 | 1.711974642 | Control |
| 2972_389.2214 | 1.711974642 | Control |
| 1042_211.1237 | 1.711974642 | Control |
| 951_205.1338 | 1.711974642 | Control |
| 2152_312.1961 | 1.711974642 | Control |
| 3603_491.3091 | 1.711974642 | Control |
| 1261_227.1754 | 1.711974642 | Control |
| 567_172.1123 | 1.711974642 | Control |
| 443_159.9695 | 1.711974642 | Control |
| 2043_302.1965 | 1.711974642 | Control |
| 4024_587.3668 | 1.711974642 | Control |
| 1720_271.1804 | 1.711974642 | Control |
| 1826_284.1858 | 1.711974642 | Control |
| 2311_326.3055 | 1.711974642 | Control |
| 3997_573.3589 | 1.711974642 | Control |
| 3047_398.3631 | 1.711974642 | Control |
| 3842_532.3857 | 1.711974642 | Control |
| 2476_339.123 | 1.711974642 | Control |
| 193_130.123 | 1.711974642 | Control |
| 2270_323.147 | 1.711974642 | Control |
| 1132_218.1291 | 1.711974642 | Control |
| 4004_576.411 | 1.711974642 | Control |
| 864_197.1288 | 1.711974642 | Control |
| 2873_376.2336 | 1.711974642 | Control |
| 882_199.1236 | 1.711974642 | Control |
| 4261_804.4899 | 1.711974642 | Control |
| 869_197.1654 | 1.711974642 | Control |
| 1593_256.1811 | 1.711974642 | Control |
| 1306_230.1652 | 1.711974642 | Control |
| 956_206.0817 | 1.711974642 | Control |
| 2575_346.2227 | 1.711974642 | Control |
| 1144_219.1131 | 1.711974642 | Control |
| 969_207.1131 | 1.711974642 | Control |
| 424_158.1178 | 1.711974642 | Control |
| 2074_304.2848 | 1.711974642 | Control |
| 2546_344.2591 | 1.711974642 | Control |
| 3869_539.3701 | 1.706442283 | MPS |
| 2974_390.2127 | 1.703366642 | MPS |
| 1931_292.2123 | 1.701644028 | MPS |
| 4032_595.3722 | 1.700206034 | MPS |
| 1221_225.1851 | 1.699951995 | MPS |
| 3201_421.2326 | 1.695186552 | MPS |
| 2700_358.2266 | 1.691149865 | MPS |
| 1295_229.1439 | 1.687314653 | MPS |
| 3333_444.3323 | 1.684344582 | MPS |
| 2285_324.2436 | 1.682217293 | Control |
| 3071_402.1294 | 1.678374577 | MPS |
| 4072_620.4374 | 1.675418437 | MPS |
| 4280_848.5161 | 1.66973369 | MPS |
| 3974_564.2806 | 1.669095986 | MPS |
| 1875_288.1809 | 1.668363111 | MPS |
| 3024_394.2456 | 1.662016561 | MPS |
| 1196_224.1394 | 1.658135943 | Control |
| 436_159.092 | 1.650493681 | Control |
| 1816_283.1158 | 1.649635767 | Control |
| 303_146.0604 | 1.649406127 | MPS |
| 1916_290.1969 | 1.649239781 | MPS |
| 3112_407.3021 | 1.648808123 | MPS |
| 502_165.1137 | 1.648524494 | MPS |
| 375_153.1389 | 1.647858903 | MPS |
| 4151_667.415 | 1.64769696 | Control |
| 268_141.0913 | 1.640951746 | MPS |
| 2146_311.2082 | 1.639858865 | MPS |
| 2474_338.2902 | 1.634470471 | MPS |
| 4019_584.3589 | 1.633235725 | Control |
| 1771_276.1813 | 1.628709658 | MPS |
| 757_189.1027 | 1.627801059 | Control |
| 2870_376.2334 | 1.622636967 | MPS |
| 3543_482.4052 | 1.618808028 | MPS |
| 1877_288.181 | 1.612005373 | MPS |
| 505_165.1389 | 1.608543646 | MPS |
| 517_167.013 | 1.606556189 | Control |
| 3173_415.1947 | 1.604976733 | Control |
| 458_161.1076 | 1.599548138 | MPS |
| 3091_404.2646 | 1.596531913 | MPS |
| 1457_244.1908 | 1.595143433 | Control |
| 2107_307.0943 | 1.595023234 | MPS |
| 3913_546.7716 | 1.593156755 | MPS |
| 2396_332.2069 | 1.59006296 | MPS |
| 3554_484.2533 | 1.589908187 | MPS |
| 1521_249.1464 | 1.589836662 | MPS |
| 1537_251.1506 | 1.589479275 | MPS |
| 1_84.081 | 1.587879249 | Control |
| 4094_634.8434 | 1.587401142 | MPS |
| 751_188.1285 | 1.586364314 | MPS |
| 3738_513.3234 | 1.583452609 | MPS |
| 1084_214.217 | 1.582595778 | Control |
| 393_155.1069 | 1.576428208 | MPS |
| 1310_230.2116 | 1.575345915 | MPS |
| 4055_609.3798 | 1.574348515 | MPS |
| 1807_281.1739 | 1.571484098 | MPS |
| 3513_477.3583 | 1.571298558 | MPS |
| 2829_371.1631 | 1.570954694 | Control |
| 1228_226.1437 | 1.569753509 | Control |
| 215_135.1019 | 1.568878796 | Control |
| 2877_376.7248 | 1.568514246 | Control |
| 2171_313.2239 | 1.566266413 | MPS |
| 1260_227.1645 | 1.563349144 | MPS |
| 3054_399.3585 | 1.560285263 | MPS |
| 1336_232.1546 | 1.558664315 | MPS |
| 3197_420.7385 | 1.558587037 | Control |
| 3911_546.4012 | 1.556777727 | Control |
| 803_193.0977 | 1.555706425 | Control |
| 85_112.0759 | 1.555634362 | MPS |
| 2981_390.249 | 1.555251671 | MPS |
| 1533_250.178 | 1.554767338 | MPS |
| 2173_314.139 | 1.554430273 | MPS |
| 3195_420.2595 | 1.554030273 | Control |
| 119_119.0858 | 1.553839194 | Control |
| 1668_265.1048 | 1.551369593 | MPS |
| 684_183.0784 | 1.549950386 | MPS |
| 3902_544.3856 | 1.549590827 | MPS |
| 2645_352.2334 | 1.549098504 | Control |
| 157_125.9865 | 1.548868708 | MPS |
| 2341_328.2847 | 1.547968224 | MPS |
| 3056_400.1943 | 1.546723753 | Control |
| 34_101.0962 | 1.545922815 | Control |
| 967_207.0554 | 1.54582325 | MPS |
| 3257_431.7494 | 1.545500134 | Control |
| 132_123.0806 | 1.545008235 | Control |
| 3354_449.7597 | 1.54491793 | MPS |
| 4015_583.2801 | 1.544432906 | Control |
| 3185_417.3151 | 1.541532849 | Control |
| 136_123.0807 | 1.541315842 | Control |
| 2888_378.2126 | 1.539101108 | MPS |
| 4127_653.3572 | 1.538238363 | Control |
| 73_110.0603 | 1.537654276 | Control |
| 1170_222.1239 | 1.53738865 | Control |
| 3677_503.279 | 1.537306951 | MPS |
| 2595_348.2018 | 1.536684182 | Control |
| 2_84.081 | 1.535799793 | Control |
| 1576_255.1956 | 1.535230976 | Control |
| 3276_435.2336 | 1.534685831 | Control |
| 2540_344.2259 | 1.534584747 | Control |
| 3921_549.2902 | 1.533961945 | MPS |
| 2485_339.2084 | 1.532927863 | Control |
| 3151_411.7278 | 1.531215 | Control |
| 2909_380.2271 | 1.530675015 | MPS |
| 1945_293.2477 | 1.530086018 | Control |
| 3553_484.2408 | 1.52996493 | Control |
| 2032_301.2237 | 1.529903899 | MPS |
| 1650_262.1652 | 1.528947632 | Control |
| 1345_233.1749 | 1.527366421 | Control |
| 2781_365.2166 | 1.527026985 | MPS |
| 1627_260.1495 | 1.526976112 | Control |
| 2577_346.2953 | 1.526434567 | Control |
| 1378_237.1486 | 1.525685532 | Control |
| 2500_341.1976 | 1.525608627 | Control |
| 892_199.1559 | 1.525014586 | Control |
| 2111_307.194 | 1.524715286 | MPS |
| 2949_385.2392 | 1.5244981 | MPS |
| 4109_643.1366 | 1.524451184 | Control |
| 3996_573.3183 | 1.523867194 | Control |
| 634_179.1069 | 1.523861183 | MPS |
| 4113_645.3326 | 1.523515512 | Control |
| 355_151.1484 | 1.523256204 | Control |
| 1192_223.1809 | 1.523012885 | Control |
| 1057_212.2012 | 1.522731449 | MPS |
| 4021_584.4736 | 1.521879669 | MPS |
| 3944_556.2867 | 1.521337983 | Control |
| 3579_487.3068 | 1.521336835 | Control |
| 2391_332.1495 | 1.521148678 | MPS |
| 4068_617.3852 | 1.520371909 | Control |
| 3368_452.28 | 1.519835961 | Control |
| 2459_336.2389 | 1.51880237 | Control |
| 2753_363.2013 | 1.518511667 | MPS |
| 1461_244.2023 | 1.518190474 | MPS |
| 779_191.0859 | 1.517977351 | Control |
| 3448_466.3212 | 1.517709602 | Control |
| 4073_620.8426 | 1.517417225 | Control |
| 1150_219.1958 | 1.516724658 | Control |
| 2360_329.2187 | 1.516319124 | Control |
| 3757_516.3541 | 1.515679359 | MPS |
| 3143_410.2407 | 1.515666261 | Control |
| 2147_311.2559 | 1.515436583 | Control |
| 4202_699.3614 | 1.514883356 | MPS |
| 3874_540.2855 | 1.514411136 | Control |
| 4283_857.4218 | 1.513341976 | Control |
| 3247_428.7215 | 1.513107281 | Control |
| 4247_777.3863 | 1.512951616 | Control |
| 2948_385.2391 | 1.512666716 | Control |
| 2248_319.2303 | 1.510854384 | Control |
| 3094_405.2465 | 1.510821111 | Control |
| 2438_335.1884 | 1.510235701 | Control |
| 1798_279.1712 | 1.510071582 | MPS |
| 3061_400.2532 | 1.509873974 | Control |
| 3252_430.3893 | 1.50958601 | MPS |
| 1855_286.3106 | 1.509436 | Control |
| 1100_215.1644 | 1.509158309 | Control |
| 1775_277.1335 | 1.509032181 | Control |
| 3064_400.3062 | 1.508215371 | Control |
| 2440_335.2068 | 1.507919399 | Control |
| 2549_344.3161 | 1.507809816 | Control |
| 7_88.0759 | 1.507148681 | Control |
| 4046_604.3137 | 1.507141202 | Control |
| 70_110.0089 | 1.506792118 | MPS |
| 680_182.9856 | 1.505786078 | Control |
| 1014_209.1541 | 1.505570458 | Control |
| 3099_406.226 | 1.504663443 | Control |
| 1917_290.1969 | 1.504491261 | Control |
| 810_193.144 | 1.504460043 | Control |
| 2426_334.2227 | 1.50444628 | Control |
| 676_182.1544 | 1.503287653 | Control |
| 324_149.06 | 1.503081691 | MPS |
| 2374_330.21 | 1.502841672 | Control |
| 2439_335.2059 | 1.502287773 | Control |
| 2323_327.2031 | 1.501860028 | Control |
| 4103_640.8253 | 1.501664518 | Control |
| 1616_258.218 | 1.500808604 | Control |
| 2411_333.1901 | 1.500463482 | Control |
| 703_184.0973 | 1.500309936 | MPS |
| 1870_287.2081 | 1.500026025 | MPS |
| 217_136.0735 | 1.498194776 | Control |
| 1266_228.1265 | 1.497725835 | Control |
| 4228_725.8611 | 1.497677193 | Control |
| 812_193.1704 | 1.496941125 | MPS |
| 2424_334.2226 | 1.496220369 | Control |
| 1812_282.1821 | 1.496099764 | Control |
| 1198_224.1396 | 1.496040821 | Control |
| 1414_241.1437 | 1.495786071 | Control |
| 13_90.526 | 1.495462479 | Control |
| 569_172.1698 | 1.494994568 | Control |
| 2643_352.2335 | 1.494962971 | Control |
| 3405_459.218 | 1.494144812 | MPS |
| 1983_297.2426 | 1.493912797 | Control |
| 1841_285.2541 | 1.493100409 | Control |
| 3221_424.2546 | 1.492950474 | Control |
| 977_207.1498 | 1.492177529 | MPS |
| 4081_626.3349 | 1.491651472 | Control |
| 542_170.0814 | 1.491185432 | MPS |
| 2271_323.1473 | 1.491159577 | Control |
| 1489_246.2429 | 1.491099789 | Control |
| 2634_352.1483 | 1.490925209 | Control |
| 4053_608.3067 | 1.489991724 | Control |
| 3781_520.3559 | 1.489715992 | Control |
| 59_107.0704 | 1.489481037 | MPS |
| 4257_800.3998 | 1.489099025 | Control |
| 1883_288.2898 | 1.48891828 | Control |
| 3070_401.2343 | 1.488717588 | Control |
| 106_115.0756 | 1.488442342 | Control |
| 3196_420.724 | 1.487700167 | Control |
| 3148_410.3479 | 1.487582578 | Control |
| 3176_415.2494 | 1.487254042 | Control |
| 2622_350.2178 | 1.487166194 | Control |
| 2955_386.2417 | 1.487112293 | Control |
| 3016_394.2082 | 1.486806026 | MPS |
| 2662_355.1071 | 1.486725306 | Control |
| 1954_294.2067 | 1.486134308 | Control |
| 1915_290.1968 | 1.485712171 | Control |
| 3271_434.2382 | 1.484896121 | Control |
| 3833_530.3101 | 1.484059692 | Control |
| 4085_628.3128 | 1.483770073 | Control |
| 159_125.9866 | 1.48348004 | Control |
| 3163_413.2763 | 1.483322557 | Control |
| 3376_453.763 | 1.483247794 | MPS |
| 1856_286.6695 | 1.482206677 | Control |
| 162_126.0917 | 1.481736857 | Control |
| 3297_438.325 | 1.481681057 | Control |
| 1575_255.1857 | 1.481629394 | Control |
| 3298_438.3254 | 1.481553339 | Control |
| 1430_242.1751 | 1.480911089 | Control |
| 4132_656.4009 | 1.480703743 | MPS |
| 2683_356.2797 | 1.480570218 | Control |
| 1958_295.1523 | 1.48043227 | Control |
| 4248_782.3938 | 1.480027056 | Control |
| 642_179.1546 | 1.479939594 | Control |
| 1330_232.1446 | 1.479850524 | Control |
| 431_158.9642 | 1.479574911 | Control |
| 2324_327.2396 | 1.479004134 | MPS |
| 1133_218.1389 | 1.478898025 | MPS |
| 2529_343.2346 | 1.47848185 | Control |
| 1729_272.1857 | 1.477642969 | Control |
| 2505_342.2052 | 1.477476005 | Control |
| 1142_218.2117 | 1.477191234 | MPS |
| 2016_300.217 | 1.476325413 | Control |
| 654_180.1597 | 1.475717744 | MPS |
| 4203_700.4271 | 1.475622476 | Control |
| 3351_449.2349 | 1.474762313 | Control |
| 1822_284.1493 | 1.474508718 | Control |
| 1256_227.1643 | 1.474128628 | Control |
| 1746_273.2539 | 1.473900606 | Control |
| 4161_672.5262 | 1.473626356 | Control |
| 856_196.1701 | 1.473592791 | Control |
| 1985_298.165 | 1.472778242 | Control |
| 3008_392.3193 | 1.472758837 | MPS |
| 3608_492.3242 | 1.472748214 | Control |
| 3038_397.1839 | 1.472385149 | Control |
| 1515_249.0526 | 1.472319814 | Control |
| 2651_353.0788 | 1.472050379 | Control |
| 1757_275.0891 | 1.471864489 | Control |
| 3287_436.3095 | 1.47157673 | Control |
| 2846_373.2273 | 1.471239119 | Control |
| 4222_716.3317 | 1.470538748 | Control |
| 1008_209.1539 | 1.470282689 | Control |
| 4125_652.8404 | 1.469851141 | Control |
| 2134_310.2742 | 1.46964811 | Control |
| 46_102.1279 | 1.469211233 | Control |
| 1002_209.1176 | 1.469161599 | Control |
| 278_141.959 | 1.468191783 | Control |
| 98_114.0916 | 1.468039214 | Control |
| 835_195.1231 | 1.46765136 | Control |
| 2340_328.2485 | 1.467547037 | Control |
| 2539_344.2256 | 1.467222182 | Control |
| 3104_406.2798 | 1.46683255 | Control |
| 1474_245.1861 | 1.46639078 | MPS |
| 4272_840.4159 | 1.466246533 | Control |
| 2881_377.3165 | 1.465799928 | Control |
| 2673_355.2958 | 1.465327553 | Control |
| 1617_258.2429 | 1.464793728 | Control |
| 3875_540.2921 | 1.464288271 | Control |
| 1092_215.1181 | 1.464225581 | Control |
| 4285_868.4305 | 1.464208337 | Control |
| 3130_408.3136 | 1.463861635 | Control |
| 3269_433.3349 | 1.463222984 | Control |
| 1682_267.1592 | 1.463039881 | MPS |
| 3961_559.297 | 1.462895069 | MPS |
| 3612_492.7661 | 1.46258276 | Control |
| 1697_269.1749 | 1.461837435 | MPS |
| 2158_313.1007 | 1.461682753 | Control |
| 3542_482.3607 | 1.461682694 | MPS |
| 4232_738.3707 | 1.461603317 | Control |
| 2633_351.2009 | 1.461377333 | Control |
| 1594_256.1904 | 1.461127275 | Control |
| 3970_563.2861 | 1.460894295 | Control |
| 2971_389.1885 | 1.460836343 | Control |
| 1102_216.1232 | 1.460832492 | Control |
| 4190_691.3497 | 1.460741568 | Control |
| 3718_511.2786 | 1.458927776 | Control |
| 4252_789.8919 | 1.458505502 | Control |
| 3942_556.1748 | 1.458028626 | Control |
| 1303_230.129 | 1.456901506 | Control |
| 2113_308.1477 | 1.456814857 | Control |
| 2089_306.1914 | 1.456309946 | Control |
| 2589_347.2178 | 1.456084622 | Control |
| 4260_803.3997 | 1.456067462 | MPS |
| 2394_332.1498 | 1.456040176 | MPS |
| 2348_329.1574 | 1.455669946 | Control |
| 817_194.1181 | 1.453360427 | Control |
| 3434_463.7756 | 1.453139416 | MPS |
| 837_195.1383 | 1.452622935 | Control |
| 1524_249.2062 | 1.452012649 | Control |
| 3987_568.7956 | 1.451981626 | Control |
| 1658_263.0816 | 1.451844507 | Control |
| 4130_654.3321 | 1.451770379 | MPS |
| 4092_634.3434 | 1.451278993 | Control |
| 3012_393.2671 | 1.451115708 | MPS |
| 1952_294.1925 | 1.451106481 | Control |
| 1491_247.1232 | 1.4510654 | Control |
| 3713_510.3832 | 1.451010705 | MPS |
| 4087_628.4998 | 1.450908024 | Control |
| 31_101.0598 | 1.450590025 | Control |
| 2832_371.2116 | 1.449859379 | Control |
| 1894_289.2484 | 1.44964392 | Control |
| 3576_486.2931 | 1.44955507 | Control |
| 1704_270.1337 | 1.449516105 | MPS |
| 1436_242.2844 | 1.449366311 | Control |
| 1337_232.1547 | 1.448532585 | Control |
| 2417_334.1865 | 1.448495068 | Control |
| 2668_355.2035 | 1.447809035 | Control |
| 2928_383.2043 | 1.447736178 | Control |
| 3161_413.2337 | 1.447066737 | MPS |
| 2150_312.1557 | 1.446873144 | Control |
| 1885_289.1048 | 1.445983384 | Control |
| 512_166.0866 | 1.445935095 | MPS |
| 4269_829.3912 | 1.445705572 | MPS |
| 2286_324.2899 | 1.445342293 | Control |
| 1739_272.2586 | 1.445304508 | Control |
| 2997_392.2284 | 1.445155452 | Control |
| 3128_408.2796 | 1.445015966 | Control |
| 1452_244.1543 | 1.444948802 | Control |
| 3102_406.244 | 1.444495201 | MPS |
| 3871_539.7887 | 1.444371212 | Control |
| 4198_696.8666 | 1.444343419 | Control |
| 1980_297.2407 | 1.44419915 | Control |
| 3069_401.1788 | 1.443912187 | MPS |
| 2188_315.1227 | 1.443744175 | Control |
| 188_130.0655 | 1.443504724 | MPS |
| 3611_492.7657 | 1.443250701 | Control |
| 3187_418.2795 | 1.443241925 | Control |
| 1809_282.1573 | 1.443212044 | Control |
| 362_152.1284 | 1.443075796 | Control |
| 1765_275.2777 | 1.442689632 | Control |
| 138_123.0807 | 1.442119989 | Control |
| 1851_286.2014 | 1.441699639 | Control |
| 1908_290.1964 | 1.44117463 | Control |
| 2296_325.2012 | 1.441042314 | Control |
| 2065_304.1756 | 1.440918128 | Control |
| 4210_707.283 | 1.440796421 | Control |
| 2184_314.2691 | 1.440743671 | Control |
| 1788_278.1666 | 1.440253412 | Control |
| 2252_320.2071 | 1.440010128 | Control |
| 3336_446.2819 | 1.439088519 | Control |
| 4249_784.5787 | 1.43866052 | Control |
| 1459_244.1909 | 1.437784034 | Control |
| 3722_511.7769 | 1.437375755 | Control |
| 1955_294.2641 | 1.43722514 | MPS |
| 2883_377.7148 | 1.437063619 | Control |
| 3976_564.3588 | 1.436914716 | Control |
| 731_186.222 | 1.436899509 | Control |
| 3403_458.3324 | 1.436822843 | Control |
| 1699_269.2093 | 1.436191187 | Control |
| 3007_392.2654 | 1.435918305 | Control |
| 406_156.1022 | 1.43570686 | Control |
| 3892_542.9682 | 1.434435868 | Control |
| 417_157.0974 | 1.434399448 | Control |
| 3601_491.243 | 1.434295845 | Control |
| 2970_389.1122 | 1.43421595 | MPS |
| 3394_457.2319 | 1.434168161 | MPS |
| 3935_553.2947 | 1.434019053 | Control |
| 57_105.0338 | 1.433894068 | Control |
| 1776_277.1337 | 1.433416922 | Control |
| 2112_307.1942 | 1.433254546 | MPS |
| 1654_262.1656 | 1.433197128 | Control |
| 522_167.1182 | 1.43313043 | Control |
| 262_140.1074 | 1.433060807 | Control |
| 518_167.0131 | 1.433029615 | Control |
| 2151_312.1803 | 1.432856484 | Control |
| 1477_246.1338 | 1.43273482 | Control |
| 89_113.0599 | 1.43264175 | Control |
| 212_135.1019 | 1.432185921 | Control |
| 1786_278.1605 | 1.431404685 | Control |
| 1989_298.2168 | 1.431259374 | Control |
| 1481_246.1698 | 1.431244007 | Control |
| 95_114.0916 | 1.43123699 | Control |
| 1101_216.0479 | 1.430899534 | Control |
| 4059_612.3746 | 1.429949399 | Control |
| 1819_283.1757 | 1.429925949 | MPS |
| 2001_299.1857 | 1.429802011 | MPS |
| 3098_406.2258 | 1.42929999 | Control |
| 299_145.0764 | 1.429270304 | Control |
| 946_204.1599 | 1.429058341 | Control |
| 3220_424.2545 | 1.428620537 | Control |
| 3118_408.2237 | 1.428313518 | Control |
| 2596_348.2021 | 1.427886357 | Control |
| 1060_213.1025 | 1.427653478 | Control |
| 4288_892.5425 | 1.427370873 | MPS |
| 1620_259.0942 | 1.426464 | Control |
| 2569_346.2226 | 1.426352425 | Control |
| 4165_676.3406 | 1.425987735 | Control |
| 78_111.0807 | 1.425980389 | Control |
| 257_140.0711 | 1.425896425 | Control |
| 1111_216.1498 | 1.425783792 | Control |
| 4149_666.4689 | 1.425490748 | Control |
| 834_195.1231 | 1.424898512 | Control |
| 2054_302.3056 | 1.424481057 | Control |
| 1912_290.1965 | 1.424260967 | Control |
| 380_154.0866 | 1.42373667 | Control |
| 2571_346.2227 | 1.423265733 | Control |
| 2495_341.1578 | 1.42326343 | Control |
| 4254_793.3943 | 1.423130434 | Control |
| 2100_306.1918 | 1.422932466 | Control |
| 1376_237.1485 | 1.422337101 | MPS |
| 2342_328.2847 | 1.422207436 | Control |
| 2515_343.1543 | 1.422099072 | Control |
| 3245_427.3896 | 1.422083887 | MPS |
| 2255_320.2075 | 1.42194979 | Control |
| 981_208.0794 | 1.421889981 | Control |
| 1873_288.1808 | 1.421723333 | Control |
| 2587_347.2061 | 1.42147865 | Control |
| 55_105.0337 | 1.42107229 | Control |
| 2246_319.1865 | 1.421032774 | Control |
| 2647_352.2335 | 1.420793987 | Control |
| 1607_257.2112 | 1.420333654 | Control |
| 3003_392.2648 | 1.420280335 | Control |
| 1794_279.0936 | 1.420142824 | Control |
| 4275_842.4181 | 1.420044729 | MPS |
| 2090_306.1914 | 1.419906608 | MPS |
| 47_102.1279 | 1.41981719 | Control |
| 3125_408.2594 | 1.419578233 | Control |
| 2644_352.2334 | 1.419567691 | Control |
| 1817_283.1405 | 1.419114041 | Control |
| 8_89.0598 | 1.418481628 | Control |
| 1419_241.18 | 1.418308121 | Control |
| 2413_333.2768 | 1.41788654 | Control |
| 4031_594.4946 | 1.417752211 | MPS |
| 3652_499.2301 | 1.417708616 | Control |
| 2238_318.2277 | 1.417330256 | Control |
| 466_162.9071 | 1.417207809 | Control |
| 3501_476.2988 | 1.416457612 | Control |
| 2441_335.2204 | 1.416278165 | Control |
| 556_171.1494 | 1.41603415 | Control |
| 752_188.1285 | 1.416027724 | MPS |
| 559_171.1858 | 1.415690964 | Control |
| 1749_274.165 | 1.415621897 | Control |
| 994_208.1911 | 1.415494917 | Control |
| 128_122.0967 | 1.414722997 | Control |
| 1674_265.1434 | 1.414457051 | Control |
| 3656_499.3146 | 1.413757553 | Control |
| 2918_381.1103 | 1.413726897 | MPS |
| 1542_251.1757 | 1.413275348 | Control |
| 619_177.1027 | 1.412863462 | Control |
| 2516_343.1543 | 1.412720566 | Control |
| 1737_272.2222 | 1.412555078 | MPS |
| 3011_393.2504 | 1.41245458 | Control |
| 690_183.102 | 1.41232523 | Control |
| 2007_299.2583 | 1.412052596 | Control |
| 77_111.0807 | 1.412026891 | Control |
| 2772_364.2337 | 1.411772238 | Control |
| 719_185.1653 | 1.411687667 | Control |
| 2646_352.2334 | 1.411539506 | Control |
| 885_199.1333 | 1.411010775 | MPS |
| 1363_236.1131 | 1.410958815 | Control |
| 2216_317.0474 | 1.410942112 | MPS |
| 1934_293.1133 | 1.410853319 | Control |
| 1608_258.1235 | 1.41033654 | MPS |
| 223_136.1125 | 1.410058578 | Control |
| 2103_306.3005 | 1.409185712 | Control |
| 1735_272.2221 | 1.408676699 | MPS |
| 412_157.0862 | 1.408638882 | Control |
| 2834_371.3272 | 1.407441849 | Control |
| 463_162.0916 | 1.407269536 | Control |
| 53_105.0335 | 1.406873024 | Control |
| 1700_269.2114 | 1.406823959 | MPS |
| 756_189.1027 | 1.406618886 | Control |
| 1248_227.1281 | 1.406515287 | Control |
| 1866_287.2006 | 1.405992785 | Control |
| 88_113.0598 | 1.405781841 | Control |
| 379_154.0866 | 1.405264895 | Control |
| 131_123.0805 | 1.40517164 | MPS |
| 2149_311.2947 | 1.404440678 | Control |
| 1987_298.1917 | 1.404135483 | Control |
| 2465_337.2603 | 1.403818094 | MPS |
| 2041_302.1965 | 1.403804198 | MPS |
| 1623_259.1906 | 1.403416985 | MPS |
| 1913_290.1965 | 1.402546032 | MPS |
| 1484_246.1703 | 1.402201246 | Control |
| 253_139.1234 | 1.401969168 | Control |
| 4250_785.3919 | 1.401819907 | MPS |
| 4006_579.1573 | 1.401773266 | MPS |
| 3004_392.2648 | 1.401574994 | Control |
| 4236_744.4534 | 1.40058411 | Control |
| 511_166.0866 | 1.400058014 | Control |
| 2742_362.2176 | 1.399556859 | Control |
| 4090_631.393 | 1.399440951 | Control |
| 276_141.9588 | 1.399310189 | Control |
| 962_206.1388 | 1.399299034 | Control |
| 1204_225.1025 | 1.399102512 | Control |
| 2545_344.2434 | 1.398712084 | Control |
| 2938_384.2611 | 1.398708918 | Control |
| 3084_403.3692 | 1.397195795 | Control |
| 2869_376.2334 | 1.396994794 | Control |
| 663_181.1339 | 1.39681725 | Control |
| 2504_342.196 | 1.396729856 | Control |
| 3882_540.4484 | 1.396582618 | Control |
| 1009_209.154 | 1.396367827 | Control |
| 2905_380.1934 | 1.396039976 | Control |
| 11_90.526 | 1.395946501 | Control |
| 3391_455.3457 | 1.395767133 | Control |
| 388_155.0818 | 1.39517382 | Control |
| 3288_436.3998 | 1.39488511 | Control |
| 226_137.0714 | 1.394724333 | Control |
| 2726_360.2022 | 1.394523855 | Control |
| 3398_458.2289 | 1.394515125 | Control |
| 3849_535.3366 | 1.393716484 | Control |
| 139_123.0807 | 1.393647759 | Control |
| 535_169.0862 | 1.393617624 | Control |
| 1785_278.1604 | 1.393552575 | Control |
| 2027_301.1801 | 1.393420925 | MPS |
| 65_108.081 | 1.393321443 | Control |
| 1286_229.1185 | 1.39291117 | Control |
| 369_153.1025 | 1.392540262 | Control |
| 2312_326.3056 | 1.392324222 | Control |
| 4063_614.4843 | 1.392085463 | Control |
| 3404_458.3479 | 1.391787623 | Control |
| 3092_404.2647 | 1.391666742 | Control |
| 4038_599.4271 | 1.391555823 | MPS |
| 4175_681.355 | 1.390848741 | Control |
| 660_181.0976 | 1.390837813 | Control |
| 2106_306.6772 | 1.390759029 | Control |
| 2336_328.1986 | 1.390586927 | Control |
| 3116_408.222 | 1.390356721 | MPS |
| 1690_268.1544 | 1.390288004 | Control |
| 737_187.1234 | 1.390248228 | Control |
| 2264_322.1864 | 1.39010017 | Control |
| 1712_270.1854 | 1.389705396 | Control |
| 2034_301.2851 | 1.389594396 | Control |
| 2000_299.1752 | 1.389560999 | Control |
| 86_112.0871 | 1.389372452 | Control |
| 1180_222.1716 | 1.389111947 | Control |
| 3873_540.2837 | 1.38878429 | MPS |
| 515_166.1229 | 1.388774751 | Control |
| 1733_272.201 | 1.388773017 | MPS |
| 67_109.1014 | 1.388553147 | Control |
| 1334_232.1546 | 1.388467467 | Control |
| 1925_291.2319 | 1.38807994 | Control |
| 3153_412.1581 | 1.387399909 | Control |
| 3525_480.1576 | 1.387056285 | Control |
| 1288_229.1186 | 1.386810773 | Control |
| 748_188.1077 | 1.386030326 | Control |
| 4101_639.3982 | 1.385948516 | Control |
| 536_169.0862 | 1.38560434 | Control |
| 903_200.2014 | 1.385007068 | Control |
| 1806_281.1641 | 1.384842677 | Control |
| 588_174.1031 | 1.384714676 | MPS |
| 3192_420.2594 | 1.38355537 | Control |
| 1294_229.1438 | 1.383223257 | MPS |
| 3123_408.2588 | 1.383159224 | Control |
| 2297_325.2131 | 1.383081806 | MPS |
| 365_153.0913 | 1.383059145 | Control |
| 941_204.1235 | 1.382854929 | Control |
| 1630_260.1497 | 1.38281712 | Control |
| 2225_318.1914 | 1.382813873 | Control |
| 208_134.0968 | 1.38268816 | Control |
| 2042_302.1965 | 1.382663345 | MPS |
| 639_179.1183 | 1.382391297 | Control |
| 1022_210.1241 | 1.382386298 | Control |
| 771_190.1231 | 1.382332396 | Control |
| 1570_255.1592 | 1.381800059 | Control |
| 1829_284.2073 | 1.381351801 | Control |
| 1464_245.0786 | 1.381259657 | MPS |
| 1189_223.1695 | 1.380714387 | Control |
| 1583_256.1543 | 1.379884012 | Control |
| 2911_380.2284 | 1.379825255 | Control |
| 2345_328.3213 | 1.379722512 | Control |
| 3356_450.2697 | 1.379408759 | Control |
| 3158_413.1786 | 1.379207622 | Control |
| 3229_425.2149 | 1.379197088 | Control |
| 3238_426.2701 | 1.379012728 | Control |
| 1510_248.1496 | 1.378900454 | Control |
| 199_131.1183 | 1.378751193 | Control |
| 3704_508.3126 | 1.378685381 | Control |
| 1619_258.2793 | 1.378668805 | Control |
| 2070_304.1759 | 1.378444508 | Control |
| 829_195.102 | 1.378301932 | Control |
| 1896_289.2739 | 1.378182481 | Control |
| 1353_234.1603 | 1.378099005 | Control |
| 4294_952.5794 | 1.377277517 | Control |
| 744_188.071 | 1.377069749 | Control |
| 296_144.1386 | 1.376480333 | MPS |
| 445_160.0759 | 1.376405343 | Control |
| 1311_230.248 | 1.37635868 | Control |
| 3083_403.2327 | 1.374680812 | Control |
| 4251_788.4797 | 1.374569473 | Control |
| 949_205.0976 | 1.374248616 | Control |
| 4176_682.3185 | 1.374136384 | MPS |
| 2337_328.2122 | 1.373244502 | Control |
| 1541_251.1642 | 1.373093996 | MPS |
| 18_96.0445 | 1.373013457 | Control |
| 2002_299.2007 | 1.372980189 | Control |
| 2813_369.2135 | 1.372919739 | Control |
| 29_100.0758 | 1.372774855 | Control |
| 1747_274.154 | 1.372049173 | Control |
| 2209_316.2121 | 1.371939046 | Control |
| 4139_661.4113 | 1.371000251 | Control |
| 3518_478.3013 | 1.370568068 | MPS |
| 1062_213.1125 | 1.370373879 | Control |
| 2605_348.2382 | 1.370237939 | Control |
| 749_188.1188 | 1.3698236 | Control |
| 2375_330.2277 | 1.369421869 | Control |
| 875_199.097 | 1.369326094 | Control |
| 4290_908.5531 | 1.368365724 | Control |
| 976_207.1497 | 1.367750951 | Control |
| 793_192.1135 | 1.367467952 | Control |
| 32_101.0599 | 1.366972768 | Control |
| 1276_228.1708 | 1.366971457 | Control |
| 1023_210.1241 | 1.366894605 | Control |
| 1447_243.1594 | 1.366615631 | Control |
| 2682_356.2297 | 1.366014105 | Control |
| 1085_214.2532 | 1.36578039 | Control |
| 1839_285.1926 | 1.36559429 | Control |
| 1525_250.1185 | 1.365367951 | Control |
| 4293_936.5689 | 1.365064508 | Control |
| 394_155.1069 | 1.364236592 | MPS |
| 750_188.1188 | 1.364082048 | Control |
| 1696_269.175 | 1.363886768 | Control |
| 408_156.1134 | 1.363861465 | Control |
| 3986_568.3484 | 1.363393949 | Control |
| 427_158.1541 | 1.363305472 | Control |
| 1601_257.161 | 1.362855215 | Control |
| 1197_224.1395 | 1.362560618 | Control |
| 3036_396.3324 | 1.362308859 | Control |
| 2153_312.1961 | 1.362264964 | Control |
| 3375_453.247 | 1.362141999 | Control |
| 320_149.0236 | 1.362108459 | Control |
| 1055_212.1649 | 1.361725278 | Control |
| 1639_261.112 | 1.361531743 | Control |
| 766_190.098 | 1.361306258 | MPS |
| 647_180.1023 | 1.361255929 | Control |
| 2131_310.1805 | 1.361244078 | Control |
| 3700_507.3205 | 1.36106472 | Control |
| 612_177.0549 | 1.360738024 | Control |
| 1823_284.1757 | 1.360179241 | Control |
| 3632_496.2883 | 1.360091973 | Control |
| 60_107.0704 | 1.35987311 | Control |
| 610_176.1187 | 1.359686902 | Control |
| 66_109.0762 | 1.359644213 | Control |
| 1540_251.1642 | 1.359546175 | Control |
| 699_183.1384 | 1.359500819 | Control |
| 729_186.1129 | 1.359240398 | Control |
| 64_108.081 | 1.358875721 | Control |
| 1037_211.1081 | 1.358638549 | Control |
| 1947_294.1919 | 1.358502944 | Control |
| 2641_352.2333 | 1.358076518 | Control |
| 2072_304.2123 | 1.357841495 | MPS |
| 4284_864.5265 | 1.357299235 | Control |
| 591_175.0869 | 1.356689585 | Control |
| 1168_221.6426 | 1.356281058 | Control |
| 189_130.0866 | 1.355380778 | Control |
| 1598_257.1278 | 1.354994256 | MPS |
| 2192_315.123 | 1.354625259 | Control |
| 1590_256.176 | 1.354032513 | Control |
| 711_185.1176 | 1.353809529 | Control |
| 3470_469.7097 | 1.353733467 | Control |
| 144_124.0761 | 1.353578595 | Control |
| 26_100.0506 | 1.35312875 | MPS |
| 1024_210.1241 | 1.352867948 | Control |
| 1978_297.1926 | 1.351992962 | Control |
| 1360_235.1808 | 1.351662622 | Control |
| 561_172.0759 | 1.351588582 | Control |
| 1413_241.1438 | 1.3510314 | Control |
| 2004_299.208 | 1.350265386 | MPS |
| 197_130.9668 | 1.349415605 | Control |
| 1181_223.0966 | 1.349359091 | Control |
| 3172_415.1945 | 1.349256566 | Control |
| 62_108.0446 | 1.34842768 | Control |
| 1606_257.1754 | 1.3482329 | Control |
| 1490_247.1079 | 1.34819776 | Control |
| 1969_297.132 | 1.348092891 | Control |
| 2397_332.207 | 1.347342115 | Control |
| 302_145.1339 | 1.346071701 | Control |
| 4128_653.4061 | 1.346059561 | Control |
| 2598_348.2023 | 1.345767205 | Control |
| 3142_410.2396 | 1.345701713 | Control |
| 2067_304.1757 | 1.345664191 | MPS |
| 1183_223.108 | 1.345547455 | Control |
| 1267_228.1342 | 1.345444672 | MPS |
| 2574_346.2227 | 1.345348604 | Control |
| 4164_675.4192 | 1.345303689 | Control |
| 706_184.17 | 1.34420551 | MPS |
| 726_186.0917 | 1.34418109 | Control |
| 696_183.1133 | 1.343402845 | Control |
| 3798_524.3232 | 1.343089111 | Control |
| 2767_364.2334 | 1.342918094 | Control |
| 1965_296.1607 | 1.342733223 | Control |
| 600_176.071 | 1.34222398 | Control |
| 56_105.0337 | 1.341841255 | Control |
| 539_169.1225 | 1.341096007 | Control |
| 2418_334.1866 | 1.341066156 | Control |
| 243_138.0918 | 1.340955714 | Control |
| 4265_820.5002 | 1.340606887 | Control |
| 762_190.0868 | 1.34033283 | Control |
| 549_170.1541 | 1.339885542 | Control |
| 557_171.1494 | 1.339724207 | Control |
| 568_172.1335 | 1.338660297 | Control |
| 1233_226.1552 | 1.338481399 | MPS |
| 283_142.0866 | 1.337746698 | Control |
| 821_194.1544 | 1.337549128 | Control |
| 3389_455.2624 | 1.337102284 | MPS |
| 1602_257.165 | 1.336567452 | Control |
| 2990_392.193 | 1.335947791 | Control |
| 450_160.0872 | 1.335594039 | Control |
| 1314_231.1131 | 1.335593909 | Control |
| 2821_370.2954 | 1.335561855 | MPS |
| 917_202.0869 | 1.335425935 | Control |
| 1178_222.1602 | 1.335288565 | Control |
| 1361_235.1808 | 1.335235633 | Control |
| 350_151.1233 | 1.334023506 | Control |
| 560_172.0759 | 1.333109334 | MPS |
| 1811_282.1815 | 1.333032506 | Control |
| 1275_228.1709 | 1.332840101 | Control |
| 2288_325.1437 | 1.332610913 | Control |
| 551_171.0921 | 1.332272783 | Control |
| 1327_232.1292 | 1.332143155 | Control |
| 191_130.0867 | 1.332029505 | Control |
| 1243_227.118 | 1.331988689 | Control |
| 516_166.123 | 1.331819953 | Control |
| 714_185.1289 | 1.331155614 | Control |
| 1298_229.155 | 1.331099863 | Control |
| 1411_241.1334 | 1.330763402 | MPS |
| 1568_255.1489 | 1.330571134 | MPS |
| 2851_374.2176 | 1.33022356 | MPS |
| 3079_402.7078 | 1.32928821 | Control |
| 1305_230.1545 | 1.328676557 | Control |
| 902_200.1763 | 1.328456436 | Control |
| 992_208.1448 | 1.327889389 | MPS |
| 3933_552.3163 | 1.32763952 | Control |
| 385_154.1342 | 1.327500823 | MPS |
| 1343_233.1399 | 1.327448906 | Control |
| 2181_314.1964 | 1.326657912 | Control |
| 1116_217.0976 | 1.326315474 | MPS |
| 378_154.0865 | 1.325788021 | MPS |
| 761_190.0866 | 1.325189023 | Control |
| 2301_325.2238 | 1.324725859 | Control |
| 1907_290.1965 | 1.323920649 | Control |
| 4179_683.4244 | 1.323866331 | Control |
| 1849_286.2015 | 1.323746893 | Control |
| 597_175.1233 | 1.323721081 | MPS |
| 1520_249.1389 | 1.323196274 | Control |
| 382_154.1229 | 1.323064108 | Control |
| 1588_256.1658 | 1.322340565 | Control |
| 315_148.076 | 1.322128513 | Control |
| 1239_227.1029 | 1.322113949 | Control |
| 1451_244.1448 | 1.321983434 | Control |
| 2581_347.1677 | 1.321350247 | Control |
| 419_158.0814 | 1.321316086 | Control |
| 311_147.092 | 1.320721872 | Control |
| 529_168.1134 | 1.320332865 | Control |
| 2384_331.1907 | 1.319820846 | Control |
| 1160_220.1698 | 1.317905442 | MPS |
| 1981_297.2424 | 1.317671115 | MPS |
| 1412_241.1337 | 1.317533164 | Control |
| 2437_335.1718 | 1.317521219 | Control |
| 2405_333.0629 | 1.317397866 | Control |
| 831_195.1134 | 1.316882114 | Control |
| 1480_246.1601 | 1.316297575 | Control |
| 441_159.9694 | 1.316076842 | Control |
| 497_165.1025 | 1.315459377 | Control |
| 30_101.0598 | 1.315370542 | Control |
| 1536_251.1505 | 1.314903857 | Control |
| 289_143.1183 | 1.314560132 | Control |
| 2996_392.2284 | 1.313972837 | MPS |
| 2951_385.2587 | 1.313725506 | Control |
| 4268_828.6051 | 1.313463448 | Control |
| 1156_220.1335 | 1.312970971 | Control |
| 392_155.1069 | 1.312941051 | Control |
| 2168_313.1874 | 1.312339987 | Control |
| 823_194.1657 | 1.311130101 | Control |
| 826_194.1658 | 1.311114073 | Control |
| 1162_220.1811 | 1.309593232 | Control |
| 669_181.1702 | 1.309211306 | Control |
| 2284_324.2176 | 1.308506759 | Control |
| 613_177.0662 | 1.308256874 | MPS |
| 127_122.0967 | 1.307612868 | Control |
| 3166_414.2718 | 1.307148181 | Control |
| 1115_217.0975 | 1.30707864 | MPS |
| 1825_284.1761 | 1.30692119 | MPS |
| 2254_320.2074 | 1.306368788 | Control |
| 1427_242.1501 | 1.306295019 | Control |
| 4246_776.4738 | 1.306077806 | Control |
| 2932_383.2908 | 1.305927569 | MPS |
| 1125_217.1702 | 1.305842311 | Control |
| 2588_347.2082 | 1.30487947 | Control |
| 1107_216.1388 | 1.303982393 | Control |
| 1015_209.1651 | 1.30386771 | Control |
| 1347_234.1238 | 1.303507618 | Control |
| 1671_265.1173 | 1.303349281 | Control |
| 1574_255.1856 | 1.303076668 | Control |
| 655_181.0862 | 1.302922668 | Control |
| 1417_241.17 | 1.302855764 | Control |
| 1350_234.1491 | 1.301976598 | Control |
| 997_209.0925 | 1.301675164 | Control |
| 2599_348.2023 | 1.301409492 | MPS |
| 587_174.103 | 1.30120124 | Control |
| 206_133.1015 | 1.301072221 | Control |
| 566_172.0971 | 1.300912783 | Control |
| 1106_216.1386 | 1.29972759 | Control |
| 3280_436.2544 | 1.299607803 | Control |
| 1080_214.1342 | 1.298978191 | MPS |
| 1038_211.1082 | 1.298846259 | Control |
| 2793_366.3216 | 1.298789679 | MPS |
| 2659_354.2141 | 1.297743954 | Control |
| 229_137.0714 | 1.297656152 | Control |
| 1191_223.1809 | 1.297619834 | Control |
| 1110_216.1498 | 1.297493553 | Control |
| 866_197.129 | 1.297101447 | Control |
| 2868_376.2334 | 1.296259627 | Control |
| 2464_337.2238 | 1.294882414 | Control |
| 1079_214.1232 | 1.294770015 | Control |
| 1552_253.1297 | 1.294330854 | MPS |
| 1072_213.1599 | 1.294166008 | Control |
| 595_175.0871 | 1.29378168 | MPS |
| 1996_299.1501 | 1.293443979 | Control |
| 1846_286.1767 | 1.29242765 | Control |
| 1424_242.1501 | 1.292060182 | Control |
| 1499_247.8848 | 1.29194836 | Control |
| 3111_407.241 | 1.291679994 | MPS |
| 96_114.0916 | 1.291572048 | Control |
| 1869_287.2081 | 1.29093146 | MPS |
| 1005_209.1288 | 1.290893947 | Control |
| 999_209.1083 | 1.290863631 | Control |
| 1321_231.1706 | 1.290729625 | Control |
| 234_137.0965 | 1.290624998 | Control |
| 1902_290.16 | 1.290172778 | Control |
| 1924_291.2297 | 1.289490204 | Control |
| 1652_262.1655 | 1.289206819 | MPS |
| 3549_483.2615 | 1.289151261 | Control |
| 1850_286.2015 | 1.288458009 | MPS |
| 1074_213.1852 | 1.287652108 | Control |
| 4186_688.4212 | 1.28761736 | Control |
| 1395_239.1499 | 1.286597397 | Control |
| 1174_222.149 | 1.285760958 | Control |
| 1730_272.1857 | 1.285741914 | Control |
| 1059_213.1025 | 1.283735948 | MPS |
| 218_136.0761 | 1.282648667 | Control |
| 2144_311.1852 | 1.282094162 | MPS |
| 1069_213.1392 | 1.281536451 | MPS |
| 224_136.1125 | 1.281372369 | Control |
| 1751_274.177 | 1.28111008 | Control |
| 1614_258.2066 | 1.279687472 | Control |
| 1519_249.1389 | 1.279417227 | MPS |
| 1449_243.1858 | 1.279238464 | Control |
| 2542_344.2294 | 1.278985165 | Control |
| 2912_380.2284 | 1.278579589 | Control |
| 2894_378.2491 | 1.277297724 | Control |
| 709_185.1079 | 1.27661174 | Control |
| 1229_226.1549 | 1.275861258 | Control |
| 2157_313.0749 | 1.275147582 | Control |
| 1384_238.1336 | 1.274368487 | MPS |
| 3308_439.2667 | 1.273934283 | Control |
| 462_162.0915 | 1.273128408 | Control |
| 63_108.081 | 1.272960008 | Control |
| 16_94.0652 | 1.272896884 | MPS |
| 2671_355.2595 | 1.271488811 | MPS |
| 558_171.1495 | 1.271471614 | Control |
| 789_192.0772 | 1.270364988 | MPS |
| 2694_357.2753 | 1.270229082 | MPS |
| 1478_246.1339 | 1.269678109 | Control |
| 889_199.1446 | 1.26905984 | Control |
| 1199_224.1396 | 1.268445254 | Control |
| 1440_243.1127 | 1.267934627 | Control |
| 1725_272.1496 | 1.266250841 | Control |
| 17_94.0653 | 1.265763161 | Control |
| 1988_298.1918 | 1.265555007 | Control |
| 3251_430.3166 | 1.26512058 | MPS |
| 674_182.1294 | 1.264610731 | Control |
| 942_204.1386 | 1.264267909 | MPS |
| 2563_346.1652 | 1.264021155 | Control |
| 244_138.103 | 1.26326724 | MPS |
| 1445_243.1491 | 1.262891466 | Control |
| 968_207.1131 | 1.262766748 | Control |
| 2478_339.142 | 1.262625064 | Control |
| 1687_267.232 | 1.261907477 | Control |
| 2040_302.1719 | 1.261460011 | Control |
| 3209_422.3841 | 1.261208954 | Control |
| 1356_235.1232 | 1.260920913 | Control |
| 293_144.1022 | 1.260908353 | Control |
| 1153_220.1084 | 1.260189372 | Control |
| 1893_289.1804 | 1.259710304 | Control |
| 4050_607.3201 | 1.259079416 | Control |
| 784_191.1547 | 1.259043726 | MPS |
| 3390_455.2882 | 1.258884794 | MPS |
| 879_199.1082 | 1.258691574 | Control |
| 2118_308.295 | 1.258527228 | Control |
| 3807_526.3005 | 1.258283773 | MPS |
| 1444_243.1492 | 1.257980291 | Control |
| 2672_355.2822 | 1.257897342 | Control |
| 758_189.1238 | 1.257454232 | Control |
| 1919_290.2691 | 1.256826316 | Control |
| 1205_225.1123 | 1.256377779 | Control |
| 254_139.1234 | 1.255962663 | Control |
| 1526_250.1185 | 1.255869422 | Control |
| 4039_600.3688 | 1.255729419 | Control |
| 1758_275.1498 | 1.255127339 | Control |
| 922_202.1232 | 1.254720077 | MPS |
| 954_205.1703 | 1.254700959 | Control |
| 510_166.0866 | 1.254324501 | MPS |
| 1307_230.1653 | 1.254058819 | Control |
| 231_137.0714 | 1.254010188 | MPS |
| 2482_339.2032 | 1.253988905 | Control |
| 982_208.0973 | 1.253775058 | MPS |
| 1580_255.2321 | 1.253753461 | Control |
| 963_206.1543 | 1.253464755 | Control |
| 4078_623.4533 | 1.253155497 | Control |
| 1208_225.1236 | 1.252803138 | Control |
| 3721_511.3754 | 1.252793279 | Control |
| 2015_300.2022 | 1.252461524 | Control |
| 499_165.1025 | 1.251761058 | MPS |
| 1935_293.1377 | 1.251448229 | Control |
| 861_197.108 | 1.251243013 | Control |
| 2119_309.1315 | 1.250916484 | Control |
| 1234_226.1802 | 1.250905381 | Control |
| 4115_645.4663 | 1.250169267 | Control |
| 931_203.1547 | 1.24974425 | Control |
| 1374_237.1386 | 1.24906217 | Control |
| 2299_325.2237 | 1.248993286 | Control |
| 1418_241.1701 | 1.248451949 | Control |
| 3258_432.2232 | 1.248402115 | Control |
| 1743_273.1698 | 1.248396583 | Control |
| 1211_225.1389 | 1.248374014 | Control |
| 1165_221.1289 | 1.247743665 | Control |
| 1603_257.165 | 1.24705954 | Control |
| 1530_250.1549 | 1.246757859 | Control |
| 1184_223.1233 | 1.246613223 | Control |
| 1610_258.16 | 1.24563604 | Control |
| 622_177.1391 | 1.245077334 | Control |
| 2372_330.1917 | 1.244497446 | Control |
| 980_207.186 | 1.244090645 | Control |
| 1340_233.1286 | 1.243967888 | Control |
| 945_204.1499 | 1.243336519 | Control |
| 1375_237.1387 | 1.243263574 | Control |
| 2812_369.2134 | 1.243024917 | MPS |
| 1531_250.1551 | 1.24298617 | Control |
| 3952_557.3486 | 1.242853994 | MPS |
| 1076_214.0979 | 1.242688766 | Control |
| 2450_336.2023 | 1.242596715 | Control |
| 979_207.1859 | 1.242525688 | Control |
| 1539_251.1542 | 1.242512754 | Control |
| 2044_302.1966 | 1.242210756 | MPS |
| 3436_464.2855 | 1.240900427 | Control |
| 3920_549.2681 | 1.240804052 | Control |
| 2274_323.2079 | 1.240104436 | Control |
| 349_151.112 | 1.239311788 | Control |
| 2275_323.2081 | 1.238666265 | Control |
| 716_185.154 | 1.237825505 | MPS |
| 1555_253.1695 | 1.237772022 | Control |
| 1357_235.1233 | 1.23592202 | Control |
| 312_147.0921 | 1.234941653 | Control |
| 442_159.9694 | 1.233814217 | MPS |
| 848_196.1086 | 1.233674954 | Control |
| 4231_732.4473 | 1.233283303 | Control |
| 1161_220.1698 | 1.233245384 | MPS |
| 3224_425.1859 | 1.233243879 | Control |
| 1967_296.295 | 1.23309456 | Control |
| 1492_247.1442 | 1.232808073 | MPS |
| 2889_378.2127 | 1.232631693 | MPS |
| 1386_238.155 | 1.232298884 | Control |
| 291_144.0481 | 1.232237785 | Control |
| 1067_213.139 | 1.231404633 | Control |
| 687_183.0924 | 1.23058246 | Control |
| 3437_464.2855 | 1.23055166 | Control |
| 3076_402.2853 | 1.230117259 | Control |
| 28_100.0758 | 1.23001324 | MPS |
| 3717_511.2525 | 1.229784422 | Control |
| 15_90.9767 | 1.229266286 | Control |
| 3932_552.316 | 1.228776835 | Control |
| 801_193.0977 | 1.22873264 | Control |
| 955_205.1703 | 1.228676641 | MPS |
| 3516_478.3011 | 1.228524817 | Control |
| 1711_270.1817 | 1.228427759 | MPS |
| 1185_223.1235 | 1.228132093 | MPS |
| 1255_227.1545 | 1.227702813 | MPS |
| 1632_260.161 | 1.227619391 | MPS |
| 685_183.0923 | 1.226978523 | Control |
| 4111_644.3951 | 1.226751483 | Control |
| 1155_220.1335 | 1.226601749 | Control |
| 2685_356.3525 | 1.226596706 | Control |
| 1434_242.1865 | 1.226374675 | Control |
| 525_167.1433 | 1.226153125 | Control |
| 862_197.1081 | 1.226125658 | Control |
| 1551_253.1184 | 1.226118112 | Control |
| 1127_218.1179 | 1.226072258 | Control |
| 507_165.139 | 1.225579971 | Control |
| 1538_251.1541 | 1.225499646 | Control |
| 1297_229.155 | 1.225457132 | Control |
| 1091_215.1181 | 1.225430384 | Control |
| 2692_357.2135 | 1.225356112 | Control |
| 1040_211.1234 | 1.223981029 | Control |
| 1269_228.1343 | 1.223409587 | Control |
| 1396_239.1542 | 1.223034393 | MPS |
| 2600_348.2024 | 1.222839967 | Control |
| 1679_266.1613 | 1.222773824 | MPS |
| 1299_229.1702 | 1.222747465 | Control |
| 500_165.1026 | 1.222645443 | Control |
| 3469_469.3274 | 1.221157554 | Control |
| 1429_242.1656 | 1.22113455 | Control |
| 2182_314.1965 | 1.220520009 | Control |
| 2155_312.2302 | 1.220263112 | Control |
| 2481_339.2028 | 1.219734084 | Control |
| 1385_238.1437 | 1.219644079 | MPS |
| 790_192.1023 | 1.218768785 | Control |
| 1210_225.1349 | 1.218735394 | Control |
| 2137_311.1471 | 1.218585823 | Control |
| 1392_239.1394 | 1.218209712 | Control |
| 728_186.0919 | 1.218208456 | Control |
| 3668_501.2437 | 1.218170988 | MPS |
| 2853_374.2178 | 1.217938385 | Control |
| 1124_217.1702 | 1.217855526 | Control |
| 2154_312.1962 | 1.21739271 | Control |
| 1273_228.1595 | 1.216326379 | Control |
| 1041_211.1236 | 1.215833245 | Control |
| 2552_345.1447 | 1.215665774 | Control |
| 1595_256.2636 | 1.215637842 | MPS |
| 1258_227.1644 | 1.215582991 | MPS |
| 1349_234.134 | 1.215479175 | Control |
| 1659_263.1544 | 1.215273985 | Control |
| 1500_247.8848 | 1.214722721 | Control |
| 2597_348.2022 | 1.214502019 | Control |
| 1237_226.9517 | 1.213841402 | Control |
| 668_181.134 | 1.212948844 | Control |
| 33_101.0711 | 1.212788683 | Control |
| 854_196.1338 | 1.212555012 | MPS |
| 1831_284.3314 | 1.212521072 | Control |
| 1126_217.1703 | 1.211954429 | Control |
| 2092_306.1915 | 1.211941695 | Control |
| 998_209.1081 | 1.211625755 | Control |
| 3551_483.3432 | 1.211210224 | Control |
| 35_102.0915 | 1.210492746 | Control |
| 5_88.0758 | 1.210180903 | MPS |
| 1293_229.1337 | 1.209352133 | Control |
| 2650_352.3059 | 1.209113454 | Control |
| 957_206.0929 | 1.208414135 | Control |
| 1149_219.1497 | 1.208221332 | MPS |
